# Secondary analyses of sex differences in attention improvements across three clinical trials of a digital therapeutic in children, adolescents, and adults with ADHD

**DOI:** 10.1186/s12889-024-18597-5

**Published:** 2024-04-29

**Authors:** Jessica E. Flannery, Stephen P. Hinshaw, Scott H. Kollins, Caitlin A. Stamatis

**Affiliations:** 1 Akili Interactive Labs, Boston, MA USA; 2 Department of Psychology, University of California, Berkeley, CA USA; 3 Department of Psychiatry and Behavioral Sciences, University of California, San Francisco, CA USA; 4 Department of Psychiatry, Duke University School of Medicine, Durham, NC USA; 5 Department of Preventive Medicine, Northwestern University Feinberg School of Medicine, Chicago, IL USA

**Keywords:** Digital health, ADHD, Attention, Sex differences

## Abstract

**Background:**

Attention-deficit/hyperactivity disorder (ADHD) remains underdiagnosed and undertreated in girls. Inattentive symptoms, often predominant in girls with ADHD, represent a key driver of impairment and often persist into adulthood. AKL-T01 is a regulated digital therapeutic targeting inattention. We examined potential sex differences in the efficacy of AKL-T01 in three separate trials for 1) children, 2) adolescents, and 3) adults.

**Methods:**

We conducted secondary analyses of clinical outcomes by sex in three AKL-T01 randomized clinical trials in ADHD (n1 = 180 children 30.6% female, M(SD) age = 9.71 (1.32); n2 = 146 adolescents; 41.1% female, M(SD) age = 14.34 (1.26); n3 = 153 adults; 69.9% female, M(SD) age = 39.86 (12.84)). Active treatment participants used AKL-T01 for 25 min/day over 4–6 weeks. Primary outcomes included change in attention on the Test of Variables of Attention (TOVA) and symptom change on the clinician-rated ADHD Rating Scale (ADHD-RS). To evaluate study hypotheses, we conducted a series of robust linear regressions of TOVA and ADHD-RS change scores by sex, adjusting for baseline scores.

**Results:**

In children, girls demonstrated greater improvement in objective attention relative to boys following AKL-T01 (TOVA Attentional Composite Score; Cohen’s *d* = .36 and Reaction Time Mean Half; Cohen’s *d* = .54), but no significant sex differences in ADHD rating scale change. We did not observe significant sex differences in outcomes in the adolescent or adult trials. Limitations include binary sex categorization and slight study design variation across the three samples.

**Conclusion:**

AKL-T01 might notably improve attentional functioning in girls with ADHD relative to boys. Objective attention measures may be particularly important in the assessment of attentional improvement in childhood, given known gender biases in ADHD symptom reporting. We emphasize the importance of considering sex and gender-specific factors in ADHD treatment evaluation.

**Trial registrations:**

STARS ADHD CHILD: ClinicalTrials.gov ID NCT03649074;

STARS ADHD ADOLESCENT: ClinicalTrials.gov ID NCT04897074;

STARS ADHD ADULT: ClinicalTrials.gov ID NCT05183919.

**Supplementary Information:**

The online version contains supplementary material available at 10.1186/s12889-024-18597-5.

## Background

Although historically understood as a disorder that predominantly affects young boys, attention deficit/hyperactivity disorder (ADHD) is a neurodevelopmental disorder that typically persists across the lifespan and affects individuals across sexes and genders [1, 2]. Notably, given the dearth of literature differentiating gender from sex differences within ADHD, we predominantly use gender descriptors below, unless describing a sex-specific difference, while acknowledging that there are unique gender and sex contributions to ADHD presentations. Indeed, prevalence rates for ADHD remain higher for boys than girls, particularly in childhood [3]. However, there is mounting evidence that ADHD affects girls and women and at higher rates than previously understood [3, 4]. Despite the increased awareness of ADHD in girls, ADHD remains underdiagnosed and undertreated in girls and women, with relatively less research evidence on treatment efficacy for girls vs. boys [4].

### Manifestation of symptoms

Symptom presentation is an important contributor to the underrepresentation of girls and women in ADHD. Compared to boys, girls are more likely to present with the inattentive presentation of ADHD than either the hyperactive/impulsive or combined presentations [4]. Often salient are difficulties in paying attention, considerable disorganization, and low frustration tolerance, as well as underlying deficits in executive functions. Such inattentive symptoms often go unnoticed because they result in far less disruption than hyperactive-impulsive symptoms [4], often resulting in a misdiagnosis of an internalizing disorder such as anxiety or depression [3]. Further, inattentive symptoms represent a key driver of impairment, often persisting into adulthood [3] and predicting a range of later impairments, including issues with substance use and self-harm [5, 6]. Despite the impact of inattentive symptoms, especially for girls, they are less understood and less often the primary target of interventions [3, 4]. Understanding inattention-targeted interventions is particularly important to bridge the gap in understanding how to improve functioning in girls and women with ADHD.

### Prevalence across age

Sex and gender differences in the prevalence of ADHD vary across the lifespan. Although there is a 1.5–2.5 times higher prevalence of ADHD in boys relative to girls [4], these discrepancies are higher in childhood and become more similar across sexes and genders by mid-adulthood [1]. Again, symptom presentation differences may influence the change in sex and gender differences across age. That is, girls are more likely to display inattentive symptoms that persist into adulthood, whereas on average, boys are more likely to display hyperactive symptoms that often diminish in adulthood [4]. Note that such changes may relate, at least in part, to informant reports: that is, adults typically report their own symptoms. For example, parent and teacher reports of ADHD symptoms are more likely to overestimate boys’ symptoms of ADHD, but informant reports tend to underestimate girls’ symptoms of ADHD [7]. It is critical to understand how interventions for ADHD may differentially impact boys and girls, particularly in childhood where there are the greatest sex and gender discrepancies in symptom ratings.

### Sex & gender differences in treatment

Despite the increased awareness of girls and women with ADHD, girls and women have still traditionally been underrepresented in studies of ADHD and its treatment. Because of the historically prominent focus of boys with ADHD, medication and therapy studies have traditionally recruited samples with a male bias, oftentimes disproportionate to the expected ratio of male:female ADHD rates [1, 8, 9]. Consequently, less is known about the impacts and appropriate dosage of medication and therapy for females with ADHD. It has been reported that females with ADHD are significantly less likely to be prescribed ADHD medication than males with ADHD [1, 7]. Given known sex differences in ADHD neuropsychological functioning and brain function [10], it’s important to tease apart if there are sex differences in ADHD treatment efficacy. Additionally, traditional behavioral interventions for ADHD primarily target disruptive behaviors (e.g., Parent Child Interaction Therapy [11], Incredible Years [12]), a common profile for boys with ADHD. In general, women are more likely to seek mental health care services [13, 14], but this trend differs by mental health service and provider. Girls with ADHD are less likely to be referred for and receive treatment for ADHD than boys [3, 15, 16]. It is critical to explicitly identify potential sex-related differences in the design of effective interventions and clinical care for ADHD.

### AKL-T01 intervention

Given existing barriers to accessing gold-standard treatments, digital health interventions represent a promising means of expanding access to care for individuals with ADHD and families. AKL-T01 is a regulated digital therapeutic to improve attentional functioning for individuals with primarily inattentive or combined-type ADHD and demonstrated attentional impairment. AKL-T01 targets inattention through a bottom-up approach aimed to enhance cognitive processes underlying attention [17]. As a digital treatment, AKL-T01 is downloaded directly to a smart device, reducing barriers to in-person treatment. AKL-T01 is effective across children, adolescents, and adults [17] (Stamatis et al., *under review*). As a treatment that directly targets inattention, AKL-T01 provides an opportunity to elucidate the impact of potential sex and/or gender-related differences in an intervention specifically designed to target symptoms of particular relevance to girls and women with ADHD.

### Rationale and current study

Overall, there is a critical need to understand sex and gender differences regarding ADHD (a) with specificity for treatment of inattention and (b) across ages. In the present study, we examined sex differences in the efficacy of AKL-T01 in three separate clinical trials: 1) children, 2) adolescents, and 3) adults.

## Methods

### Overview

We conducted a secondary analysis of clinical outcomes by sex in children, adolescents, and adults from three trials of AKL-T01 (n1 = 180 children; 30.6% female, M(SD) age = 9.71 (1.32)9.71; n2 = 146 adolescents; 41.1% female, M(SD) age = 14.34 (1.26); n3 = 153 adults; 69.9% female, M(SD) age = 39.86 (12.84)). For full study details see Kollins et al. [17] and Study Protocols (Supplement). All participants had a diagnosis of ADHD (combined or inattentive presentation according to the Diagnostic and Statistical Manual of Mental Disorders, Fifth Edition (DSM-5) [18] as confirmed by MINI-Kid version 7.0.2 [19] or MINI for Attention-Deficit/Hyperactivity Disorders Studies (Adult) version 7.0.2 [20], as well as high inattention per a baseline score ≤ -1.8 on the Test of Variables of Attention (TOVA) [21], a computerized, FDA-cleared continuous performance task objectively measuring attention. For adults, diagnosis was further confirmed with a ADHD Rating Scale (RS)‑IV [22] score ≥ 24. All participants had a full scale IQ score ≥ 80 (as assessed by KBIT-II) [23, 24] and absence of any medical condition that could affect study participation or potentially confound study assessments. Notably, this study is described in terms of "sex" differences because the data includes a binary sex (male/female) variable.

All trials were conducted in compliance with the Institutional Review Board (IRB) regulations stated in Title 21 of the US Code of Federal Regulations (CFR), Part 56, Good Clinical Practice (GCP) regulations and guidelines, and all applicable local regulations. The trials were approved by each site’s institutional review board (WIRB-Copernicus Group). Written informed consent or assent was obtained from all participants, consent from parents or legally authorized representatives and assent for children and adolescents, respectively, and as appropriate given the participants' ages. IRB-approved forms containing a detailed description of the study treatment, study procedures, and risks were provided to participants and caregivers. For more details on the three trials inclusion and exclusion criteria, see the Study Protocols in the Supplementary Materials.

### Procedures

At baseline, participants received an iPad Mini with AKL-T01 software and completed training to learn proper device and software usage.

At the screening and exit visits, participants were assessed via validated measures of cognitive functioning (TOVA) and clinical symptoms (see “Outcomes”). Adolescent and adult participants were instructed to avoid caffeine for 4–6 hours prior to the visit. At the investigator’s discretion, participants may also have been instructed to delay that day’s ADHD medication until after completion of the TOVA at both screening and exit visits to avoid unintentional impact of ADHD medication on TOVA performance. For full study details see Kollins et al. [17] and Study Protocols (Supplement).

Adolescent and adult participants completed AKL-T01 treatment in combination with their previously established stable ADHD treatment regimen (which could include stimulant medications, other psychoactive medications, and nonpharmacological therapies), and keep their previously-established treatment stable for 4 + weeks. Children completed a stimulant wash out 3–7 days prior to enrollment and remained off medication throughout the study. Recommended treatment with AKL-T01 involved playing 6 to 8 missions per day (approximately 25 min) for at least 5 days per week for 4 weeks [6 weeks for adults]. Compliance was monitored electronically, and the software generated automatic reminders to play. The treatment automatically locked after the allocated maximum number of daily missions, and no further play was permitted until the next calendar day. Participation was determined by the absence of any medical condition as well as any concomitant psychiatric conditions based on clinical judgment, but not limited based on concomitant medications apart from ADHD drugs.

### Intervention

**Fig. 1:**
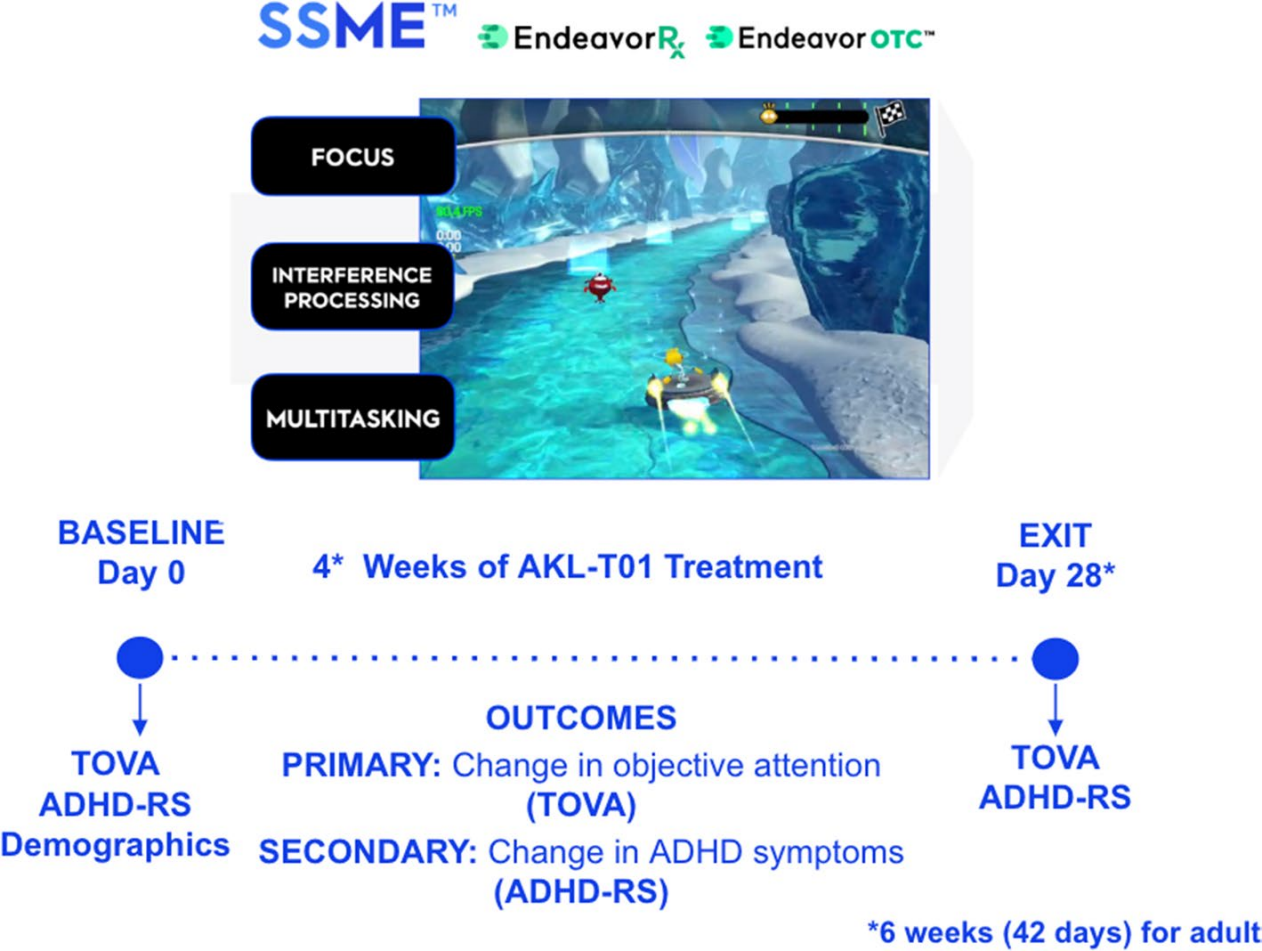
AKL-T01 intervention. The AKL-T01 intervention (EndeavorRx® for child and adolescent populations; EndeavorOTC® for adults) is a digital therapeutics indicated to improve attentional functioning for individuals with primary inattentive or combined-type ADHD and demonstrated attentional impairement. AKL-T01 uses a selective stimulus management engine (SSME) to adapt difficulty in real time to the individual patient. Across all three trials, participants completed the Attention-Deficit/Hyperactivity Disorder-Rating Scale (ADHD-RS) and the Test of Variables of Attention (TOVA) at baseline and again at the end of treatment (day 28 for the child and adolescent samples, and day 42 for adults)

AKL-T01 is a digital therapeutic built using a proprietary algorithm (Selective Stimulus Management Engine [SSME™]) designed to train interference management at an adaptive and personalized high degree of difficulty (See Fig. 1). Interference was instantiated through a video game-based interface displaying two tasks done in parallel (multitasking): a perceptual discrimination targeting task in which users responded to the stimulus targets and ignored the distractors (similar to a Go-No-Go task), and a sensory motor navigation task in which users continuously adjusted their location to interact with or avoid positional targets. Performance in each task was assessed during single and multitask conditions. The adolescent and adult samples were single armed studies. The child sample was a randomized control trial (see Supplemental for details of all study protocols). For purposes of this study question, control participants were not included in the analyses.

### Outcomes

#### Baseline characteristics and compliance

To understand baseline sex differences in our samples, we assessed if there were sex differences in age, number of completed sessions, and baseline objective attention and clinician-report ADHD measures. We also assessed if there were significant differences in the number of males and females in each sample. In the adult sample, Given the historically higher prevalence of these comorbid diagnoses for women [4], we also explored if there were baseline differences in self-reported comorbid anxiety and depression diagnoses in the adult sample. We did not have complete data on comorbidities to assess these differences in our child and adolescent samples.

#### Objective attention change score

We assessed sex differences in change (study day 1 to study day 28 [child and adolescent] or day 42 [adult]) on the TOVA-ACS^1^ (attentional composite score), with norms relative to both ADHD and non-clinical samples [21]. The TOVA ACS is a composite score of the sum of three scores: reaction time mean Half-1 (highly infrequent targets; RTMH), RT variability total (both halves; Variability), and d-prime Half-2 (highly frequent targets; d’prime). The clinical cutoff on the ACS is zero, with scores below zero frequently observed in samples with ADHD and scores above zero typical of non-clinical samples. All TOVA scores were standardized prior to analysis. Positive changes in the ACS and its subscales indicate improvement in objective attentional functioning.

#### ADHD symptom change score

We also assessed sex differences in change (study day 1 to study day 28 or 42 [adult]) in the ADHD RS-5[child and adolescent]/RS-IV[adult] inattention scale and total scores. The ADHD RS-5 is a parent-reported, clinician-administered assessment of the child’s frequency of ADHD symptoms, consisting of 18 items rated on a Likert scale ranging from 0 (never or rarely) to 3 (very often). The ADHD RS-IV, with adult prompts, is a clinician-rated measure of ADHD symptoms. We examined both the inattention subscale and total scores at baseline and after treatment with AKL-T01. The inattention scale score is the sum of the 9 items that comprise the inattention scale, and the total score is the sum of all 18 items [22].

### Statistical analysis

All analyses were post-hoc, secondary to the primary aims of the three studies; however, endpoints of interest were kept identical to reduce any bias in measurement selection: TOVA-ACS and ADHD-RS total score. We also conducted exploratory analyses of sex differences in treatment response as measured by subscales of the ADHD-RS and TOVA, to further understand whether sex differences exist within specific components of attention. To further combat biased reporting of secondary analyses, analyses were interpreted in the context of multiple comparison correction, using false discovery rate (FDR) correction. As in the primary analyses, all analyses were conducted using a complete case analysis; no missing data were imputed. Analyses were completed separately for the three trials: in children, adolescents, and adults. Data across trials were not pooled due to known sex differences in ADHD across age and methodological differences between samples.

#### Baseline and compliance differences

Baseline sex differences within our sample were examined with a series of independent sample t-tests, or chi square tests for categorical variables. For baseline measurements that did not conform to normality assumptions, we performed a non-parametric alternative: Kruskal–Wallis test.

#### Primary analyses

For our primary analyses, we performed a series of linear regressions to assess sex differences in the change in attentional outcome, accounting for baseline scores, using R (R Core Team, 2021; R version 4.3.1 (2023–06-16) and RStudio (version 2023.9.1.494). The ADHD-RS responses were normally distributed, with no evidence of clear outliers; therefore, we used the standard linear regression (lm function in R) to evaluate sex differences in ADHD-RS response. For non-normally distributed data and/or data with potential outliers, multiple linear regressions were executed using the Robustbase package “lmrob” function” [25]. Robust linear regression provides more reliable estimates, robust to outliers and nonnormal data, by using an iteratively reweighted least squares method to assign weight to each data point [25]. Sensitivity analyses were performed to identify whether the results were sensitive to outliers, using 3SD outlier removal, and adjusting for age or number of sessions. We also conducted sensitivity analyses on the ADHD-RS models to identify whether the traditional linear regression results differed with the robust regression model. For statistically significant effects, Cohen’s *d* effect sizes were also calculated.

## Results

### Participants

#### Children

Of 857 children screened for eligibility, 348 patients were randomly assigned to receive AKL-T01 (*n* = 180) or control (*n* = 168) between July 15, 2016, and Nov 30, 2017. Participants were predominantly male (69.4%), White (73.9%) and non-Hispanic/Latino (77.2%; see Supplemental Table 3 for detailed breakdown), with a mean age of 9.71 years (SD = 1.32).

#### Adolescents

Of 526 participants screened, 162 were enrolled between July 29, 2021 and September 1, 2022. Fifteen participants (9.3%) discontinued the study. Participants were predominantly male (58.9%), White (77.4%) and non-Hispanic/Latino (81.5%; see Supplemental Table 3 for detailed breakdown), with a mean age of 14.3 years (SD = 1.26), and approximately half (49.3%) reported current stimulant use.

#### Adults

Of 440 participants screened, 221 were enrolled between November 9, 2021 and December 2, 2022. 75 participants (34%) discontinued the study. Participants were predominantly female (70%), White (77%) and non-Hispanic/Latino (85%; see Supplemental Table 3 for detailed breakdown), with a mean age of 39.9 years (SD = 12.84), and 60 (39%) reported current stimulant use.

### Sex differences in intervention sessions completed and age

There were no significant sex differences in the number of sessions of the intervention that were completed within the child, adolescent, or adult trials (child: *M(SD)female* 86.56 (22.78), *M(SD)male* 81.072 (32.109), *p* = 0.25; adolescent: *M(SD)female* 93.567 (53.618), *M(SD)male* 78.895 (41.827), *p* = 0.066; adult: *M(SD)female* 157.670 (101.803), *M(SD)male* 141.217 (82.090), *p* = 0.335). There were significantly more males than females in both of the child and adolescent trials, but significantly more females than males in the adult trial (See supplemental Table 1 for sex and age statistics).

### Sex differences in baseline measurements of attention and ADHD symptoms

#### Children

Kruskal–Wallis tests revealed significant sex differences in several baseline measurements. Boys had significantly lower (worse) baseline TOVA Attention Composite Score (ACS), and TOVA subscore variability (ACS: *p* = 0.045; Variability: *p* = 0.001; See Table 1). There were no significant sex differences in the baseline reaction time mean half (RTMH) or d'prime subscores (RTMH:*p* = 0.91; d’prime: *p* = 0.051; See Table 1). Boys also had significantly higher ADHD total scores (*p* = 0.02) but did not have statistically different inattention subscale scores (*p* = 0.66; See Table 1). Only the baseline difference in the TOVA variability subscore survived multiple comparison correction (*p* = 0.005, corrected).

**Table 1:** Baseline Sex Differences in Outcomes

Measure	Female	Male	*p* value
	M(SD)	M(SD)	(corrected)
Children	*n* = 55	*n* = 125	
Objective Outcomes			
ACS baseline	-4.58 (2.71)	-5.34 (3.07)	0.045 (0.07)
RHTH baseline	74.46 (21.04)	75.22 (21.06)	0.91
Variability baseline	58.16 (28.69)	42.94 (36.77)	0.001 (0.005)
d'prime baseline	71.89 (9.07)	74.68 (7.82)	0.05
Subjective Outcomes			
ADHD-RS total score baseline	37.27 (7.34)	39.77 (6.42)	0.02 (0.05)
ADHD-RS Inattention subscore	22.06 (3.55)	21.81(3.45)	0.66
Adolescents	*n* = 60	*n* = 86	
Objective Outcomes			
ACS baseline	-6.40 (4.72)	-4.781(2.74)	0.04 (0.08)
RHTH baseline	75.52 (27.89)	84.75 (19.98)	0.04 (0.08)
Variability baseline	33.97 (52.22)	47.44 (34.53)	0.1
d'prime baseline	67.49 (10.59)	69.09 (9.79)	0.33
Subjective Outcomes			
ADHD-RS total score baseline	30.33 (8.87)	32.31 (9.34)	0.2
ADHD-RS Inattention subscore	19.27 (5.42)	18.66 (4.86)	0.48
Adults	*n* = 107	*n* = 56	
Objective Outcomes			
ACS baseline	-7.08 (4.10)	-12.60 (10.54)	0.003 (0.008)
RHTH baseline	68.68 (30.30)	57.492 (50.258)	0.8
Variability baseline	32.81 (45.37)	-22.450 (109.992)	0.004 (0.008)
d'prime baseline	65.30 (17.07)	48.100 (44.89)	0.043 (0.058)
Subjective Outcomes			
ADHD-RS total score baseline	39.00 (7.33)	36.50 (7.39)	0.06
ADHD-RS Inattention subscore	22.10 (3.09)	21.11 (3.58)	0.08

Baseline Sex Differences in Outcomes across Children, Adolescents, and Adults

*P* values are shown corrected for multiple comparison (false discovery rate)

#### Adolescents

Kruskal–Wallis tests revealed significant sex differences in several baseline measurements; however, none survived comparison for multiple corrections. Girls had significantly lower (worse) baseline TOVA Attention Composite Score (ACS), and TOVA subscore reaction time mean half (RTMH)(ACS: *p* = 0.04; RTMH: *p* = 0.04; See Table 1). There were no significant sex differences in the baseline variability or d'prime subscores (variability: *p* = 0.097; d’prime: *p* = 0.33; See Table 1). There were no significant sex differences in baseline ADHD total and inattention subscale scores (total score: *p* = 0.20; inattention: *p* = 0.48; See Table 1).

#### Adults

Kruskal–Wallis tests revealed significant sex differences in several baseline measurements. Men had significantly lower (worse) baseline TOVA Attention Composite Score (ACS), and TOVA variability and d'prime subscores (ACS: *p* = 0.003; variability: *p* = 0.004; d’prime: *p* = 0.043; See Table 1). There were no significant sex differences in the baseline reaction time mean half (RTMH; *p* = 0.799; See Table 1). There were no significant sex differences in baseline ADHD total and inattention subscale scores (total score: *p* = 0.06; inattention: *p* = 0.08; See Table 1). Only sex differences in baseline TOVA ACS and variability scores survived multiple comparison correction (*p* = 0.008, corrected).

### Sex differences in outcomes

#### Children

##### *Objective measures of attention outcomes*

After statistically adjusting for baseline scores, there was a significant sex-specific difference in the change of TOVA-ACS scores and TOVA-RTMH scores from baseline to day 28 (See Table 2). Specifically, girls exhibited significantly greater improvement in TOVA-ACS (Cohen’s *d* = 0.36) and RTMH (Cohen’s *d* = 0.54) scores following the AKL-T01 intervention, compared to boys. These results were retained when covarying age and number of sessions or removing outliers +—3SD above or below the mean (*p*’s < 0.05). Also adjusting for baseline scores, there were no significant sex-specific differences in TOVA-RT variability or d’prime score (See Table 2).

##### *ADHD rating scale measures of outcomes*

After adjusting for baseline scores, there were no significant sex-specific differences in the ADHD-Rating Scale-5 (ADHD-RS-5) endpoint improvement from baseline to day 28 (See Table 2). This pattern remained the same when covarying for age and number of sessions, or removing outliers +—3SD above or below the mean (*p*’s > 0.05).

**Table 2:** Child Sex Differences in Outcomes

Model	Term	Estimate	std.error	Statistic	*p* value
ACS	(Intercept)	0.56	0.51	1.09	0.28
	Male	-0.93	0.46	-2.03	0.04
	ACS BL	-0.21	0.09	-2.33	0.02
RTMH	(Intercept)	32.18	5.26	6.12	< .001
	Male	-10.59	2.95	-3.6	< .001
	RTMH BL	-0.27	0.07	-4.06	< .001
Variability	(Intercept)	20.21	6.22	3.25	0
	Male	-4.97	3.89	-1.28	0.2
	Variability BL	-0.16	0.08	-2.03	0.04
d'prime	(Intercept)	23.94	6.78	3.53	< .001
	Male	0.03	1.3	0.02	0.98
	d'prime BL	-0.31	0.1	-3.27	0
ADHD-RS Total	(Intercept)	0.6	3.39	0.18	0.86
	Male	0.02	1.27	0.01	0.99
	Total Score BL	-0.17	0.09	-2.02	0.05
ADHD-RS inattention	(Intercept)	4.93	2.37	2.08	0.04
	Male	-0.17	0.78	-0.22	0.82
	Inattention BL	-0.38	0.1	-3.72	< .001

Child Sex Differences in Outcomes. Robust linear regressions, co-varying for baseline measurement

Results remain with sensitivty analyses for outlier removal, co-varing age and compliance (number of sessions completed)

#### Adolescent and Adult

##### Objective measures of attention outcomes

*For both adolescents and adults, *after adjusting for baseline scores, there were no significant sex-specific differences in changes scores for TOVA measurements from baseline to day 28 (adolescent; Table 3) or day 42 (adult; Table 4). This pattern remained the same when adjusting for age and number of sessions, or removing outliers +—3SD above or below the mean.

##### ADHD rating scale measures of attention outcomes

*For both adolescents and adults, *after adjusting for baseline scores, there were no significant sex-specific differences in the ADHD-Rating Scale (ADHD-RS) endpoint improvement from baseline to day 28 (adolescent; Table 3) or day 42 (adult; Table 4). This pattern remained the same when adjusting for age and number of sessions, or removing outliers +—3SD above or below the mean (*p*’s > 0.05).

**Table 3:** Adolescent Sex Differences in Outcomes

Model	Term	Estimate	std.error	Statistic	*p* value
ACS	(Intercept)	1.13	0.74	1.51	0.13
	Male	-0.47	0.65	-0.72	0.47
	ACS BL	-0.32	0.09	-3.63	< .001
RTMH	(Intercept)	48.66	5.79	8.41	< .001
	Male	-1.63	2.99	-0.54	0.59
	RTMH BL	-0.46	0.06	-7.57	< .001
Variability	(Intercept)	38.02	7.25	5.24	< .001
	Male	0	5.65	0	1
	Variability BL	-0.39	0.11	-3.59	< .001
d'prime	(Intercept)	5.33	7.33	0.73	0.47
	Male	-2.86	1.92	-1.49	0.14
	d'prime BL	0.03	0.11	0.25	0.81
ADHD-RS Total	(Intercept)	4.55	2.36	1.93	0.06
	Male	0.91	1.31	0.7	0.49
	Total Score BL	-0.31	0.07	-4.36	< .001
ADHD-RS inattention	(Intercept)	5.34	1.67	3.2	0
	Male	0.37	0.83	0.45	0.65
	Inattention BL	-0.45	0.08	-5.64	< .001

Adolescent Sex Differences in Outcomes. Robust linear regressions, covarying for baseline measurement

Results remain with sensitivty analyses for outlier removal, co-varing age and compliance (number of sessions completed)

**Table 4:** Adult Sex Differences in Outcomes

Model	Term	Estimate	std.error	Statistic	*p* value
ACS	(Intercept)	1.02	0.54	1.87	0.06
	Male	-0.28	0.86	-0.32	0.75
	ACS BL	-0.62	0.07	-8.82	< .001
RTMH	(Intercept)	79.08	5.27	15.01	< .001
	Male	4.12	2.61	1.58	0.12
	RTMH BL	-0.71	0.06	-11.41	< .001
Variability	(Intercept)	64.14	3.88	16.54	< .001
	Male	1.42	6.21	0.23	0.82
	Variability BL	-0.66	0.05	-12.45	< .001
d'prime	(Intercept)	29.82	6.42	4.65	< .001
	Male	-6.41	3.89	-1.65	0.1
	d'prime BL	-0.28	0.1	-2.97	0
ADHD-RS Total	(Intercept)	1.38	3.35	0.41	0.68
	Male	-0.72	1.35	-0.53	0.59
	Total Score BL	-0.25	0.08	-2.94	0
ADHD-RS inattention	(Intercept)	4.23	2.6	1.63	0.11
	Male	-0.65	0.82	-0.79	0.43
	Inattention BL	-0.42	0.12	-3.62	< .001

Adult Sex Differences in Outcomes. Robust linear regressions, covarying for baseline measurement

Results remain with sensitivty analyses for outlier removal, covaring age and compliance (number of sessions completed)

## Discussion

### Summary and implications of results

In a secondary analysis study, we examined sex-specific differences in the efficacy of a digital therapeutic for treating inattention in ADHD across three age groups from three trials: children (8–12 years old), adolescents (12–17 years old), and adults (18 + years old). In childhood, girls demonstrated greater improvement in objective attention metrics following the AKL-T01 intervention, compared to boys, but sex differences in attentional outcomes were not observed in the adolescent and adult samples. The observed differences in childhood showed a small to medium effect size, suggesting a nuanced sex-specific response to the intervention. Given the traditional underrepresentation of girls in treatment evaluations for ADHD, particularly in childhood, it is encouraging that girls not only benefit from an attention intervention in childhood but appear to potentially benefit even more than boys. The absence of a sex-difference in treatment efficacy for the adolescent and adult samples is promising given the persistence of inattention symptoms into adulthood [3], and consequent need for effective attention-targeted interventions across sexes and across the lifespan. These data shed greater light on an often understudied aspect of ADHD treatment development and provide greater insight into how inattention, a predominant symptom in girls and women with ADHD [4], responds to non-pharmacological interventions. These data suggest that despite expected baseline sexes differences in attentional measures of ADHD, in childhood, girls may particularly benefit from the AKL-T01 intervention, and across age groups both sexes demonstrate clinically meaningful changes in attention.

### Child sex differences in outcomes

Our data suggest that girls may experience significantly greater improvement in objective measures of attention (TOVA attentional composite score and reaction time subscore) following the AKL-T01 attentional intervention, as evident by a small-to-medium effect size. Girls are less likely to be diagnosed in childhood and more likely to display more symptoms of ADHD later in development than boys [26]. Consistent with these trends, boys in our sample had significantly higher baseline ADHD-RS scores than girls, and their symptoms remained significantly higher than girls at the end of treatment. Although the statistical phenomenon of regression to the mean would postulate that boys would be more likely to see an improvement in their scores than girls, we saw the opposite pattern. Namely, our data suggest that even if girls show a less impaired baseline presentation of inattentive symptoms in childhood, they still show significantly greater improvements in objective measures of attention compared to boys following the AKL-T01 intervention.

In contrast, we did not observe a significant sex difference in change scores on the ADHD-RS total score or the subscale of attention in our child sample. This pattern mirrors other studies that demonstrate discrepancies between objective and subjective measures of ADHD, particularly in childhood, which are likely to be influenced by gender biases in both symptom presentation and reporting [3, 4]. For example, parents are more likely to rate boys’ symptoms of ADHD higher than girls’ [7], which is consistent with our findings. This pattern has also been found within medication studies [27, 28]. The combination of results suggest the inclusion of objective measures of attention may be particularly important for assessing treatment efficacy across genders and sexes to reduce the impact of gender bias on outcome measures solely reliant on self or other informant-report ratings.

### Adolescent and adult sex differences in outcomes

We did not observe significant sex differences in objective measures of attention or the ADHD rating scale in either the adolescent or adult sample. Notably, both men and women showed clinically meaningful improvement in their attentional scores, suggesting that even though women showed a relatively less impaired attentional profile at baseline, AKL-T01 was equally helpful in improving attention. The lack of observed sex differences in treatment is consistent with prior literature indicating that sex differences in the rates of ADHD across the lifespan and the life course of ADHD symptomatology become more similar across age [1]. Taken together with the childhood findings, these results underscore the importance of accounting for age in understanding potential sex differences in ADHD intervention treatment response.

### Baseline sex differences across samples

There were no significant differences in the number of sessions completed within each age sample, suggesting sex differences are not due to differences in the intervention dose, or other baseline characteristics, such as motivation. As expected, men showed greater baseline impairment in childhood and adulthood than women. Consistent with the historically higher prevalence of anxiety and depression comorbid diagnoses for women [4], nearly 1/3rd of women self-reported at least one comorbid internalizing disorder (depression or anxiety), compared to less than 1/5th of men reporting a comorbid internalizing disorder. These data suggest that regardless of baseline severity of ADHD symptoms or internalizing comorbidities, AKL-T01 intervention was successful in improving attentional outcomes for both men and women.

### Limitations

These findings should be considered within the context of study limitations. This study was a secondary analysis of sex differences across three trials; therefore, it was not designed to optimize for assessing sex differences across age and may mean there were additional sex differences we were not powered to detect. Although our three samples had a significant overlap in inclusion and exclusion criteria, there were differences in their study designs that could have contributed to the differences we see between samples. For example, the adult intervention was 6 weeks, whereas the child and adolescent studies were 4 weeks. It is unknown how an additional 2 weeks of treatment may have influenced the observed sex differences in our pediatric samples. Importantly, in the studies, categorization was based on binary sex, which does not capture nuances of gender identity. This is a notable gap in ADHD literature and should be addressed with greater sample sizes to differentiate sex and gender effects of outcomes, given the known biological and sociological factors that can contribute to differences in ADHD symptom presentation. Further, our sample sizes were not equal between boys and girls, which is consistent with the diagnostic rates of ADHD for boys and girls, but again limited our power to evaluate potential sex differences in effects. Last, we did not have sufficient information regarding the different ADHD presentation types. It’s possible there were sex/gender differences in inattentive versus combined type presentations, which may have influenced findings.

### Future directions

Future studies should seek to identify nuances of comorbidities, gender identity, race, ethnicity, socioeconomic status, and cultural considerations that can influence gender presentations of ADHD, as well as caregivers’ perceptions of symptoms and related parenting practices that may influence ADHD presentation and outcome effects. It will also be important for future studies to tease apart sex-specific interactions with medication and intervention effects, as stimulant use was “washed-out” in our childhood sample, but not the other samples. This is notable because although AKL-T01 shows comparable efficacy in people on or off effective stimulant medication [29], boys are prescribed stimulants at higher rates than girls [1], which therefore may affect sex-specific differences in outcomes in the real world.

## Conclusion

These limitations notwithstanding, the present study offers insight into sex-specific differences in AKL-T01 treatment effects, specifically targeting inattention. This work underscores the importance of considering sex and gender-specific factors in the evaluation of ADHD treatments, in light of the relatively limited attention on treatment efficacy for girls, compared to boys. These data suggest that AKL-T01 provides an efficacious, non-pharmacological treatment option for both girls and boys with inattentive symptoms of ADHD. Future work should continue to disentangle potential contributing factors to these sex and/or gender effects and evaluate functional improvement in real world behaviors across sex, genders and age, ultimately contributing to improved clinical care and the well-being of individuals with ADHD, across sex, genders, and age.

### Supplementary Information

**Supplementary Material 1. **

**Supplementary Material 2. **

**Supplementary Material 3. **

**Supplementary Material 4. **

**Supplementary Material 5. **

## Data Availability

Investigators agree to share de-identified individual participant data, the study protocol, and the statistical analysis plan with academic researchers 6 months after publication, and following completion of a Data Use Agreement. Analysis scripts will be available on github. Proposals should be directed to medinfo@akiliinteractive.com.

## Footnotes

1 In the child study, the APS is referred to as the API (Attentional Performance Index), the original name for the composite score, but it is referencing the same measure.
