# Supplementary material for: Secondary analyses of sex differences in attention improvements across three clinical trials of a digital therapeutic in children, adolescents, and adults with ADHD
Source: BMC Public Health. 2024 Apr 29;24:1195. doi: 10.1186/s12889-024-18597-5 (PMC11057090; doi:10.1186/s12889-024-18597-5)
Supplement: Supplementary file 1 — Supplementary Material 1. [file 12889_2024_18597_MOESM1_ESM.zip › AKL-T01 Adolescent Study Protocol.pdf]

Protocol Number: Akili-051

Official Title: A single arm pivotal trial to assess the efficacy of AKL-T01, a novel digital intervention designed to improve attention, in adolescents, aged 13-17 years old, diagnosed with Attention Deficit Hyperactive Disorder (ADHD)

Short Title: Software Treatment for Actively Reducing Severity of ADHD in Adolescents (STARS-ADHD-Adolescents)

Version: 5.0

Date: April 25, 2022

Study Sponsor: Akili Interactive Labs, Inc.
Sponsor Address: 125 Broad Street, 4th Floor; Boston, MA 02110
Sponsor Representative: Vandana Menon, MD, PhD, VP Clinical Research

Protocol Approval Page

<u>Akili-051:</u> A single arm pivotal trial to assess the efficacy of AKL-T01, a novel digital intervention designed to improve attention, in adolescents, aged 13-17 years old, diagnosed with Attention Deficit Hyperactive Disorder (ADHD)

Version: 5.0 **Date of Issue:** April 25, 2022

We, the undersigned, have read, and approve this protocol and agree on its content.

SPONSOR REPRESENTATIVE	INVESTIGATOR
DocuSigned by: Vandana Menon EFA07A4EC9B440E...	
Signature	Signature
4/25/2022	
Date	Date
Vandana Menon, MD, PhD<br>VP Clinical Research	
Printed Name	Printed Name

Statement of Compliance

This trial will be carried out in accordance with International Conference on Harmonisation Good Clinical Practice (ICH GCP) and the following:

US Code of Federal Regulations (CFR) applicable to clinical studies (45 CFR Part 46, 21 CFR Part 50, 21 CFR Part 56, 21 CFR Part 312, and/or 21 CFR Part 812)

The protocol, informed consent forms, recruitment materials, and all participant materials will be submitted by the Investigator to the reviewing institutional review board (IRB) for review and approval. Approval of both the protocol and the consent form must be obtained before any participant is enrolled. Any amendment to the protocol will require review and approval by the IRB before the changes are implemented to the study. In addition, all changes to the consent form will be IRB approved; a determination will be made regarding whether a new consent needs to be obtained from participants who provided consent, using a previously approved consent form.

Protocol Version and Amendment Tracking

Version Number/Amendment	Approval Date
V 1.0 / Original	March 18, 2021
V 2.0 / Amendment 1	August 10, 2021
V 3.0 / Amendment 2	October 05, 2021
V 4.0 /Amendment 3	November 29, 2021
V 5.0/Amendment 4	April 25, 2022

Protocol Synopsis

Protocol Number	Akili-051
Protocol Title	A single arm pivotal trial to assess the efficacy of AKL-T01, a novel digital intervention designed to improve attention, in adolescents, aged 13-17 years old, diagnosed with Attention Deficit Hyperactive Disorder (ADHD)
Sponsor	Akili Interactive Labs, Inc.
Non-Significant Risk Medical Device Investigation	This study fulfills the requirements for a non-significant risk medical device investigation as:<br>• It does not meet any of the criteria set forth in 21 CFR 812.3(m)] to define a significant risk device<br>• Was previously determined by Institutional Review Boards to be non-significant device
Study Phase	Pivotal
Study Product	AKL-T01 Digital Treatment (AKL-T01)
Background	First-line treatment for Attention Deficit Hyperactive Disorder (ADHD) includes the use of stimulant medications, which is effective in reducing core symptoms of the disorder but also associated with well-documented side effects. In addition, there is recent evidence that pharmacological treatment may not show optimal benefits in some cognitive domains. Computerized cognitive training programs have shown some promise in improving working memory and attention in ADHD populations. Novel, cost-effective, non-pharmacological interventions for ADHD which are easy to implement and disseminate could be helpful for many participants given the limitations of other approved interventions.<br><br>AKL-T01 is a novel, highly immersive, digital therapeutic intervention which is currently FDA cleared as EndeavorRx™. EndeavorRx™ is a digital therapeutic indicated to improve attention function as measured by computer-based testing in children ages 8-12 years old with primarily inattentive or combined-type ADHD, who have a demonstrated attention issue. AKL-T01 is deployed on mobile devices (i.e., tablets) and incorporates adaptive, simultaneous cognitive tasks in a consumer-grade action videogame-based platform with high-quality graphics and reward mechanisms.

	AKL-T01 was studied in the Software Treatment for Actively Reducing Severity of ADHD as Adjunctive Treatment to Stimulant (STARS-ADHD) study; a multi-center, randomized, double-blind, active-controlled study comparing AKL-T01 treatment to the use of a digital control in 348 pediatric participants aged 8-12 years, diagnosed with ADHD and a measurable impairment in objective attention function (as indicated by the TOVA Attention Performance Index (API) ≤ -1.8) who were not taking ADHD medication. 180 participants were randomized into AKL-T01, and 168 into the control group and both groups were instructed to engage with their intervention for approximately 25minutes/day, 5days/week for 4 weeks. The study successfully demonstrated a statistically significant improvement (p=0.006) between AKL-T01 and control on the primary endpoint (TOVA-API [a composite measure of objective attention] change from Baseline to after 4-weeks of treatment). This improvement in objective attention (TOVA-API) in the treatment group is indicative of a clinically meaningful benefit for AKL-T01 participants and is supported by the results of the study secondary endpoints, which all consistently trended towards improvement.<br><br>A subsequent open-label study, STARS-Adjunct, included children both on and off ADHD stimulant medication, ages 8-14 years old, and found comparable effects in these two cohorts on ADHD-related impairment and ADHD symptoms. Children with measurable attention impairment at baseline comparable to STARS-ADHD (TOVA API ≤ -1.8), also showed improvement in objective attention function.<br><br>In both these studies, AKL-T01 demonstrated a favorable safety profile, with no serious adverse events observed.<br><br>This protocol will evaluate objective attention functioning and ADHD symptoms/impairments in adolescents between 13 and 17 years old, with a diagnosis of ADHD (combined or inattentive subtype), stably on or off ADHD medication, after 4-weeks of AKL-T01 treatment.
Study Design	Multi-center, unblinded, uncontrolled study
Study Objectives and Endpoints	1. The primary objective of this study is to evaluate the efficacy of AKL-T01 as determined by the change in a digitally assessed measure, Test of Variables of Attention (TOVA®) Attention Comparison Score (ACS), of sustained and selective attention, after 4 weeks of treatment with AKL-T01<br>2. A secondary objective of this study is to evaluate the change in ADHD symptoms, as determined by change in the ADHD Rating

	Scale-5 inattention sub-scale and total scale scores, after 4 weeks of treatment with AKL-T01<br>3. Exploratory objectives of this study are:<br>a. To evaluate the change in patient and caregiver perceived cognitive deficits as determined by the change in selected items from the PROMIS® Parent Proxy Item Bank v1.1 – Cognitive Function and the PROMIS® Pediatric Item Bank v1.0-Cognitive Function after 4 weeks of treatment with AKL-T01<br>b. To evaluate changes in functional impairment as determined by change in ADHD-Rating Scale 5 Home Version relationship with significant others, peer relationships, academic functioning, behavioral functioning, homework functioning and self-esteem domains<br>c. To evaluate changes in TOVA metrics other than ACS after 4 weeks of AKL-T01 treatment<br>d. To evaluate response rates for TOVA-ACS and ADHD RS-5 after 4 weeks of AKL-T01 treatment
Study Overview	Participants will have two on-site visits and an at-home intervention period:<br>1. Baseline visit (Day 1)<br>a. Confirm eligibility<br>b. Obtain informed consent<br>c. Provide training on the intervention<br>d. Obtain baseline data for study assessments<br>2. At-home treatment (Day 1-27)<br>a. Participants instructed to use the intervention at-home for approximately 25 minutes a day, 5 days a week<br>3. Exit/post-treatment visit (Day 28+3)<br>a. Post treatment assessments<br>b. Participant experience surveys
Inclusion/Exclusion Criteria	**Inclusion**<br>1. Adolescents between the ages of 13 and 17 years and 10 months at time of consent (must be under 18 years at study completion)<br>2. Confirmed diagnosis of ADHD combined or inattentive type, according to Diagnostic and Statistical Manual of Mental Disorders, Fifth Edition (DSM-5) as confirmed by MINI-Kid Version 7.0.2.<br>3. Stably on or off ADHD medication for ≥4 weeks prior to study enrollment and throughout the 4-week study

	4. Baseline visit score on the TOVA-ACS score ≤ -1.8<br>5. Access to and self-report of ability to connect wireless devices to a functional wireless network<br>6. Ability to follow written and verbal instructions (English) as assessed by the PI and/or study coordinator<br>7. Able to comply with all testing and study requirements<br>8. Estimated IQ score ≥80 as assessed by the Kaufmann Brief Intelligence Test, Second Edition (KBIT-II)<br>9. Patient assent and caregiver informed consent<br>10. Stably on or off psychoactive medications for ≥4 weeks prior to study enrollment and throughout the 4-week study<br>**<u>Exclusion</u>**<br>1. Current controlled or uncontrolled, comorbid psychiatric diagnosis that in the opinion of the Investigator may confound study data/assessments.<br>2. Participant is currently considered at risk for attempting suicide, has made a suicide attempt within the past year, or is currently demonstrating active suicidal ideation or self-injurious behavior, in the opinion of the Investigator based on the MINI-kid clinical interview.<br>3. Motor condition (e.g., physical deformity of the hands/arms) that prevents game playing as reported by the participant or observed by the Investigator.<br>4. Recent history (6 months prior to screening) of substance use disorder<br>5. History of seizures (excluding febrile seizures), significant tics, or a current diagnosis of Tourette's Disorder.<br>6. Known sensitivity to playing video games, such as photo-sensitive epilepsy, light-headedness, dizziness, nausea, or motion sickness.<br>7. Participation in a clinical trial within 3 months prior to screening.<br>8. Plans to initiate, or to make significant changes in frequency, of non-pharmacological behavioral therapy during the study<br>9. Color blindness as detected by Ishihara Color Blindness Test<br>10. Urine test positive for nicotine or marijuana<br>11. Any other medical condition that in the opinion of the Investigator may confound study data/assessments.<br>12. Previous exposure to Akili Products within the 6 months prior to study enrollment<br>13. Plans to initiate or to make significant changes in frequency or duration of non-pharmacological trainings with the aim to improve cognition by means of game or app-based cognitive trainings or neurofeedback, during the study

Treatment Regimen	Approximately 25 minutes/day for 5 days/week of AKL-T01 across the 4-week (28 day) treatment period.
Number of Sites	up to 20
Number of Participants	Up to 165 participants will be enrolled to achieve 135 completed participants
Duration of Study Participation	32 days: Baseline visit, 28-day treatment phase, and an Exit Visit on day 28 (+3) days.
Primary Endpoint	Change (Study Day 1 to Study Day 28) on the Attention Comparison Score, an overall composite score from TOVA 9 (TOVA-ACS). TOVA-ACS is equivalent to TOVA- Attention Performance Index (API).
Secondary Endpoint	Change (Study Day 1 to Study Day 28) in the ADHD Rating Scale-5 Home Version inattention scale and total scale scores
Exploratory Endpoint	• Change (Study Day 1 to Study Day 28) in patient/caregiver perceived cognitive deficit as reported using the PROMIS® Cognitive Function items<br>• Change (Study Day 1 to Study Day 28) in functional impairments as reported using the ADHD-Rating Scale 5 Home Version relationship with significant others, peer relationships, academic functioning, behavioral functioning, homework functioning and self-esteem domains<br>• Change (Study Day 1 to Study Day 28) in TOVA metrics other than ACS<br>• Responder analyses for TOVA-ACS and ADHD RS-5
Sample Size Justification	Sample sizes are calculated to test the null hypothesis of a zero-change from baseline in TOVA-ACS against the alternative of a positive change from baseline in TOVA-ACS with a one-sample t-test using EAST© Version 6.5. The significance level is defined by a one-sided α at 0.025, and a minimum power of 90% must be achieved. For the calculations it is assumed that the standard deviation σ in TOVA-ACS in this study population is as large as observed with TOVA-API in the study population of STARS-ADHD (SD=3.2). Assuming previously observed control-corrected effect size of μ=0.9 and observed standard deviation of σ=3.2, a sample size of 135 is required to detect the effect with 90% power.
Adjunct Study	Participants whose TOVA ACS score is greater than -1.8 but less than 0 and meet all other study inclusion criteria will be enrolled into an adjunct study that will run in parallel with the primary study. Adjunct

	study participants will undergo the same treatment regimen as the primary study participants, have the same duration of study participation, and have the same number of study visits. They will have the same study assessments as the primary study with two exceptions- they will not be administered the urine test and TOVA at the exit visit. The primary endpoint for the adjunct study is change (Study Day 1 to Study Day 28) in the ADHD-RS-5 Home Version inattention scale and total scale scores. Exploratory endpoints include change (Study Day 1 to Study Day 28) in patient/caregiver perceived cognitive deficit reported using the PROMIS® Cognitive Function items; functional impairments as reported using the ADHD-RS-5 Home Version relationship with significant others, peer relationships, academic functioning, behavioral functioning, homework functioning and self-esteem domains; and responder analyses for ADHD RS-5. The adjunct study will enroll up to 100 participants. It is estimated that 85 participants are required to detect an effect size of 0.5 with 90% power at the 95% confidence level using a within-group t-test.

Table of Contents

Protocol Approval Page 2

Statement of Compliance..... 3

Protocol Version and Amendment Tracking 4

Protocol Synopsis 5

Abbreviations..... 14

1. INTRODUCTION..... 15

1.1 Background 15

1.2 Clinical Experience with Study Agent 15

2. OBJECTIVES..... 16

3. STUDY POPULATION 17

3.1 Inclusion Criteria..... 17

3.2 Exclusion Criteria 17

3.3 Screen Failures & Adjunct Study..... 18

3.3.1 Screen Failures 18

3.3.2 Adjunct Study 18

4. STUDY DESIGN 18

4.1 Overview..... 18

4.2 Study Schedule of Procedures 19

4.3 Screening Visit 20

4.4 Baseline Procedures..... 21

4.5 At-Home Treatment Phase 21

4.6 Exit/Post-Treatment Visit..... 22

4.6.1 Adjunct Study Procedures..... 23

4.7 End of Study Definition 23

4.7.1 Study Completion..... 23

4.7.2 Participant Withdrawal or Discontinuation 23

4.7.3 Lost to Follow-Up 24

5. STUDY INTERVENTION..... 24

5.1 Study Interventions Description 24

5.2 Study Intervention Compliance 25

5.3 Potential Benefits/Risks of Study..... 26

5.4 Withdrawal/Discontinuation of Study Interventions 26

6. EFFICACY ASSESSMENTS 27
6.1 TOVA 27
6.2 ADHD Rating Scale-5 27
6.3 PROMIS® Cognition 27
6.4 Measures to Minimize Bias 28
7. CONCOMITANT MEDICATIONS AND THERAPIES 28
8. SAFETY 29
8.1 Definitions of Adverse Device (Software) Effect 29
8.1.1 Adverse Device (Software) Effect (ADE) 29
8.1.2 Unanticipated Adverse Device Effects (UADEs) 29
8.2 Previously Noted Adverse Device (Software) Effects 30
8.3 Collection of Adverse Device (Software) Effects 30
8.4 Assessment of Adverse Device (Software) Effect 31
8.4.1 Relationship to Study Intervention 31
8.4.2 Severity of Adverse Device (Software) Effects 32
8.4.3 Outcome of Unanticipated Adverse Device (Software) Effects 32
8.5 Reporting of Adverse Device (Software) Effects 32
8.5.1 Site Reporting 32
8.5.2 Institutional Review Board Reporting 33
8.5.3 Regulatory Reporting 33
9. DATA AND SAFETY MONITORING 33
9.1 Study Data Collection and Data Entry 33
9.2 Study Dataset 34
9.3 Endpoint Determination 34
9.4 Safety Monitoring 34
9.5 Clinical Monitoring and Quality Control (QC) 34
9.6 Future Use of Stored Data 35
10. STATISTICAL CONSIDERATIONS 35
10.1 Endpoints 36
10.3 Statistical Analysis 37
10.3.1 Analysis of the Primary Endpoint 37
10.3.2 Analysis of Secondary Endpoints 38
10.3.3 Analysis of Exploratory Endpoints 38
10.3.4 Subgroup Analyses 39
10.3.5 Adjunct Study 39
10.4 Sample Size 39
10.5 Safety Analysis 39

11. OPERATIONAL CONSIDERATIONS 40
11.1 Informed Consent 40
11.1.1 Consent/Assent Requirements 40
11.1.2 Consent/Assent Documentation 40
11.1.3 Other Information and Materials Provided to Participants 41
11.2 Confidentiality and Privacy 41
11.3 Trial Master File and Records Retention 41
11.4 Protocol Deviations 42
11.5 Study Discontinuation and Closure 42
11.6 Audits and Inspections 43
11.7 Conflict-of-Interest Policy 43
12. REFERENCES 44

Abbreviations

ACS	Attention Comparison Score (current/updated name of API)
ADHD	Attention Deficit Hyperactivity Disorder
ADHD Rating Scale-5	Attention Deficit Hyperactivity Disorder Rating Scale 5
API	Attention Performance Index (previous name of the ACS)
APP	Application
CFR	Code of Federal Regulations
CONSORT	Consolidated Standards of Reporting Trials
CRF	Case Report Form
DSM-5	Diagnostic and Statistical Manual of Mental Disorders, Fifth Edition
eCRF	Electronic Case Report Form
FDA	Food and Drug Administration
GCP	Good Clinical Practice
HIPAA	Health Insurance Portability and Accountability Act
ICF	Informed Consent Form
ICH	International Conference on Harmonisation
IDE	Investigational Device Exemption
IRB	Institutional Review Board
KBIT-II	Kaufmann Brief Intelligence Test, Second Edition
MAOI	Monoamine oxidase inhibitor
OTC	Over the counter
PI	Principal Investigator
PROMIS	Patient-Reported Outcomes Measurement Information System
QC	Quality Control
SAP	Statistical analysis plan
SSRI	Selective serotonin reuptake inhibitor
STARS-ADHD	Software Treatment for Actively Reducing Severity of ADHD as Adjunctive Treatment to Stimulant
TE-ADE	Treatment-Emergent Adverse Device Effect
TOVA	Test of Variables of Attention
UADE	Unanticipated Adverse Device Effect
UP	Unanticipated problems

1. INTRODUCTION

1.1 Background

First-line treatment for attention deficit hyperactivity disorder (ADHD) includes the use of stimulant medications, which is effective in reducing core symptoms of the disorder but also associated with well-documented side effects. In addition, there is recent evidence that pharmacological treatment may not show optimal benefits in some cognitive domains.[1] Computerized cognitive training programs have shown some promise in improving working memory and attention in ADHD populations.[2] Novel, cost-effective, non-pharmacological interventions for ADHD which are easy to implement and disseminate could be helpful for many participants given the limitations of other approved interventions.

AKL-T01 is a novel, highly immersive, digital therapeutic intervention which is currently FDA cleared as EndeavorRx™. EndeavorRx™ is a digital therapeutic indicated to improve attention function as measured by computer-based testing in children ages 8-12 years old with primarily inattentive or combined-type ADHD, who have a demonstrated attention issue. Patients who engage with EndeavorRx™ demonstrate improvements in a digitally assessed measure, Test of Variables of Attention (TOVA®), of sustained and selective attention and may not display benefits in typical behavioral symptoms, such as hyperactivity. EndeavorRx™ should be considered for use as part of a therapeutic program that may include clinician-directed therapy, medication, and/or educational programs, which further address symptoms of the disorder.

AKL-T01 is deployed on mobile devices (i.e., tablets) and incorporates adaptive, simultaneous cognitive tasks in a consumer-grade action videogame-based platform with high-quality graphics and reward mechanisms.

This protocol will extend existing data supporting AKL-T01's effects on objective attention functioning and ADHD symptoms/impairment, to children aged 13-17 years old.

1.2 Clinical Experience with Study Agent

AKL-T01 was studied in the Software Treatment for Actively Reducing Severity of ADHD as Adjunctive Treatment to Stimulant (STARS-ADHD) study; a multi-center, randomized, double-blind, active-controlled study comparing AKL-T01 treatment to the use of a digital control in 348 pediatric participants aged 8-12 years, diagnosed with ADHD and a measurable impairment in objective attention function (as indicated by the Test of Variables of Attention (TOVA®), Attention Performance Index (API) ≤ -1.8) who were not taking ADHD medication. 180 participants were randomized into AKL-T01, and 168 into

the control group and both groups were instructed to engage with their intervention for approximately 25 minutes/day, 5days/week for 4 weeks. The study successfully demonstrated a statistically significant improvement (p=0.006) between AKL-T01 and control on the primary endpoint (TOVA-API [a composite measure of objective attention] change from Baseline to after 4-weeks of treatment).[3]

A subsequent open-label study STARS-Adjunct included children ages 8-14 years old, both on and off stimulant ADHD medication, and found comparable effects in these two cohorts on ADHD-related impairment and ADHD symptoms. Children with measurable attention impairment at baseline comparable to STARS-ADHD TOVA Attention Comparison Score (ACS ≤-1.8) showed improvement in objective attention function. In both these studies, AKL-T01 demonstrated a favorable safety profile, with no serious adverse events observed.

Two separate studies of 20 and 19 children with ADHD and Sensory Processing Disorder and Autism Spectrum Disorder, where AKL-T01 was used for a 4-week treatment period and showed improvements in attention measures and attention-related ADHD symptoms.[4,5]

2. OBJECTIVES

- The primary objective of this study is to evaluate the efficacy of AKL-T01 as determined by the change in a digitally assessed measure, Test of Variables of Attention (TOVA®)- Attention Comparison Score (ACS), of sustained and selective attention, after 4 weeks of treatment
- A secondary objective of this study is to evaluate the change in ADHD symptoms, as determined by change in the ADHD Rating Scale-5 inattention sub-scale and total scale scores, after 4 weeks of treatment with AKL-T01
- Exploratory objectives of this study are
    a. To evaluate the change in patient and caregiver perceived cognitive deficits as determined by the change in selected items from the PROMIS® Parent Proxy Item Bank v1.1 – Cognitive Function and the PROMIS® Pediatric Item Bank v1.0-Cognitive Function after 4 weeks of treatment with AKL-T01
    b. To evaluate changes in functional impairment as determined by change in ADHD-Rating Scale 5 Home Version relationship with significant others, peer relationships, academic functioning, behavioral functioning, homework functioning and self-esteem domains
    c. To evaluate changes in TOVA metrics other than ACS after 4 weeks of AKL-T01 treatment
    d. To evaluate response rates for TOVA-ACS and ADHD RS-5 after 4 weeks of AKL-T01 treatment

3. STUDY POPULATION

3.1 Inclusion Criteria

To be eligible for this trial, participants must meet all the following criteria:

1) Adolescents between the ages of 13 and 17 years and 10 months at time of consent (must be under 18 years at study completion)
2) Confirmed diagnosis of ADHD combined or inattentive type, according to Diagnostic and Statistical Manual of Mental Disorders, Fifth Edition (DSM-5) as confirmed by MINI-Kid Version 7.0.2.
3) Stably on or off ADHD medication for ≥4 weeks prior to study enrollment and throughout the 4-week study
4) Baseline visit score on the TOVA-ACS score ≤ -1.8
5) Access to and self-report of ability to connect wireless devices to a functional wireless network
6) Ability to follow written and verbal instructions (English) as assessed by the PI and/or study coordinator
7) Able to comply with all testing and study requirements
8) Estimated IQ score ≥80 as assessed by the Kaufmann Brief Intelligence Test, Second Edition (KBIT-II)
9) Patient assent and caregiver informed consent
10) Stably on or off psychoactive medications for ≥4 weeks prior to study enrollment and throughout the 4-week study

3.2 Exclusion Criteria

If a participant meets any of the following criteria, he or she may not be enrolled in the study:

1) Current controlled or uncontrolled, comorbid psychiatric diagnosis that in the opinion of the Investigator may confound study data/assessments.
2) Participant is currently considered at risk for attempting suicide, has made a suicide attempt within the past year, or is currently demonstrating active suicidal ideation or self-injurious behavior in the opinion of the Investigator based on the MINI-kid clinical interview.
3) Motor condition (e.g., physical deformity of the hands/arms) that prevents game playing as reported by the participant or observed by the Investigator.
4) Recent history (6 months prior to screening) of substance use disorder
5) History of seizures (excluding febrile seizures), significant tics, or a current diagnosis of Tourette's Disorder.
6) Known sensitivity to playing video games, such as photo-sensitive epilepsy, light-headedness, dizziness, nausea, or motion sickness.
7) Participation in a clinical trial within 3 months prior to screening.

8) Plans to initiate, or to make significant changes in frequency, of non-pharmacological behavioral therapy during the study
9) Color blindness as detected by Ishihara Color Blindness Test
10) Urine test positive for nicotine or marijuana
11) Any other medical condition that in the opinion of the Investigator may confound study data/assessments.
12) Previous exposure to Akili Products within the 6 months prior to study enrollment
13) Plans to initiate or to make significant changes in frequency or duration of non-pharmacological trainings with the aim to improve cognition by means of game or app-based cognitive trainings or neurofeedback, during the study.

3.3 Screen Failures & Adjunct Study

3.3.1 Screen Failures

Screen failures are defined as participants who consent to participate in the clinical trial but are not subsequently enrolled into the primary study. A minimal set of information is required to ensure transparent reporting of participant screen failures, to meet the Consolidated Standards of Reporting Trials (CONSORT) publishing requirements and to respond to queries from regulatory authorities. Minimal information includes demography, screen failure details, and eligibility criteria.

3.3.2 Adjunct Study

Participants whose TOVA ACS score is greater than -1.8 but less than 0 and meet all other study inclusion criteria will be enrolled into an adjunct study that will run in parallel with the primary study. Adjunct study participants will undergo the same treatment regimen as the primary study participants, have the same duration of study participation, and have the same number of study visits.

4. STUDY DESIGN

4.1 Overview

This study is a multi-center, unblinded/non-controlled study to evaluate objective attention functioning and ADHD symptoms and impairments in children between adolescents aged 13 to 17 years old, with a diagnosis of ADHD (combined or inattentive subtype), stably on or off ADHD medication, after 4-weeks of AKL-T01 treatment.

Up to 165 total participants from up to 20 sites will be enrolled. Enrollment is competitive and there are no caps per site.

Study participation is expected to be 32 days: Baseline visit, 28-day treatment phase, and an Exit Visit on day 28 (+3) days.

Participants will have on-site visits for baseline and exit/post-treatment study assessments. Participants will complete a 28 day at-home treatment phase in between the baseline and exit visit.

4.2 Study Schedule of Procedures

Procedure	Screening Visit	Baseline Procedures Day 1 [1]	At-Home Treatment Phase Days 2-27	Exit / Post-treatment Visit Day 28 (+3)
**Caregiver Informed Consent**	X			
**Participant Assent**	X			
**Demographics**	X			
**MINI-KID v7.0.2 Section N [3]**	X			
**MINI-KID v7.0.2 Section B**	X			
**Urine test for nicotine and marijuana [2][7]**	X			X
**TOVA (Version 9) [2][7]**	X			X
**Kaufmann Brief Intelligence Test [2]**	X			
**Ishihara Color Blindness Test [2]**	X			
**Medical History**		X		
**ADHD History**		X		
**MINI-KID v7.0.2 Remaining Sections [3]**		X		
**ADHD Rating Scale-5 Home Version**		X		X
**PROMIS Cognition parent proxy and pediatric**		X		X
**Assign iPad-Mini**		X		
**Intervention Training**		X		
**AKL-T01 Treatment [4]**			←——————→	
**AKL-T01 Verify Data Upload**		X		
**AKL-T01 compliance check [5]**			←——————→	
**Collect iPad-Mini and charger**				X

**Participant Experience Survey**				X
**Prior and Concomitant Medications/Therapies [6]**		X	←——————→	
**Treatment Emergent Adverse Device Effect**		←——————→		
	[1] Baseline visit may be conducted on a different day from screening visit based on site discretion<br>[2] Assessments may be conducted on a different day from screening visit based on site discretion<br>[3] Unless the assessment was completed within the 3 months prior to screening except for MINI-KID Section B (suicidality) which must be completed on the day of the screening visit<br>[4] Approximately 25 minutes/day for at least 5 days/week of AKL-T01 across the 4-week (28 day) treatment period<br>[5] Compliance check as needed throughout 28-day treatment<br>[6] Capture changes to medications and dosage, include non-pharmacological therapies, as reported<br>[7] Adjunct participants will complete the same schedule of activities as primary study participants with the exception of urine test and TOVA being omitted at the exit visit.			

4.3 Screening Visit

For this on-site visit, participants should be instructed to avoid caffeine (e.g., coffee, tea, sodas, energy drinks, caffeine pills, etc.) for 4-6 hours prior to the study visit. At the investigator's discretion, participants may also be instructed to delay their ADHD medication, for that day, to after completion of the TOVA assessment. The following study procedures and assessments will be performed during the screening visit:

- Informed consent will be obtained from the participant's caregiver (parent or legal guardian) and written assent will be obtained from the participant
- Preferred method of contact will be collected
- Demographic information will be collected
- MINI-KID v7.0.2 Section N to establish ADHD diagnosis. If a MINI-KID assessment was performed within the 3 months prior to the screening visit, those results can be entered into the EDC and it is not necessary to repeat the assessment.
- Assess suicidality using MINI-KID v7.0.2 Section B regardless of when the last MINI-KID assessment was performed
- A urine test to detect nicotine and marijuana
- Test of Variables of Attention (TOVA)
    - All participants should have a minimum 10-minute break from previous study procedure before TOVA administration
- KBIT-II
- Ishihara Color Blindness Test

4.4 Baseline Procedures

If a participant meets all eligibility criteria, the following study procedures and assessment will be performed:

- Medical history and ADHD history will be collected.
- Review of prior and concomitant medications (including dosages), behavioral therapies or cognitive and related training, including EEG neuro feedback training
- Remaining sections of MINI-KID v7.0.2 If a MINI-KID assessment was performed within the 3 months prior to the screening visit, those results can be entered into the EDC and it is not necessary to repeat the assessment. This applies to all sections of the MINI-KID except the suicidality section B.
- ADHD-Rating Scale 5 Home Version (Symptom scales and Functional Impairment Domains)
- Selected items from the PROMIS® Parent Proxy Item Bank v1.1- Cognitive Function and Pediatric Item Bank v1.0-Cognitive Function
- An iPad Mini, loaded with AKL-T01 will be assigned and provided to the participant. Intervention Training will be given to the participant delivering proper device and software usage.
- The participant will initiate their first treatment day at the clinic. At a minimum, the participant must complete the tutorial and analysis missions in-clinic.
- Site staff will verify data upload on the Akili Dashboard to confirm the treatment registered.
- Caregivers will be informed to notify the site during the At-Home Treatment portion if there are any changes to the participants Concomitant Medications or if any Treatment Emergent Adverse Device Effects (TE-ADEs) occur.

The following materials are provided to the parent to take home:

- Signed informed consent
- The iPad they were assigned
- Charger
- Intended use instructions: Set up of the participants' local Wi-Fi network for internet access to push play and compliance data to Akili secure servers, and completion of at-home intervention for the assigned 25 minutes per day, during at least 5 days of each calendar week.

4.5 At-Home Treatment Phase

Participants will initiate at-home play with the AKL-T01 treatment. Participants will be asked to complete AKL-T01 treatment per the Study Application Usage instructions given during the Intervention Training.

To access the AKL-T01 intervention, participants will log into their assigned device with a unique user ID and password. Details about performance and duration of engagement will be automatically recorded and uploaded to Akili central servers when the device is connected to Wi-Fi. Compliance with requirements will be monitored automatically.

Compliance checks will be performed throughout the treatment phase. Automatic compliance emails will be sent to study staff daily. Study staff will contact active participants who do not complete the required treatment after 48 hours of consecutive non-compliance. The software will also automatically alert participants when they have not interacted with the application for more than 24 hours.

Caregivers are to notify the site during the At-Home Treatment portion if there are any changes to the participant's concomitant medications or if any Treatment Emergent Adverse Device Effects (TE-ADEs) occur. A Caregiver Card will be provided to Caregivers, which contains spaces for the caregiver to record ADE start/stop dates, concomitant medication changes, and the date of the scheduled exit/post-treatment visit. The Caregiver Card will be submitted to the site at the Exit/Post-treatment visit. Site will transcribe any changes that may have occurred during the treatment portion of the study into the EDC.

4.6 Exit/Post-Treatment Visit

The exit visit should be scheduled at approximately the same time of day (i.e., morning or afternoon) as the Baseline visit. The participant must be accompanied by the same parent/caregiver as for the Baseline visit. As with the screening visit, participants will be instructed to avoid caffeine for 4-6 hours prior to the visit. At the investigator's discretion, the participant may also be asked to delay their ADHD medication, for that day, to after completion of the TOVA assessment.

Participants will arrive at the study clinic with the assigned iPad-Mini and charger and return the device to the site. The caregiver will submit their Caregiver Card.

The same study clinician who performed the baseline visits must perform all study assessments for the Exit Visit.

- A urine test to detect nicotine and marijuana
- Test of Variables of Attention (TOVA)
- ADHD-Rating Scale 5 Home Version (Symptom scales and Functional Impairment Domains)
- Selected items from the PROMIS® Parent Proxy Item Bank v1.1- Cognitive Function and Pediatric Item Bank v1.0-Cognitive Function
- Review of concomitant medications and therapies
- Participant Experience Survey will be completed by caregivers and participants, to capture general game-play experience and satisfaction with treatment.

4.6.1 Adjunct Study Procedures

Study procedures in the adjunct study are identical to the primary study for the screening and baseline visits and at-home treatment phase. Adjunct study participants will undergo the same procedures as the primary study participants during the exit visit with the exception of the urine test and TOVA which should not be administered.

4.7 End of Study Definition

4.7.1 Study Completion

A participant is considered to have completed the study if he or she has completed all phases of the study including the Exit Visit, at Day 28 (+3).

4.7.2 Participant Withdrawal or Discontinuation

Participant Withdrawal

Participants are free to withdraw from participation in the study at any time upon request, for any reason without prejudice.

Investigator Discontinuation of Participant

Participants may be discontinued from the study at the discretion of the investigator if medically necessary or if any untoward effects occur. In addition, a participant may be discontinued by the investigator (in consultation with the sponsor as necessary) for the following reasons:

- Significant study treatment non-compliance (defined as <30 missions played) with prescribed study treatment observed in the first two weeks following enrollment
- If any clinical adverse device effect (ADE), or other medical condition or situation occurs such that continued participation in the study would not be in the best interest of the participant
- Disease progression worsening, to the extent that it, requires discontinuation of the study treatment
- If the participant meets an exclusion criterion (either newly developed or not previously recognized) that precludes further study participation
- Or otherwise violates the study plan, or for administrative and/or other safety reasons

The reason for participant withdrawal/discontinuation from the study will be recorded on the study Case Report Form (CRF).

Participants who sign the informed consent form but do not receive the study treatment will be counted as screen failures. Participants who sign the informed consent form and

receive the study treatment will be counted as enrolled participants, even if they subsequently withdraw, or are discontinued from the study.

The investigator or designee will notify the sponsor or their designee immediately when a participant has withdrawn or been discontinued from the study because of an adverse event. When a participant withdraws or is discontinued from the study before study completion, all applicable activities scheduled for the final study visit should be performed at the time of discontinuation. Any adverse events that are present at the time of withdrawal/discontinuation should be reported and followed up in accordance with the safety requirements outlined in the Adverse Device (Software) Effect Reporting and Follow-Up section.

Participants may withdraw by contacting the investigator or study staff at their site.

4.7.3 Lost to Follow-Up

A participant will be considered lost to follow-up if they fail to return for the final study visit and are unable to be contacted by the study site staff.

The following actions must be taken if a participant fails to return to the clinic for a required study visit:

- The site will attempt to contact the participant and reschedule the missed visit within a one-week window of the missed visit and counsel the participant on the importance of maintaining the assigned visit schedule and ascertain if the participant wishes to and/or should continue in the study.
- Before a participant is deemed lost to follow-up, the investigator or designee will make every effort to regain contact with the participant (where possible, 3 telephone calls and, if necessary, a certified letter to the participant's last known mailing address or local equivalent methods). These contact attempts should be documented in the participant's study file.
- Should the participant continue to be unreachable, they will be considered to have withdrawn from the study with a primary reason of loss to follow-up.

5. STUDY INTERVENTION

5.1 Study Interventions Description

AKL-T01 is a digital therapeutic built using Akili's proprietary algorithm (Selective Stimulus Management Engine (SSME™)) designed to train interference management at an adaptive and personalized high degree of difficulty. Interference is instantiated through a video game-based interface displaying two tasks that are to be done in parallel (multitasking); a perceptual discrimination targeting task in which users respond

to the instructed stimulus targets and ignore the stimulus distractors (similar to a Go–No-Go task), and a sensory motor navigation task in which users continuously adjust their location to interact with or avoid positional targets. Performance in each task is assessed during single and multitask conditions. As users proceed through the treatment, periodic recalibration occurs to maintain an optimal difficulty level.

Participants should allow approximately 25 minutes of time to complete each daily treatment, which consists of playing between 6-8 missions per day. Participants will be instructed to play at least 5 days/week during each of the 4 weeks of the treatment phase of the study. Participants may initiate play immediately after receiving their study device. Specifics about which days and times can be used for play can be chosen by each participant based on their schedules and may vary among participants as long as the above play requirements are met. Actual time of play will be captured by the software.

The device should be connected to the participant's home Wi-Fi network or cellular service. If home Wi-Fi or cellular service is not available, the parents and participants need to establish a plan where the device will be connected to remote Wi-Fi on a periodic basis during the treatment. To initiate play, the participant taps on the game icon displayed on the screen and follows the in-program instructions. Separate electronic instructions for use (IFUs) will also be accessible on the study device.

For any calendar day, the treatment will automatically lock after the participants complete their allocated maximum number of daily missions and no further play will be allowed until the next calendar day. Access to the treatment will be disabled at the end of the study.

5.2 Study Intervention Compliance

AKL-T01 automatically captures gameplay compliance and uploads these data directly to a central server when connected to Wi-Fi. The server will automatically push daily compliance emails to the clinical sites. Based on these emails, study staff can then reach out to caregivers (via the preferred contact method captured in the CRF) to troubleshoot technical problems and/or encourage more play. The rule for compliance outreach is as follows:

Participants that fail to play any missions of AKL-T01 over two consecutive days will be contacted to troubleshoot potential Wi-Fi connection problems.

Participants that fail to record at least 15 missions of AKL-T01 over 7 consecutive days will be contacted a second time and reminded to continue their study intervention.

Additionally, AKL-T01 contains built-in features that remind the participant to play each day.

5.3 Potential Benefits/Risks of Study

The commercial version of AKL-T01, EndeavorRx®, is a digital therapeutic indicated to improve attention function as measured by computer-based testing in children ages 8-12 years old with primarily inattentive or combined-type ADHD, who have a demonstrated attention issue. Patients who engage with EndeavorRx demonstrate improvements in a digitally assessed measure, Test of Variables of Attention (TOVA®), of sustained and selective attention and may not display benefits in typical behavioral symptoms, such as hyperactivity. EndeavorRx should be considered for use as part of a therapeutic program that may include clinician-directed therapy, medication, and/or educational programs, which further address symptoms of the disorder. EndeavorRx is not intended to be used as a stand-alone therapeutic and is not a substitution for medication.

Of 538 participants using EndeavorRx (AKL-T01), 50 participants (9.3%) experienced treatment-related adverse events (probable, likely), and three participants experienced treatment-related adverse events with the digital control, in studies where a control was used. EndeavorRx associated adverse events included frustration (6.1%), headache (1.3%), dizziness (0.6%), emotional reaction (0.4%), nausea (0.4%), and aggression (0.2%). All adverse events were generally transient. Only 3 events led to device discontinuation, and no subject reported lasting or irreversible effects after discontinuation. To date, there have been no serious adverse events reported across several studies with the device.

If a participant experiences frustration, emotional reaction, dizziness, nausea, headache, eyestrain, or joint pain while playing they should pause the treatment and if the problem persists, they should contact their healthcare provider. If a participant experiences a seizure, they should stop the treatment and contact their healthcare provider.

5.4 Withdrawal/Discontinuation of Study Interventions

Participants are free to withdraw from study intervention at any time upon request, for any reason.

An investigator may discontinue a participant from the study intervention for the same reasons as detailed in the Participant Discontinuation/Withdrawal from the Study section.

If a subject withdraws or is discontinued from study intervention, they will be subsequently withdrawn/discontinued from the study as described in the Participant Discontinuation/Withdrawal from the Study section.

6. EFFICACY ASSESSMENTS

6.1 TOVA

The TOVA® is an FDA-cleared, continuous performance test measuring attention and inhibitory control used in the evaluation of ADHD treatments.[6] The test provides several objective metrics related to attention function and generates an Attention Comparison Score (ACS). Age and gender-matched norms are available for children and adults 4 years and older.
Respondent: Child
Estimated time to complete: 22 minutes

6.2 ADHD Rating Scale-5

The ADHD Rating Scale-5 Home Version is a rating scale based on the diagnostic criteria for ADHD as described in the fifth edition of the Diagnostic and Statistical Manual of Mental Disorders (DSM-5).[7] The Home version consists of two symptom subscales, Inattention (9 items) and Hyperactivity-Impulsivity (9 items), as well as a Total Scale (18 items). In addition, the ADHD Rating Scale-5 assesses six domains of impairment that are common among children with ADHD: relationships with significant others, peer relationships, academic functioning, behavioral functioning, homework performance, and self-esteem. The ADHD Rating Scale-5 has a separate form for adolescents (ages 11-17 years) that incorporates age-appropriate symptom descriptions as per DSM-5.

6.3 PROMIS® Cognition

The PROMIS® Cognitive Function and Cognitive Function Abilities Subset item banks assess patient-perceived cognitive deficits.[8] Facets include mental acuity, concentration, verbal and nonverbal memory, verbal fluency, and perceived changes in these cognitive functions. The cognitive function instruments are universal rather than disease specific. All assess cognitive function over 4 weeks. Cognitive Function instruments are available for adults (ages 18+), pediatric self-report (ages 8-17) and for parents serving as proxy reporters for their child (youth ages 8-17).

For the Caregiver assessments, ten items from the Parent Proxy Item Bank v1.1-Cognitive Function will be administered in this study. The respondent is the caregiver (parent proxy) and estimated time to complete is 10-15 minutes.

For the Participant assessments, ten items from the Pediatric Item Bank v1.0-Cognitive Function) will be administered in this study. The respondent is the participant and estimated time to complete is 10-15 minutes.

6.4 Measures to Minimize Bias

Bias opportunities should be minimized by the removal of rater variability. Every effort should be made, throughout the course of the trial, to maintain the consistency of rating staff among participants.

To minimize the unintentional bias of study data and to maintain data consistency, clinical rater assessments for each participant should be completed at the same time of day throughout the course of the study. The TOVA assessment should also be completed at the same time of the day and a break should be allowed before the TOVA.

All attempts should be made to ensure that the same parent/caregiver completes the patient reported outcome measures.

Every effort must be made to ensure that raters of all rating scales/assessments are kept consistent among participants throughout trial participation. Each rating scale/assessment for the participant should be administered by the same rater for the duration of the trial. If a rater leaves a clinical site for any reason the site should replace the rater as quickly as possible, and this replacement rater will also be expected to complete rater training (if not previously trained). The replacement rater should review the participant's source documents for previously completed rating assessments, in an effort to become familiar with the former rater's evaluations.

7. CONCOMITANT MEDICATIONS AND THERAPIES

ADHD medications administered prior to treatment and until the final study visit will be recorded in the CRFs.

Participants may be enrolled into the study currently on stimulant or other types of ADHD medication, provided that the dosage has been stable for ≥ 4 weeks prior to the Baseline Visit. Participant's ADHD medication dosage should remain stable as possible during the course of the trial. Any titrations (including switching medications) of the participant's ADHD medication during the course of their participation in the trial will be documented within the CRFs. On the screening and exit study visit days, at the investigator's discretion, participants may delay their dose of ADHD medication to until after they have completed the TOVA assessment.

Participants may be enrolled into the study on psychoactive medications (e.g. SSRIs, MAOIs, tricyclic antidepressants, antipsychotics etc.), provided that the participants' medication and dosage has been stable for ≥ 4 weeks prior to the Baseline Visit. Participant's medication dosage should remain as stable as possible during the course of

the trial. Any titrations (including switching medications) of the participant's medication during the course of their participation in the trial will be documented within the CRFs.

Initiation of or significant changes in non-pharmacological behavioral therapy, or non-pharmacologic trainings with game or app-based cognitive training or neurofeedback, (including but not limited to Cogmed or Attentive) for ADHD, is not permitted during the study. Participants actively engaged in non-pharmacological treatment at screening may be eligible if they meet all inclusion criteria and do not undergo changes to their treatment during the study. The participant should inform the Investigator if they intend to change their non-pharmacological therapy during the 4 weeks of the study.

8. SAFETY

Safety assessments will include the evaluation of adverse device effects (ADEs). Other Assessments will include unanticipated problems (UP).

8.1 Definitions of Adverse Device (Software) Effect

8.1.1 Adverse Device (Software) Effect (ADE)

Defined as an adverse event related to the use of an investigational medical treatment delivered through software application. This includes any adverse event resulting from insufficiencies or inadequacies in the instructions for use, the deployment, the installation, the operation, or any malfunction of the investigational medical software. This also includes any event that is a result of a use error or intentional misuse.

Treatment Emergent ADEs (TE-ADE) indicate ADEs to be collected at the point of exposure to the treatment and time thereafter.

8.1.2 Unanticipated Adverse Device Effects (UADEs)

A Unanticipated Adverse Device Effect is any serious adverse effect on health or safety or any life-threatening problem or death caused by, or associated with, a device, if that effect, problem, or death was not previously identified in nature, severity, or degree of incidence in the investigational plan or application (including a supplementary plan or application), or any other unanticipated serious problem associated with a device that relates to the rights, safety, or welfare of participants.

Unanticipated Adverse Device Effects will include events meeting A and B as stated below:

A. Events meeting **ALL** the following criteria:
   - Not included in the list of Anticipated Events (previously noted ADEs)
   - Possibly, probably, or definitely related to the investigational device per the PI

B. Serious (meets **ANY** of the following criteria):
   - Is a life-threatening illness or injury
   - Requires inpatient or prolonged hospitalization
   - Results in permanent (i.e., irreversible impairment or damage to a body structure or function, excluding trivial impairment or damage) impairment of a body structure
   - Necessitates medical or surgical intervention to prevent permanent impairment of a body function or a body structure
   - Led to fetal distress, fetal death or a congenital abnormality or birth defect
   - Led to death

8.2 Previously Noted Adverse Device (Software) Effects

Adverse Device (Software) Effects expected for **AKL-T01**:

1) Dizziness
2) Nausea
3) Headache
4) Decreased frustration tolerance
5) Emotional reaction
6) Aggression

The expectedness of an ADE shall be documented in the informed consent form. Any ADE that is not identified in nature, severity, or is not listed above is considered unanticipated.

8.3 Collection of Adverse Device (Software) Effects

A TE-ADE observed from the time of Intervention Training at the Baseline visit through the Exit visit will be collected in the study database.

At each study visit, the investigator will inquire about the occurrence of TE-ADEs since the last visit. TE-ADEs may be reported via participant or caregiver communication with the study team during the at-home treatment phase or interviews of a study participant presenting for medical care. Study monitors may identify a TE-ADE upon review of subject source documentation.

All TE-ADEs including local and systemic reactions will be captured on the appropriate case report form (CRF). Information to be collected includes the event description, time of onset, relationship to study product (assessed only by those with the training and authority to make a diagnosis), clinician's assessment of severity, and time of resolution/stabilization of the event.

All TE-ADEs occurring while in the study will be documented appropriately regardless of relationship.

Any medical condition that is present at the time that the participant is screened will be considered as baseline and not reported as an ADE. However, if the study participant's condition deteriorates at any time during the study, it will be recorded as a TE-ADE.

Changes in the severity of a TE-ADE will be documented and presented by maximum severity. TE-ADEs characterized as intermittent require documentation of onset for each episode.

Events will be followed for outcome information until resolution or stabilization or until the final study visit.

8.4 Assessment of Adverse Device (Software) Effect

8.4.1 Relationship to Study Intervention

All TE-ADEs must have their relationship to study treatment assessed by the clinician who examines and evaluates the participant based on temporal relationship and his/her clinical judgment. In a clinical trial, the investigational device must always be suspect. The degree of certainty of causal relationship of an adverse device effect of either study treatment will be rated as follows:

- **Possible:** An event that might be due to the use of the study application. An alternative explanation - e.g., concomitant drug(s), concomitant disease(s) - is inconclusive. The relationship in time is reasonable; therefore, the causal relationship cannot be excluded.
- **Probable:** An event that might be due to the use of the study application. An alternative explanation is less likely - e.g., concomitant drug(s), concomitant disease(s). The relationship in time is suggestive.
- **Definite:** An event that is due to the use of the study application. The event cannot be reasonably explained by an alternative explanation - e.g., concomitant drug(s), concomitant disease(s).

8.4.2 Severity of Adverse Device (Software) Effects

The severity of a TE-ADE will be rated as follows:

- **Mild:** Awareness of sign, symptom, or event, but easily tolerated.
- **Moderate:** Discomfort enough to cause interference with usual activity and may warrant intervention.
- **Severe:** Incapacitating with inability to do usual activities or significantly affects clinical status and warrants intervention.

8.4.3 Outcome of Unanticipated Adverse Device (Software) Effects

If the Sponsor determines that an UADE presents an unreasonable risk to participants, all investigations or parts of investigations presenting that risk shall be terminated as soon as possible. Termination shall occur no later than 5 working days after the Sponsor makes this determination and no later than 15 working days after the first received notice of the effect.

8.5 Reporting of Adverse Device (Software) Effects

It is understood that complete information about an event may not be known at the time the initial report is submitted. The investigator must assess the relationship of the event to the investigational device/software (including rationale for assessment) and should make every attempt to obtain as much information as possible concerning the event.

Additional information pertaining to an event should be reported in the clinical database as it becomes available.

8.5.1 Site Reporting

Within 24 hours of knowledge of the event, the investigator must report all related TE-ADEs, anticipated or unanticipated, occurring from the time of study Intervention Training, Baseline Day 1, in the clinical database.

The investigator must document when important follow-up information (final diagnosis, outcome, results of specific investigations, etc.) becomes available after documentation of the initial Suspected Adverse Reaction information.

Follow-up information should be collected according to the same process used for documenting the initial event as described above.

8.5.2 Institutional Review Board Reporting

The investigator will submit all related unanticipated, and serious TE-ADEs, to the reviewing Institutional Review Board (IRB) according to IRB policies and procedure.

8.5.3 Regulatory Reporting

A sponsor who conducts an evaluation of an UADE under 21 CFR 812.46(b) shall report the results of such evaluation to the Food and Drug Administration (FDA) and to all reviewing IRBs and participating investigators within 10 business days after the sponsor first receives notice of the effect.

Thereafter, the sponsor shall submit such additional reports concerning the effect as FDA requests.

9. DATA AND SAFETY MONITORING

9.1 Study Data Collection and Data Entry

Clinical data is any data point that is captured outside of the AKL-T01 gameplay data.

Clinical data for this study will be captured in an electronic format. Each participant will be assigned a series of electronic Case Report Forms (eCRFs) that will be contained in the Electronic Data Capture (EDC) system. Details of oversight and management of the eCRFs/EDC will be detailed in the study Data Management Plan.

The investigator must ensure that all staff involved in the conduct of the trial are familiar with the protocol and all study-specific procedures, and that they have appropriate knowledge of the study agents. The investigator, or personnel delegated by the investigator, will perform primary data collection based on the protocol design and assessments performed. Site staff will perform data entry into the EDC based on the data captured in the original source documentation.

This study will use a study specific electronic case report form (eCRF) to capture all clinical data. All required study information must be recorded on the appropriate eCRF screen/form using the eCRF Completion Guidelines for the study. A CRF must be completed for each participant.

All data must be carefully entered in a timely fashion to permit meaningful interpretation and study oversight. Required clinical data will be entered in the eCRF as soon as possible after collection, and no later than 3 business days after the completed visit.

The investigator is responsible for ensuring that the clinical data is entered into the eCRF appropriately. The investigator is responsible for signing off on the final eCRF prior to locking.

AKL-T01 gameplay data will be collected on the iPad and automatically transferred to Akili upon connection of the iPad to the internet.

9.2 Study Dataset

The full study dataset will be collected for participants who enter the study by receiving study Intervention Training (AKL-T01).

Limited data (i.e., demographics, adverse events, selection criteria, and reason for discontinuation) will be collected for participants who screen fail or discontinue before the treatment phase.

All required data for this study will be entered or uploaded into the eCRF.

9.3 Endpoint Determination

Data from the TOVA assessment will be derived directly from the computers used to administer the task and these data will be shared with Akili for analysis of primary outcome. Data from all other assessments will be collected by study personnel.

9.4 Safety Monitoring

Safety/tolerability will be assessed primarily at the Post-Treatment Visit (Visit 3), as well as during the 4-week game play component as necessary.

9.5 Clinical Monitoring and Quality Control (QC)

Each clinical site will perform internal quality management of study conduct and data collection, documentation, and completion per site standard operating procedures.

The investigational site will provide direct access to all trial-related source data/documents, and reports for the purpose of monitoring and auditing by the sponsor and inspection by local and regulatory authorities.

A study Clinical Management Plan will be developed to include details regarding QC and monitoring of the clinical data. Oversight and management of the clinical data will be detailed in the study Data Management Plan.

Source data verification procedures will be implemented beginning with the data entry system, and data QC checks on the database will be generated. Sites will be queried on any missing data or data anomalies for clarification/resolution.

The study monitor will conduct risk-based source data verification (SDV) as detailed in the Clinical Monitoring Plan to ensure maximum data integrity.

9.6 Future Use of Stored Data

Data collected for this study will be analyzed and stored at Akili. After the study is completed, the coded, archived data will be transmitted to and stored at Akili and may be used for additional post hoc analyses.

10. STATISTICAL CONSIDERATIONS

This section provides a brief overview of the statistical analyses, both quantitative and qualitative in nature, that will be used during this study. In addition to what follows in this section, further details will be provided in a separate statistical analysis plan (SAP).

- The primary objective of this study is to evaluate the efficacy of AKL-T01 as determined by the change in a digitally assessed measure, Test of Variables of Attention (TOVA®)- Attention Comparison Score (ACS), of sustained and selective attention, after 4 weeks of treatment
- A secondary objective of this study is to evaluate the change in ADHD symptoms, as determined by change in the ADHD Rating Scale-5 inattention sub-scale and total scale scores, after 4 weeks of treatment with AKL-T01
- Exploratory objective of this study include:
    a. To evaluate the change in patient perceived cognitive deficits as determined by the change in selected items from the PROMIS®– Cognitive Function Item Bank (Parent Proxy Item Bank v1.1-Cognitive Function and Pediatric Item Bank 1.0 Item Bank-Cognitive Function) after 4 weeks of treatment with AKL-T01

b. To evaluate change in functional impairments as reported using the ADHD-Rating Scale 5 Home Version relationship with significant others, peer relationships, academic functioning, behavioral functioning, homework functioning and self-esteem domains
c. To evaluate changes in TOVA metrics other than ACS after 4 weeks of treatment with AKL-T01
d. To evaluate response rates for TOVA-ACS and ADHD RS-5 after 4 weeks of treatment with AKL-T01

10.1 Endpoints

- The primary endpoint of this study is change (Study Day 1 to Study Day 28) on the TOVA-ACS.
- The secondary endpoints of this study are change (Study Day 1 to Study Day 28) in the ADHD RS-5 inattention scale and total scale scores.
- Exploratory endpoints of this study include
  - Change (Study Day 1 to Study Day 28) in the PROMIS® Parent Proxy Item Bank v1.1 – Cognitive Function score
  - Change (Study Day 1 to Study Day 28) in the PROMIS® Pediatric Item Bank v1.0 – Cognitive Function score
  - Change (Study Day 1 to Study Day 28) in functional impairment as determined by change in ADHD-Rating Scale 5 Home Version relationship with significant others, peer relationships, academic functioning, behavioral functioning, homework functioning and self-esteem domains
  - Change (Study Day 1 to Study Day 28) in TOVA metrics other than ACS
    - Ex-Gaussian Tau Total
    - Commission Errors Standard Score H2
    - RT Variability Standard Score Total
    - RT Mean Standard Score H1
    - D-Prime Standard Score H2
    - Omission Errors Standard Score H2
  - Responder rates, defined as
    - TOVA-ACS
      - 1.0-point improvement from baseline after 4 weeks of AKL-T01 treatment
      - TOVA-ACS $\geq$ -1.0 at exit visit
    - $\geq$ 30% improvement in ADHD RS-5 total score from baseline after 4 weeks of AKL-T01 treatment

10.2 Analysis Populations for the Primary Study

Efficacy Population

The efficacy population will consist of any participant that is sent home with the AKL-T01 intervention that (for the purpose of this protocol):

1. Completes baseline assessments including TOVA
2. Completes their exit assessments including TOVA

Per Protocol (PP) Population

The PP population will be a subset of the efficacy population that meets definition for "adequate dosage" or "minimum acceptable exposure" to the game which will be defined as by completion of at least 60% of prescribed missions, equivalent to 72 of 120 missions (at least 6 missions/day × 5 days/week × 4 weeks) will be used in sensitivity analyses. Patients with any protocol deviations that could impact efficacy will be excluded from the PP population.

Safety Population

The safety population will consist of all participants who are exposed to AKL-T01 intervention.

10.3 Statistical Analysis

Details of statistical analyses will be provided in a statistical analysis plan. Analyses will be conducted using a complete case analysis for participants with sufficient data at baseline and follow-up timepoints. Missing data will not be imputed.

Baseline characteristics (socio-demographic, medical history, ADHD history, ADHD treatments etc.) of the study population, will be described using mean and standard deviation as well as median, minimum, maximum and interquartile range, as appropriate for continuous variables, and percentages for categorical variables. Compliance metrics will also be reported descriptively in the efficacy population using the same conventions.

10.3.1 Analysis of the Primary Endpoint

The primary endpoint is the change in TOVA-ACS calculated as the score at Day 28 minus the score at baseline for each participant. Since no missing data will be imputed, any patients without TOVA-ACS measurements at either timepoint will be excluded from the analysis. The primary analysis population will be the efficacy population, with analyses repeated for the PP populations as sensitivity analyses.
Descriptive statistics for TOVA-ACS baseline, follow-up and change scores will be calculated (mean, standard deviation, median, minimum, maximum, interquartile range) for patients with complete data at both timepoints. The null (H_0) and alternative (H_a)

hypotheses for assessing mean change (μ_d) in baseline to Day 28 measurements of TOVA-ACS are:

$$H_0: \mu_d = 0$$
$$H_a: \mu_d \neq 0$$

A two-sided one-sample *t*-test will be used to evaluate the mean change in TOVA-ACS with significance level of $\alpha = 0.05$. A statistically significant improvement will be defined by a 95% confidence interval for the mean change with lower bound greater than zero and corresponding *p*-value < 0.05. Given the large target sample size for this study, the *t*-test should be valid for assessing the change in TOVA-ACS relative to zero per the Central Limit Theorem even if the data deviates from a Normal distribution. Distribution of the change scores and use of equivalent nonparametric tests (i.e., Wilcoxon signed rank test) may be explored by visually assessing appropriate diagnostic plots (e.g., histogram, Q-Q plot) at the discretion of the investigators.

10.3.2 Analysis of Secondary Endpoints

Secondary endpoints for this study are change in ADHD RS-5 (1) inattention scale score and (2) total scale score, calculated as the score at Day 28 minus the score at baseline. Analysis of secondary endpoints will follow the methods described above for the primary endpoint with mean changes in respective ADHD RS-5 scores considered here as μ_d. A lower score on these measures indicates less severe symptoms, so a 95% confidence interval with upper bound less than zero will be considered a statistically significant improvement in these analyses.

Adjustment for multiplicity will be conducted for the primary and two secondary endpoints. Specific methodology will be described in the SAP.

10.3.3 Analysis of Exploratory Endpoints

Exploratory endpoints for this study include change in (1) PROMIS Parent Proxy Cognitive Function score, (2) PROMIS Pediatric Cognitive Function score, (3) ADHD RS-5 inattentive domains, and (4) specified TOVA metrics other than ACS. All differences will be calculated as the score at Day 28 minus the score at baseline and analyzed using the same methods as for the primary and secondary outcomes with appropriate tests of < or > 0 for each endpoint. Type I error will not be controlled for in analyses for exploratory endpoints, and only nominal *p*-values will be reported. Analyses will be conducted on the efficacy and repeated in the PP populations.

For responder analyses, patients achieving definitions of response for TOVA-ACS and ADHD RS-5 scores will be reported descriptively using counts and percentages. Additional responder analyses may be considered and will be described in the SAP if incorporated in the study.

10.3.4 Subgroup Analyses

Summary statistics for all efficacy endpoints will be provided for the following subgroups as additional exploratory analyses:

- Concomitant stimulant medication use: yes, no.
- Alternate definition(s) of treatment adherence, with categories to be determined post hoc

10.3.5 Adjunct Study

The primary endpoint for the adjunct study is change (Study Day 1 to Study Day 28) in the ADHD-RS-5 Home Version inattention scale and total scale scores. Exploratory endpoints include change (Study Day 1 to Study Day 28) in patient/caregiver perceived cognitive deficit reported using the PROMIS® Cognitive Function items; functional impairments as reported using the ADHD-RS-5 Home Version relationship with significant others, peer relationships, academic functioning, behavioral functioning, homework functioning and self-esteem domains; and responder analyses for ADHD RS-5. The adjunct study population will be analyzed separately from the primary study population and will have a separate SAP.

10.4 Sample Size

Sample sizes are calculated to test the null hypothesis of a zero-change from baseline in TOVA-ACS against the alternative of a positive change from baseline in TOVA-ACS with a one-sample t-test using EAST© Version 6.5. The significance level is defined by a one-sided α at 0.025, and a minimum power of 90% must be achieved. For the calculations it is assumed that the standard deviation σ in TOVA-API in this study population is as large as observed with TOVA-API in the study population of STARS-ADHD (SD=3.2). Assuming previously observed control-corrected effect size of $\mu=0.9$ and observed standard deviation of $\sigma=3.2$, a sample size of 135 is required to detect the effect with 90% power.

For the adjunct study, it is estimated that 85 participants are required to detect an effect size of 0.5 with 90% power at the 95% confidence level using a within-group t-test.

10.5 Safety Analysis

Treatment related adverse events for the AKL-T01 intervention will be presented. Any adverse events occurring during any phase of the study judged by the PI to be related to the intervention will be recorded and presented in a table. The Safety population will be used for this analysis that will include participants that began the at-home treatment phase for the intervention. The severity of each event will be evaluated by the PI and presented with the recorded events. If there are frequently occurring treatment related

adverse events, a frequency table will be included, to rank events from most frequent to most rare.

11. OPERATIONAL CONSIDERATIONS

11.1 Informed Consent

11.1.1 Consent/Assent Requirements

Informed consent will be obtained from all participant's caregivers (parent or legally authorized representatives). Informed consent is a process that is initiated before the individual agrees to participate in the study and continues throughout the individual's study participation. Consent forms will be Institutional Review Board (IRB) approved, and the participants' caregivers will be asked to read and review the document.

The informed consent process will be conducted and documented in the source document (including the date), and the form signed before the participant undergoes any study-specific procedures. Participants must be informed that participation is voluntary and that they may withdraw from the study at any time, without prejudice. The rights and welfare of the participants will be protected by emphasizing to them that the quality of their medical care will not be adversely affected if they decline to participate in this study.

Consent forms describing in detail the study treatment, study procedures, and risks are given to the participant's caregivers, and written documentation of informed consent is required before starting treatment/administering study treatment.

An IRB approved assent form describing the study, study procedures, and risks in age-appropriate detail are used to have a discussion about the study with participants. For participants able to read on their own, the form will be given to the participants to review prior to having the discussion. Site staff will discuss the study information with the participant and obtain participant signature. Written documentation of assent is required before starting treatment/administering study treatment. If a participant is unable to provide a handwritten signature, then the person obtaining assent must document that assent was provided verbally.

11.1.2 Consent/Assent Documentation

The following consent documentation will be utilized for this study:

- Caregiver Informed Consent – information provided to the legally authorized representative (LAR) (parents/caregivers) to give permission for pediatric participant

- Participant Assent– age-appropriate information provided to the pediatric participant, to agree to participation

11.1.3 Other Information and Materials Provided to Participants

Any other study related informational materials provided to participants and/or caregivers will be submitted for review and approval to the reviewing IRB according to IRB policies and procedure.

11.2 Confidentiality and Privacy

Participant confidentiality and privacy is strictly held in trust by the participating investigators, their staff, and the sponsor. The study protocol, documentation, data, and all other information generated will be held in strict confidence. No information concerning the study, or the data will be released to any unauthorized third party without prior written approval of the sponsor.

The study monitor, other authorized representatives of the sponsor, representatives of the IRB, and regulatory agencies may inspect all documents and records required to be maintained by the investigator, including but not limited to, medical records (office, clinic, or hospital) and pharmacy records for the participants in this study. The clinical study site will permit access to such records.

The study participant's caregiver contact information will be securely stored at each clinical site for internal use during the study. At the end of the study, all records will continue to be kept in a secure location for as long a period as dictated by the reviewing IRB, Institutional policies, or sponsor requirements.

Study participant research data, which is for purposes of statistical analysis and scientific reporting, will be transmitted to and stored at Akili. This will not include the participant's contact or identifying information. Rather, individual participants and their research data will be identified by a unique study identification number. The study data entry and study management systems used by clinical sites and by Akili Interactive's research staff will be secured and password protected. At the end of the study, all study databases will be coded and archived at Akili.

11.3 Trial Master File and Records Retention

The Trial Master File (TMF) is the collection of study documentation consisting of essential documents, which enable both the conduct of the clinical trial and the quality of the data produced to be evaluated.

Akili Clinical Operations is responsible for ensuring that complete and accurate documentation is prepared and maintained in compliance with good documentation practices and applicable federal, state, and local laws, rules and regulations.

TMF documentation includes all CRFs, regulatory documents and other study related documents (e.g., signed protocol and amendments, IRB correspondence and approval, approved participant consent forms, signed Investigator Statement form, clinical supplies receipts and distribution records).

TMF Documentation for this study will be maintained electronically in an electronic Trial Master File system (eTMF). Details of the oversight and management of the study TMF is detailed in the Trial Master File Management Plan.

The PI is responsible for filing of site essential documentation in the Investigator Site File. The PI will ensure all study documentation from the site will be available to Akili Clinical Operations and contracted vendors for review and monitoring.

Clinical operations and participating sites maintain all study documentation from the initiation of the study through 2 years after the latter of the following: either the date the study is complete or the date the records are no longer required to meet a regulatory approval application.

11.4 Protocol Deviations

A protocol deviation is defined as an event where the investigator or study personnel did not conduct the study according to the investigational plan.

All protocol deviations will be documented in the protocol deviation CRF. The IRB will be informed by the investigator of all protocol changes or deviations that occur in accordance with applicable regulations and the IRB's established procedures. No deviations from the protocol of any type will be made without complying with all the IRB/EC's established procedures.

11.5 Study Discontinuation and Closure

This study may be temporarily suspended or prematurely terminated if there is sufficient reasonable cause. Written notification, documenting the reason for study suspension or termination, will be provided by the sponsor to regulatory authorities, investigators, and study participants as applicable. If the study is prematurely terminated or suspended, the site principal investigator (PI) will promptly inform study participants, their reviewing IRB, and will provide the reasons for the termination or

suspension. Study participants will be contacted, as applicable, and be informed of changes to study visit schedule.

Circumstances that may warrant termination or suspension include, but are not limited to:

- Determination of unexpected, significant, or unacceptable risk to participants
- Demonstration of efficacy that would warrant stopping
- Insufficient compliance with protocol requirements
- Data that are not sufficiently complete and/or evaluable
- Determination that the primary endpoint has been met
- Determination of futility

Study may resume once concerns about safety, protocol compliance, and data quality are addressed, and satisfy the sponsor, IRB and/or Food and Drug Administration (FDA).

11.6 Audits and Inspections

The investigator will provide access to all study related documents and trial supplies for the purpose of sponsor oversight and quality assurance or inspection by local and federal regulatory authorities.

11.7 Conflict-of-Interest Policy

Any actual conflict of interest of persons who have a role in the design, conduct, analysis, publication, or any aspect of this study will be disclosed and managed. Furthermore, persons who have a perceived conflict of interest will be required to have such conflicts managed in a way that is appropriate to their participation in the design and conduct of this study.

12. REFERENCES

1. Biederman J, Chan J, Spencer TJ, et al. Evidence of A Pharmacological Dissociation Between The Robust Effects of Methylphenidate on Adhd Symptoms and Weaker Effects on Working Memory. *J Brain Sci*. 2015;1(2):43-53.

2. Klingberg T. Training and plasticity of working memory. *Trends Cogn Sci*. 2010;14(7):317-324. doi:10.1016/j.tics.2010.05.002

3. Kollins SH, DeLoss DJ, Cañadas E, et al. A novel digital intervention for actively reducing severity of paediatric ADHD (STARS-ADHD): a randomised controlled trial. *Lancet Digit Health*. 2020;2(4):e168-e178. doi:10.1016/S2589-7500(20)30017-0

4. Anguera JA, Brandes-Aitken AN, Rolle CE, et al. Characterizing cognitive control abilities in children with 16p11.2 deletion using adaptive ‘video game’ technology: a pilot study. *Transl Psychiatry*. 2016;6(9):e893-e893. doi:10.1038/tp.2016.178

5. Yerys BE, Bertollo JR, Kenworthy L, et al. Brief Report: Pilot Study of a Novel Interactive Digital Treatment to Improve Cognitive Control in Children with Autism Spectrum Disorder and Co-occurring ADHD Symptoms. *J Autism Dev Disord*. 2019;49(4):1727-1737. doi:10.1007/s10803-018-3856-7

6. Hughes S. A Guide to Using the Test of Variables of Attention (T. :4.

7. ADHD Rating Scale—5 for Children and Adolescents: Checklists, Norms, and Clinical Interpretation. Guilford Press. Accessed February 11, 2021. https://www.guilford.com/books/ADHD-Rating-Scale-5-for-Children-and-Adolescents/DuPaul-Power-Anastopoulos-Reid/9781462524877

8. Lai J-S, Wagner LI, Jacobsen PB, Cella D. Self-reported cognitive concerns and abilities: two sides of one coin? *Psychooncology*. 2014;23(10):1133-1141. doi:10.1002/pon.3522
