# Supplementary material for: Secondary analyses of sex differences in attention improvements across three clinical trials of a digital therapeutic in children, adolescents, and adults with ADHD
Source: BMC Public Health. 2024 Apr 29;24:1195. doi: 10.1186/s12889-024-18597-5 (PMC11057090; doi:10.1186/s12889-024-18597-5)
Supplement: Supplementary file 1 — Supplementary Material 1. [file 12889_2024_18597_MOESM1_ESM.zip › AKL-T01 Adult Study Protocol.docx.pdf]

Protocol Number: Akili-057

Official Title: A single arm, adaptive design, pivotal trial to assess the efficacy of AKL-T01, a novel digital intervention designed to improve attention, in adults diagnosed with Attention Deficit Hyperactive Disorder

Short Title: Software Treatment for Actively Reducing Severity of ADHD in Adults (STARS-ADHD-Adults)

Version: 3.0

Date: December 3 , 2021

Study Sponsor: Akili Interactive Labs, Inc.
Sponsor Address: 125 Broad Street, 4th Floor; Boston, MA 02110
Sponsor Representative: Vandana Menon, MD, PhD, VP Clinical Research

Protocol Approval Page

Akili-057: A single arm, adaptive design, pivotal trial to assess the efficacy of AKL-T01, a novel digital intervention designed to improve attention, in adults diagnosed with Attention Deficit Hyperactive Disorder

Version: 3.0 **Date of Issue:** December 3, 2021

We, the undersigned, have read, and approve this protocol and agree on its content.

SPONSOR REPRESENTATIVE	INVESTIGATOR
DocuSigned by: Vandana Menon EFA07A4EC9B440E...	
signature	signature
12/7/2021	
date	date
Vandana Menon, MD, PhD<br>VP Clinical Research	
printed name	printed name

Statement of Compliance

This trial will be carried out in accordance with International Conference on Harmonisation Good Clinical Practice (ICH GCP) and the following:

US Code of Federal Regulations (CFR) applicable to clinical studies (45 CFR Part 46, 21 CFR Part 50, 21 CFR Part 56, 21 CFR Part 312, and/or 21 CFR Part 812)

The protocol, informed consent forms, recruitment materials, and all participant materials will be submitted by the Investigator to the reviewing institutional review board (IRB) for review and approval. Approval of both the protocol and the consent form must be obtained before any participant is enrolled. Any amendment to the protocol will require review and approval by the IRB before the changes are implemented to the study. In addition, all changes to the consent form will be IRB approved; a determination will be made regarding whether a new consent needs to be obtained from participants who provided consent, using a previously approved consent form.

Protocol Version and Amendment Tracking

Version Number/Amendment	Approval Date
V 1.0 / Original	12Aug2021
V 2.0/ Amendment 1	19Oct2021
V 3.0/ Amendment 2	03Dec2021

Protocol Synopsis

Protocol Number	Akili-057
Protocol Title	A single arm, adaptive design pivotal trial to assess the efficacy of AKL-T01, a novel digital intervention designed to improve attention in adults diagnosed with Attention Deficit Hyperactive Disorder.
Sponsor	Akili Interactive Labs, Inc.
Non-Significant Risk Medical Device Investigation	This study fulfills the requirements for a non-significant risk medical device investigation as:<br>• It does not meet any of the criteria set forth in 21 CFR 812.3(m) to define a significant risk device<br>• Was previously determined by Institutional Review Boards to be non-significant risk device
Study Phase	Pivotal
Study Product	AKL-T01 Digital Treatment (AKL-T01)
Background	A diagnosis of Attention Deficit Hyperactive Disorder (ADHD) is often made in childhood however, in many cases, symptoms of impulsivity, inattention and hyperactivity persist into adulthood. Current pharmacologic therapy includes stimulants which while providing symptom relief are not effective for all patients and require careful and continuous titration to minimize side effects while maintaining clinical benefits. In addition, pharmacotherapy may not be suitable for some patients due to concerns about abuse, misuse, and diversion. Thus, there is an unmet need for non-pharmacologic treatment options for adult ADHD.<br><br>Digital therapeutics are emerging as a novel option to improve attention symptoms and attentional control.<br><br>AKL-T01 is a novel, highly immersive, digital therapeutic intervention which is currently FDA cleared as EndeavorRx®. EndeavorRx® is a digital therapeutic indicated to improve attention function as measured by computer-based testing in children ages 8-12 years old with primarily inattentive or combined-type ADHD, who have a demonstrated attention issue. AKL-T01 is deployed on mobile devices (i.e., tablets) and incorporates adaptive, simultaneous cognitive tasks in

	a consumer-grade action videogame-based platform with high-quality graphics and reward mechanisms.<br><br>AKL-T01 was studied in the Software Treatment for Actively Reducing Severity of ADHD (STARS-ADHD) study; a multi-center, randomized, double-blind, active-controlled study comparing AKL-T01 treatment to the use of a digital control in 348 pediatric participants aged 8-12 years, diagnosed with ADHD and a measurable impairment in objective attention function (as indicated by the TOVA Attention Performance Index (API) ≤ -1.8) who were not taking ADHD medication. 180 participants were randomized into AKL-T01, and 168 into the control group and both groups were instructed to engage with their intervention for approximately 25 minutes/day, 5 days/week for 4 weeks. The study successfully demonstrated a statistically significant improvement (p=0.006) between AKL-T01 and control on the primary endpoint (TOVA-API [a composite measure of objective attention] change from Baseline to after 4 weeks of treatment). This improvement in objective attention (TOVA-API) in the treatment group is indicative of a clinically meaningful benefit for AKL-T01 participants and is supported by the results of the study secondary endpoints, which all consistently trended towards improvement.<br><br>A subsequent open-label study, Software Treatment for Actively Reducing Severity of ADHD as Adjunctive Treatment to Stimulant (STARS-Adjunct), included children both on and off ADHD stimulant medication, ages 8-14 years old, and found comparable effects in these two cohorts on ADHD-related impairment and ADHD symptoms. Children with measurable attention impairment at baseline comparable to STARS-ADHD (TOVA API ≤ -1.8) also showed improvement in objective attention function.<br><br>In both studies, AKL-T01 demonstrated a favorable safety profile, with no serious adverse events observed.<br><br>This protocol will evaluate objective attention functioning and ADHD symptoms/impairments in adults with a diagnosis of ADHD (combined or inattentive subtype), stably on or off ADHD medication, after 6-weeks of AKL-T01 treatment.
Study Design	Multi-center, unblinded, uncontrolled adaptive design study
Study Objectives and Endpoints	1. The primary objective of this study is to evaluate the efficacy of AKL-T01 as determined by the change in a digitally assessed

	measure, Test of Variables of Attention (TOVA®) Attention Comparison Score (ACS), of sustained and selective attention, after 6 weeks of treatment with AKL-T01<br>2. A secondary objective of this study is to evaluate the change in ADHD symptoms, as determined by change in the ADHD Rating Scale-IV with adult prompts inattention sub-scale and total scale scores, after 6 weeks of treatment with AKL-T01<br>3. Exploratory objectives of this study are:<br>a. To evaluate change in the Adult ADHD Quality of Life (AAQoL) after 6 weeks of treatment with AKL-T01<br>b. To evaluate change in the Conner's Adult ADHD Rating Scale-Self Report: Short Version (CAARS-S:S) after 6 weeks of treatment with AKL-T01<br>c. To evaluate response rates for TOVA-ACS and ADHD-RS-IV after 6 weeks of treatment with AKL-T01<br>d. To evaluate changes in TOVA metrics other than ACS after 6 weeks of treatment with AKL-T01
Study Overview	Participants will have two on-site visits and an at-home intervention period:<br>1. Screening/Baseline visit (Day 1)<br>a. Confirm eligibility<br>b. Obtain informed consent<br>c. Provide training on the intervention<br>d. Obtain baseline data for study assessments<br>2. At-home treatment (Day 2-42)<br>a. Participants instructed to use the intervention at-home for approximately 25 minutes a day, 5 days a week<br>3. Exit/post-treatment visit (Day 42+3)<br>a. End of study assessments<br>b. Participant experience surveys
Inclusion/Exclusion Criteria	**<u>Inclusion</u>**<br>1. Adults 18 years and older<br>2. Diagnosis of ADHD combined or inattentive type, according to Diagnostic and Statistical Manual of Mental Disorders, Fifth Edition (DSM-5) as confirmed by Mini International Neuropsychiatric Interview (MINI) for Attention – Deficit / Hyperactivity Disorders Studies (Adult) 7.0.2<br>3. Stably on or off ADHD medications for ≥4 weeks prior to study enrollment and throughout the primary 6-week study

	4. Stably on or off psychoactive medications for ≥4 weeks prior to study enrollment and throughout the 6-week study<br>5. Baseline visit score on the ADHD-RS-IV of ≥ 24<br>6. Baseline visit score on the TOVA-ACS score ≤ -1.8<br>7. Estimated IQ score ≥80 as assessed by the Kaufmann Brief Intelligence Test, Second Edition (KBIT-II)<br>8. Access to and self-report of ability to connect wireless devices to a functional wireless network<br>9. Ability to follow written and verbal instructions (English) as assessed by the PI and/or study coordinator<br>10. Able to comply with all testing and study requirements<br>11. Completion of informed consent form<br>**Exclusion**<br>1. Current controlled or uncontrolled, comorbid psychiatric diagnosis with significant symptoms that in the opinion of the Investigator may confound study data/assessments.<br>2. Suicidality assessed using the Columbia-Suicide Severity Rating Scale (C-SSRS)<br>3. Motor condition (e.g., physical deformity of the hands/arms) that prevents game playing as reported by the participant or observed by the Investigator.<br>4. History of moderate or severe substance use disorder within the last 12 months prior to informed consent<br>5. History of seizures (excluding febrile seizures), significant tics, or a current diagnosis of Tourette’s Disorder.<br>6. Known sensitivity to playing video games, such as photo-sensitive epilepsy, light-headedness, dizziness, nausea, or motion sickness.<br>7. Color blindness as detected by Ishihara Color Blindness Test<br>8. Positive urine drug screen<br>9. Current or recent (3 months prior to screening) history of heavy smoking defined as the equivalent of greater than or equal to a pack of cigarettes a day<br>10. Any other medical condition that in the opinion of the Investigator may confound study data/assessments.<br>11. Participation in a clinical trial within 3 months prior to screening.<br>12. Previous exposure to Akili products within the 6 months prior to study enrollment<br>13. Plans to initiate new concomitant medications during the primary study, except for common over the counter (OTC) (e.g., ibuprofen, acetaminophen) and prescription medications (e.g., antibiotics) for minor transient ailments.

	14. Planned initiation of, or significant changes in frequency, of non-pharmacological behavioral therapy during the primary study<br>15. Planned initiation of, or significant changes in frequency of, non-pharmacological trainings with the aim to improve cognition by means of game or app-based cognitive trainings or neurofeedback, during the primary study
Treatment Regimen	Approximately 25 minutes/day for 5 days/week of AKL-T01 across the 6-week (42 day) treatment period.
Number of Sites	12-30
Number of Participants	A maximum of 325 completed participants will be enrolled. Recruitment will stop once a prespecified standard error of 0.277 is achieved for the primary endpoint (change in TOVA-ACS).
Duration of Primary Study Participation	45 days: Baseline visit, 42-day treatment phase, and an Exit Visit on day 42 (+3) days
Primary Endpoint	Change (Study Day 1 to Study Day 42) on the Attention Comparison Score, an overall composite score from TOVA 9 (TOVA-ACS). TOVA-ACS is equivalent to TOVA-Attention Performance Index (API)
Secondary Endpoint	Change (Study Day 1 to Study Day 42) in the administered ADHD Rating Scale-IV inattention scale and total scale scores
Exploratory Endpoints	• Change (Study Day 1 to Study Day 42) in Adult ADHD Quality of Life Questionnaire (AAQoL) total score and the Life Productivity subscale score<br>• Change (Study Day 1 to Study Day 42) in the Conner's Adult ADHD Rating Scale Short Self-Administered Version (CAARS-S:S) score<br>• Proportion of responders at end of treatment defined as<br>o ≥ 30% reduction in total ADHD-RS-IV scores<br>o 8-point change in AAQoL<br>• Change (Study Day 1 to Study Day 42) in TOVA metrics other than ACS
Sample Size Justification	Sample size was calculated to test the null hypothesis of a zero change in TOVA-ACS from baseline to Study Day 42 against the alternative of a positive change in TOVA-ACS over the same period with a one-sample paired t-test using SAS® version 9.4 (SAS Institute, Cary, North Carolina). The significance level was defined by a one-sided α=0.025 with a

	minimum power of 90% to detect a mean difference in pre- and post-intervention TOVA-ACS.<br><br>Given that the adult ADHD population may be more heterogeneous than the pediatric ADHD population, a standard deviation σ of 1.5 times that observed in STARS-ADHD (SD=3.2) was assumed . Using the previously observed control-corrected effect size of μ=0.9 and σ=4.8, a sample size of 301 participants is required to detect the effect with 90% power.
Statistical Analysis	The primary analysis population will be the efficacy population, consisting of all participants sent home with the AKL-T01 intervention who complete both baseline and exit assessments. A per protocol (PP) population will also be defined as the subset of the primary analysis population who meet criteria for “adequate dosage” or “minimum acceptable exposure” to treatment. The safety population will include all participants that were exposed to the intervention regardless of whether they completed endpoint assessments.<br><br>Baseline characteristics (socio-demographic, medical history, ADHD history, ADHD treatments, etc.) of the study population, will be described using mean and standard deviation as well as median, minimum, maximum and interquartile range, as appropriate for continuous variables, and counts with percentages for categorical variables.<br><br>A two-sided one-sample t-test at the α=0.05 confidence level will be used to evaluate the whether the change in baseline to Day 42 measurements differs from zero for TOVA-ACS and TOVA metrics, the ADHD-RS-IV inattention subscale and total scale scores, AAQoL total and life productivity subscale scores, and CAARS-S:S score.<br><br>Treatment related adverse events for the AKL-T01 intervention will be presented. Any adverse events occurring during any phase of the study judged by the investigator to be related to the intervention will be recorded and presented in a table. The safety population will be used for this analysis that will include participants who were exposed to the intervention. The severity of each event will be evaluated by the PI and presented with the recorded events. If there are frequently occurring treatment related adverse events, a frequency table will be included, to rank events from most frequent to most rare.

Table of Contents

Protocol Approval Page **2**

Statement of Compliance **3**

Protocol Version and Amendment Tracking **4**

Protocol Synopsis **5**

Abbreviations **13**

1. INTRODUCTION **14**

1.1 Background 14
1.2 Clinical Experience with Study Agent 14

2. OBJECTIVES **15**

3. STUDY POPULATION **16**

3.1 Inclusion Criteria 16
3.2 Exclusion Criteria 16
3.3 Screen Failures 17

4. STUDY DESIGN **17**

4.1 Overview 17
4.2 Study Schedule of Procedures 18
4.3 Screening Visit 19
4.4 Baseline Procedures 19
4.5 At-Home Treatment Phase 20
4.6 Exit/Post-Treatment Visit 21
4.7 End of Study Definition 21
4.7.1 Study Completion 21
4.7.2 Participant Withdrawal or Discontinuation 21
4.7.3 Lost to Follow-Up 22

5. STUDY INTERVENTION **23**

5.1 Study Interventions Description 23
5.2 Study Intervention Compliance 24
5.3 Potential Benefits/Risks of Study 24
5.4 Withdrawal/Discontinuation of Study Interventions 25

6. EFFICACY ASSESSMENTS **25**

6.1 TOVA 25
6.2 ADHD Rating Scale-IV with Adult Prompts 25
6.3 Adult ADHD Quality-of-Life Scale (AAQoL) 26
6.4 Conner's Adult ADHD Rating Scales-Self-Report: Short Version (CAARS-S:S) 26
6.5 Measures to Minimize Bias 26

7. CONCOMITANT MEDICATIONS AND THERAPIES **27**

8. SAFETY **27**

8.1 Definitions of Adverse Device (Software) Effect 28
8.1.1 Adverse Device (Software) Effect (ADE) 28
8.1.2 Unanticipated Adverse Device Effects (UADEs) 28
8.2 Previously Noted Adverse Device (Software) Effects 29
8.3 Collection of Adverse Device (Software) Effects 29
8.4 Assessment of Adverse Device (Software) Effect 30
8.4.1 Relationship to Study Intervention 30
8.4.2 Severity of Adverse Device (Software) Effects 30
8.4.3 Outcome of Unanticipated Adverse Device (Software) Effects 30
8.5 Reporting of Adverse Device (Software) Effects 31
8.5.1 Site Reporting 31
8.5.2 Institutional Review Board Reporting 31
8.5.3 Regulatory Reporting 31

9. DATA AND SAFETY MONITORING 32

9.1 Study Data Collection and Data Entry 32
9.2 Study Dataset 32
9.3 Endpoint Determination 33
9.4 Safety Monitoring 33
9.5 Clinical Monitoring and Quality Control (QC) 33
9.6 Future Use of Stored Data 33

10. STATISTICAL CONSIDERATIONS 33

10.1 Endpoints 34
10.2 Analysis Populations 35
10.3 Statistical Analysis 36
10.3.1 Analysis of the Primary Endpoint 36
10.3.2 Analysis of Secondary Endpoints 36
10.3.3 Analysis of Exploratory Endpoints 37
10.4 Sample Size 37
10.5 Safety Analysis 38

11. OPERATIONAL CONSIDERATIONS 38

11.1 Informed Consent 38
11.1.1 Consent/Assent Requirements 38
11.1.2 Other Information and Materials Provided to Participants 39
11.2 Confidentiality and Privacy 39
11.3 Trial Master File and Records Retention 39
11.4 Protocol Deviations 40
11.5 Study Discontinuation and Closure 40
11.6 Audits and Inspections 41
11.7 Conflict-of-Interest Policy 41

12. REFERENCES 42

Abbreviations

AAQoL	Adult ADHD Quality of Life Questionnaire
ACS	Attention Comparison Score (current/updated name of API)
ADHD	Attention Deficit Hyperactivity Disorder
ADHD Rating Scale-IV	Attention Deficit Hyperactivity Disorder Rating Scale IV
API	Attention Performance Index (previous name of the ACS)
APP	Application
CAARS-S:S	Conner's Adult ADHD Rating Scales-Self-Report: Short Version
CFR	Code of Federal Regulations
C-SSRS	Columbia-Suicide Severity Rating Scale
CONSORT	Consolidated Standards of Reporting Trials
CRF	Case Report Form
DSM-5	Diagnostic and Statistical Manual of Mental Disorders, Fifth Edition
eCRF	Electronic Case Report Form
FDA	Food and Drug Administration
GCP	Good Clinical Practice
HIPAA	Health Insurance Portability and Accountability Act
ICF	Informed Consent Form
ICH	International Conference on Harmonisation
IDE	Investigational Device Exemption
IRB	Institutional Review Board
KBIT-II	Kaufmann Brief Intelligence Test, Second Edition
MAOI	Monoamine oxidase inhibitor
MINI	Mini International Neuropsychiatric Interview (MINI) *Screen* 7.0.2
OTC	Over the counter
PI	Principal Investigator
QC	Quality Control
SAP	Statistical analysis plan
SCID-5	Structured Clinical Interview for DSM-5
SE	Standard error
SSRI	Selective serotonin reuptake inhibitor
STARS-ADHD	Software Treatment for Actively Reducing Severity of ADHD as Adjunctive Treatment to Stimulant
TE-ADE	Treatment-Emergent Adverse Device Effect
TOVA	Test of Variables of Attention
UADE	Unanticipated Adverse Device Effect
UP	Unanticipated problems

1. INTRODUCTION

1.1 Background

A diagnosis of Attention Deficit Hyperactive Disorder (ADHD) is often made in childhood however, in many cases, symptoms of impulsivity, inattention and hyperactivity persist into adulthood.[1] Current pharmacologic therapy includes stimulants which while providing symptom relief are not effective for all patients and require careful and continuous titration to minimize side effects while maintaining clinical benefits.[2] Pharmacotherapy may not be suitable for some patients due to concerns about abuse, misuse, and diversion.[3] There is recent evidence that pharmacological treatment may not show optimal benefits in some cognitive domains.[4] Thus, there is an unmet need for non-pharmacologic treatment options for adult ADHD.

Digital therapeutics are emerging as a novel option to improve attention symptoms and attentional control. Computerized cognitive training programs have shown some promise in improving working memory and attention in ADHD populations.[5]

AKL-T01 is a novel, highly immersive, digital therapeutic intervention which is currently FDA cleared as EndeavorRx®. EndeavorRx® is a digital therapeutic indicated to improve attention function as measured by computer-based testing in children ages 8-12 years old with primarily inattentive or combined-type ADHD, who have a demonstrated attention issue. Patients who engage with EndeavorRx® demonstrate improvements in Test of Variables of Attention (TOVA®), a digitally assessed measure of sustained and selective attention, and may not display benefits in typical behavioral symptoms, such as hyperactivity. EndeavorRx® should be considered for use as part of a therapeutic program that may include clinician-directed therapy, medication, and/or educational programs, which further address symptoms of the disorder. It is not intended as a stand-alone therapeutic, nor is it a substitute for a child's medication.

AKL-T01 is deployed on mobile devices (i.e., tablets) and incorporates adaptive, simultaneous cognitive tasks in a consumer-grade action videogame-based platform with high-quality graphics and reward mechanisms.

This protocol will extend existing data supporting AKL-T01's effects on objective attention functioning and ADHD symptoms/impairment to adults with ADHD.

1.2 Clinical Experience with Study Agent

AKL-T01 was studied in the Software Treatment for Actively Reducing Severity of ADHD (STARS-ADHD) study; a multi-center, randomized, double-blind, active-controlled study comparing AKL-T01 treatment to the use of a digital control in 348 pediatric participants

aged 8-12 years, diagnosed with ADHD and a measurable impairment in objective attention function (as indicated by the TOVA® Attention Performance Index [API] ≤ -1.8) who were not taking ADHD medication. 180 participants were randomized into AKL-T01, and 168 into the control group and both groups were instructed to engage with their intervention for approximately 25 minutes/day, 5 days/week for 4 weeks. The study successfully demonstrated a statistically significant improvement (p=0.006) between AKL-T01 and control on the primary endpoint (TOVA-API [a composite measure of objective attention] change from baseline to after 4-weeks of treatment).[6]

A subsequent open-label study Software Treatment for Actively Reducing Severity of ADHD as Adjunctive Treatment to Stimulant (STARS-Adjunct) included children ages 8-14 years old, both on and off stimulant ADHD medication, and found comparable effects in these two cohorts on ADHD-related impairment and ADHD symptoms. Children with measurable attention impairment at baseline comparable to STARS-ADHD TOVA Attention Comparison Score (ACS ≤-1.8) showed improvement in objective attention function. In both of these studies, AKL-T01 demonstrated a favorable safety profile, with no serious adverse events observed.[7]

Two separate studies of 20 and 19 children with ADHD, Sensory Processing Disorder and Autism Spectrum Disorder, where AKL-T01 was used for a 4-week treatment period and showed improvements in attention measures and attention-related ADHD symptoms.[8,9]

2. OBJECTIVES

- The primary objective of this study is to evaluate the efficacy of AKL-T01 as determined by the change in a digitally assessed measure, Test of Variables of Attention (TOVA®) Attention Comparison Score (ACS), of sustained and selective attention, after 6 weeks of treatment with AKL-T01
- A secondary objective of this study is to evaluate the change in ADHD symptoms, as determined by change in the ADHD Rating Scale-IV (ADHD-RS-IV) inattention sub-scale and total scale scores, after 6 weeks of treatment with AKL-T01
- Exploratory objectives of this study are:
    - To evaluate change in the Adult ADHD Quality of Life (AAQoL) after 6 weeks of treatment with AKL-T01
    - To evaluate change in the Conner's Adult ADHD Rating Scale-Self Report: Short Version (CAARS-S:S) after 6 weeks of treatment with AKL-T01
    - To evaluate response rates for ADHD-RS-IV and AAQoL after 6 weeks of treatment with AKL-T01; and
    - To evaluate changes in TOVA metrics other than ACS after 6 weeks of treatment with AKL-T01.

3. STUDY POPULATION

3.1 Inclusion Criteria

To be eligible for this trial, participants must meet all the following criteria:

1) Adults 18 years and older
2) Diagnosis of ADHD combined or inattentive type, according to Diagnostic and Statistical Manual of Mental Disorders, Fifth Edition (DSM-5) as confirmed by Mini International Neuropsychiatric Interview (MINI) for Attention – Deficit / Hyperactivity Disorders Studies (Adult) 7.0.2
3) Stably on or off ADHD medication for ≥4 weeks prior to study enrollment and throughout the primary 6-week study
4) Stably on or off psychoactive medications for ≥4 weeks prior to study enrollment and throughout the 6-week study
5) Baseline visit score on the ADHD-RS-IV of ≥ 24
6) Baseline visit score on the TOVA-ACS score ≤ -1.8
7) Estimated IQ score ≥80 as assessed by the Kaufmann Brief Intelligence Test, Second Edition (KBIT-II)
8) Access to and self-report of ability to connect wireless devices to a functional wireless network
9) Ability to follow written and verbal instructions (English) as assessed by the PI and/or study coordinator
10) Able to comply with all testing and study requirements
11) Completion of informed consent form

3.2 Exclusion Criteria

If a participant meets any of the following criteria, he or she may not be enrolled in the study:

1) Current controlled or uncontrolled, comorbid psychiatric diagnosis with significant symptoms that in the opinion of the Investigator may confound study data/assessments.
2) Suicidality assessed using the Columbia-Suicide Severity Rating Scale (C-SSRS)
3) Motor condition (e.g., physical deformity of the hands/arms) that prevents game playing as reported by the participant or observed by the Investigator.
4) History of moderate or severe substance use disorder within the last 12 months prior to informed consent
5) History of seizures (excluding febrile seizures), significant tics, or a current diagnosis of Tourette’s Disorder.
6) Known sensitivity to playing video games, such as photo-sensitive epilepsy, light-headedness, dizziness, nausea, or motion sickness.
7) Color blindness as detected by Ishihara Color Blindness Test

8) Positive urine drug screen
9) Current or recent (3 months prior to screening) history of heavy smoking defined as the equivalent of greater than or equal to a pack of cigarettes a day
10) Any other medical condition that in the opinion of the Investigator may confound study data/assessments.
11) Participation in a clinical trial within 3 months prior to screening.
12) Previous exposure to Akili products within the 6 months prior to study enrollment
13) Plans to initiate new concomitant medications during the primary study, except for common over the counter (OTC) (e.g., ibuprofen, acetaminophen) and prescription medications (e.g., antibiotics) for minor transient ailments.
14) Planned initiation of, or significant changes in frequency, of non-pharmacological behavioral therapy during the primary study
15) Planned initiation of, or significant changes in frequency of, non-pharmacological trainings with the aim to improve cognition by means of game or app-based cognitive trainings or neurofeedback, during the primary study

3.3 Screen Failures

Screen failures are defined as participants who consent to participate in the clinical trial but are not subsequently enrolled into the study or administered study treatment. A minimal set of information is required to ensure transparent reporting of participant screen failures, to meet the Consolidated Standards of Reporting Trials (CONSORT) publishing requirements and to respond to queries from regulatory authorities. Minimal information includes demography, screen failure details, and eligibility criteria.

4. STUDY DESIGN

4.1 Overview

This study is a multi-center, unblinded/non-controlled adaptive design study to evaluate objective attention functioning and ADHD symptoms and impairments in adults with a diagnosis of ADHD (combined or inattentive subtype), stably on or off ADHD medication, after 6 weeks of AKL-T01 treatment.

Up to 325 completed participants from 12-30 sites will be enrolled. Participants who do not complete both baseline and exit visits will be replaced. Enrollment is competitive and there are no caps per site.

Study participation is expected to be 45 days: Baseline visit, 42-day treatment phase, and an Exit Visit on day 42 (+3) days.

Participants will have two on-site visits: a baseline visit and exit/post-treatment visit for study assessments. Participants will complete a 42 day at-home treatment phase in between the baseline and exit visit.

4.2 Study Schedule of Procedures

Procedure	Screening Visit Day 1	Baseline Procedures Day 1 [1}	At-Home Treatment Phase Days 2-42	Exit / Post-treatment Visit Day 42 (+3)
**Informed Consent**	X			
**Demographics**	X			
**ADHD Module Mini 7.0.2**	X			
**Urine tests**	X			X
**TOVA (Version 9) [2]**	X			X
**KBIT-II**	X			
**Ishihara Color Blindness Test**	X			
**C-SSRS**	X			
**ADHD-RS-IV with Adult Prompts**	X			x
**MINI Screen**		X		
**Medical History**		X		
**ADHD History**		X		
**AAQoL**		X		X
**CAARS-S:S**		X		X
**Prior and Concomitant Medications/Therapies [3]**		X	◄──────►	◄──────►
**Assign iPad-Mini**		X		
**Intervention Training**		X		
**AKL-T01 Treatment [4]**			◄──────►	◄──────►
**AKL-T01 Verify Data Upload**		X		
**AKL-T01 compliance check [5]**			◄──────►	◄──────►
**Collect iPad-Mini and charger**				X
**Participant Experience Survey**				X
**Treatment Emergent Adverse Device Effect**		◄──────►	◄──────►	◄──────►
	[1] Baseline visit may be held on a different day than the screening visit, at the discretion of study staff<br>[2] Assessments may be conducted on a different day from screening visit based on site discretion<br>[3] Capture changes to medications and dosage, include non-pharmacological therapies, as reported			

	[4] 25 minutes/day for at least 5 days/week of AKL-T01 across the 6-week (42 day) treatment period<br>[5] Compliance check throughout 42-day treatment

4.3 Screening Visit

Participants will be instructed to avoid alcohol, marijuana and caffeine (e.g. coffee, tea, sodas, energy drinks, caffeine pills, etc) for 4-6 hours prior to the screening visit, and to delay (at the discretion of the study doctor) taking their ADHD medication until after the visit, which is an on-site visit, during which the following study procedures and assessments will be performed:

- Informed consent will be obtained from the participant
- Preferred method of contact will be collected
- Demographic information will be collected
- ADHD Module of Mini 7.0.2 to establish inattentive or combined ADHD diagnosis
- A urine drug screen
- Test of Variables of Attention (TOVA)
  - Participants must refrain from using nicotine products for at least an hour prior to TOVA
  - Participants should have a minimum 10-minute break from previous study procedures before TOVA administration
- C-SSRS
- ADHD-RS IV with adult prompts
- KBIT-II
- Ishihara Color Blindness Test
- Assessment of study eligibility

4.4 Baseline Procedures

Baseline procedures are to be conducted on the same day as the screening visit. If a participant meets all eligibility criteria, the following study procedures and assessments will be performed:

- Mini International Neuropsychiatric Interview (MINI) *Screen* 7.0.2
- Medical history
- ADHD history
- Review of prior and concomitant medications (including dosages), behavioral therapies or cognitive and related training, including EEG neuro feedback training
- AAQoL Scale
- CAARS-S:S Scale

- An iPad Mini, loaded with AKL-T01 software and instructions for use will be assigned and provided to the participant. Intervention Training will be given to the participant delivering proper device and software usage.
- The participant will initiate their first treatment day at the clinic. At a minimum, the participant must complete the tutorial and analysis missions in-clinic.
- Site staff will verify data upload on the Akili Dashboard to confirm that the treatment registered.
- Participants will be instructed to notify the site during the At-Home Treatment portion if there are any changes to their Concomitant Medications or if any Treatment Emergent Adverse Device Effects (TE-ADEs) occur.

The following materials are provided to the participant to take home:

- Signed informed consent
- The iPad they were assigned
- Charger
- Quick instructions: Set up of the participants' local Wi-Fi network for internet access to push play and compliance data to Akili secure servers, and completion of at-home intervention for the assigned 25 minutes per day, during at least 5 days of each calendar week.

4.5 At-Home Treatment Phase

Participants will initiate at-home play with the AKL-T01 treatment. Participants will be asked to complete AKL-T01 treatment per the Study Application Usage instructions given during the Intervention Training.

To access the AKL-T01 intervention, participants will log into their assigned device with a unique user ID and password. Details about performance and duration of engagement will be automatically recorded and uploaded to Akili central servers when the device is connected to Wi-Fi.

Compliance checks will be performed throughout the treatment phase. Automatic compliance emails will be sent to study staff daily. Study staff will contact active participants who do not complete the required treatment after 48 hours of consecutive non-compliance. The software will also automatically alert participants when they have not interacted with the application for more than 24 hours.

Participants will be instructed to notify the site during the At-Home Treatment phase if there are any changes to their Concomitant Medications or if any Treatment Emergent Adverse Device Effects (TE-ADEs) occur. A Participant Card will be provided which contains spaces for the participants to record ADE start/stop dates, concomitant medication changes, and the date of the scheduled exit/post-treatment visit. The

Participant Card will be submitted to the site at the Exit/Post-treatment visit. The site will transcribe any changes that may have occurred during the treatment portion of the study into the Electronic Data Capture System (EDC).

4.6 Exit/Post-Treatment Visit

The exit visit should be scheduled at approximately the same time of day (i.e., morning or afternoon) as the Baseline visit and, participants should be instructed to avoid alcohol marijuana and caffeine for 4-6 hours prior to the visit and, at the discretion of the study doctor, to delay taking their ADHD medication until after the visit.

Participants will arrive at the study clinic with the assigned iPad-Mini, charger, and Participant Card.

The same qualified study personnel who performed the baseline visits must perform all study assessments for the Exit Visit.

- A urine drug screen
- TOVA
  - Participants must refrain from using nicotine products for at least an hour prior to TOVA
  - Participants should have a minimum 10-minute break from previous study procedures before TOVA administration
- ADHD-RS-IV with adult prompts
- AAQoL
- CAARS-S:S
- Review of concomitant medications and therapies
- Participant Experience Survey, regarding general game-play experience and satisfaction with treatment, will be completed by participants.

4.7 End of Study Definition

4.7.1 Study Completion

A participant is considered to have completed the study if he or she has completed all phases of the study including the Exit Visit, at Day 42 (+3).

4.7.2 Participant Withdrawal or Discontinuation

<u>Participant Withdrawal</u>
Participants are free to withdraw from participation in the study at any time upon request, for any reason without prejudice.

Investigator Discontinuation of Participant

Participants may be discontinued from the study at the discretion of the investigator if medically necessary or if any untoward effects occur. In addition, a participant may be discontinued by the investigator (in consultation with the sponsor as necessary) for the following reasons:

- Significant study treatment non-compliance [defined as <30 missions played] with prescribed study treatment observed in the first two weeks following enrollment
- If any clinical adverse device effect (ADE), or other medical condition or situation occurs such that continued participation in the study would not be in the best interest of the participant
- Disease progression worsening to the extent that it requires discontinuation of the study treatment
- If the participant meets an exclusion criterion (either newly developed or not previously recognized) that precludes further study participation
- Or otherwise violates the study plan, or for administrative and/or other safety reasons

The reason for participant withdrawal/discontinuation from the study will be recorded on the study Case Report Form (CRF).

Participants who sign the informed consent form but do not receive the study treatment will be counted as screen failures. Participants who sign the informed consent form and receive the study treatment will be counted as enrolled participants, even if they subsequently withdraw or are discontinued from the study.

The investigator or designee will notify the sponsor or their designee immediately when a participant has withdrawn or been discontinued from the study because of an adverse event. When a participant withdraws or is discontinued from the study before study completion, all applicable activities scheduled for the final study visit should be performed at the time of discontinuation. Any adverse events that are present at the time of withdrawal/discontinuation should be reported and followed up in accordance with the safety requirements outlined in the Adverse Device (Software) Effect Reporting and Follow-Up section.

Participants may withdraw by contacting the investigator or study staff at their site.

4.7.3 Lost to Follow-Up

A participant will be considered lost to follow-up if they fail to return for the final study visit and are unable to be contacted by the study site staff.

The following actions must be taken if a participant fails to return to the clinic for a required study visit:

- The site will attempt to contact the participant and reschedule the missed visit within a one-week window of the missed visit and counsel the participant on the importance of maintaining the assigned visit schedule and ascertain if the participant wishes to and/or should continue in the study.
- Before a participant is deemed lost to follow-up, the investigator or designee will make every effort to regain contact with the participant (where possible, 3 telephone calls and, if necessary, a certified letter to the participant's last known mailing address or local equivalent methods). These contact attempts should be documented in the participant's study file.
- Should the participant continue to be unreachable, they will be considered to have withdrawn from the study with a primary reason of loss to follow-up.

5. STUDY INTERVENTION

5.1 Study Interventions Description

AKL-T01 is a digital therapeutic built using Akili's proprietary algorithm (Selective Stimulus Management Engine [SSME™]) designed to train interference management at an adaptive and personalized high degree of difficulty. Interference is instantiated through a video game-based interface displaying two tasks that are to be done in parallel (multitasking); a perceptual discrimination targeting task in which users respond to the instructed stimulus targets and ignore the stimulus distractors (similar to a Go–No-Go task), and a sensory motor navigation task in which users continuously adjust their location to interact with or avoid positional targets. Performance in each task is assessed during single and multitask conditions. As users proceed through the treatment, periodic recalibration occurs to maintain an optimal difficulty level.

Participants should allow approximately 25 minutes of time to complete each daily treatment, which consists of playing between 6-8 missions per day. Participants will be instructed to play at least 5 days per week, during the 6 weeks of the treatment phase of the study. Participants may initiate play immediately after receiving their study device. Specifics about which days and times can be used for play can be chosen by each participant based on their schedules and may vary among participants as long as the above play requirements are met. Actual time of play will be captured by the software.

The device should be connected to the participant's home Wi-Fi network or cellular service. If home Wi-Fi or cellular service is not available, the participants need to establish a plan where the device will be connected to remote Wi-Fi on a periodic basis during the treatment. To initiate play, the participant taps on the game icon displayed on the screen and follows the in-program instructions. Separate electronic instructions for use (IFUs) will also be accessible on the study device.

For any calendar day, the treatment will automatically lock after the participants completes their allocated maximum number of daily missions, and no further play will be allowed until the next calendar day. Access to the treatment will be disabled at the end of the study.

5.2 Study Intervention Compliance

AKL-T01 automatically captures gameplay compliance and uploads these data directly to a central server when the iPad is connected to Wi-Fi. The server will automatically push daily compliance emails to the clinical sites. Based on these emails, study staff can then reach out to participants (via the preferred contact method captured in the CRF) to troubleshoot technical problems and/or encourage more play. The rule for compliance outreach is as follows:

Participants that fail to record any treatment over two consecutive days will be contacted to troubleshoot potential Wi-Fi connection problems.

Participants that fail to record at least 15 missions of AKL-T01 over 7 days will be contacted and reminded to continue their study intervention.

Additionally, AKL-T01 contains built-in features that remind the participant to play each day.

5.3 Potential Benefits/Risks of Study

The commercial version of AKL-T01, EndeavorRx® is a digital therapeutic indicated to improve attention function as measured by computer-based testing in children ages 8-12 years old with primarily inattentive or combined-type ADHD who have a demonstrated attention issue. Patients who engage with EndeavorRx® demonstrate improvements in a digitally assessed measure of sustained and selective attention, TOVA®, and may not display benefits in typical behavioral symptoms, such as hyperactivity. EndeavorRx® should be considered for use as part of a therapeutic program that may include clinician-directed therapy, medication, and/or educational programs, which further address symptoms of the disorder. EndeavorRx® is not intended to be used as a stand-alone therapeutic and is not a substitution for medication.

Of 538 participants using EndeavorRx® (AKL-T01), 50 participants (9.3%) experienced treatment-related adverse events (probable, likely), and three participants experienced treatment-related adverse events with the digital control, in studies where a control was used. EndeavorRx® associated adverse events included frustration (6.1%), headache (1.3%), dizziness (0.6%), emotional reaction (0.4%), nausea (0.4%), and aggression

(0.2%). All adverse events were generally transient. Only 3 events led to device discontinuation, and no subject reported lasting or irreversible effects after discontinuation. To date, there have been no serious adverse events reported across several studies with the treatment software.

If a participant experiences frustration, emotional reaction, dizziness, nausea, headache, eyestrain, or joint pain while playing they should pause the treatment and if the problem persists, they should contact their healthcare provider. If a participant experiences a seizure, they should stop the treatment and contact their healthcare provider.

5.4 Withdrawal/Discontinuation of Study Interventions

Participants are free to withdraw from study intervention at any time upon request, for any reason.

An investigator may discontinue a participant from the study intervention for the same reasons as detailed in the Participant Discontinuation/Withdrawal from the Study section.

If a participant withdraws or is discontinued from study intervention, they will be subsequently withdrawn/discontinued from the study as described in the Participant Discontinuation/Withdrawal from the Study section.

6. EFFICACY ASSESSMENTS

6.1 TOVA

The TOVA® is an FDA-cleared, continuous performance test measuring attention and inhibitory control used in the evaluation of ADHD treatments.[10] The test provides several objective metrics related to attention function and generates an Attention Comparison Score. Age and gender-matched norms are available for children and adults 4 years and older.

6.2 ADHD Rating Scale-IV with Adult Prompts

The ADHD-RS-IV is a rating scale based on the diagnostic criteria for ADHD as described in the fourth edition of the Diagnostic and Statistical Manual of Mental Disorders (DSM-IV).[11] It consists of two symptom subscales, Inattention (9 items) and Hyperactivity-Impulsivity (9 items), as well as a Total Scale (18 items). Scoring is based on a 4-point

Likert-type severity scale ranging from 0 (none) to 3 (severe). A 25 to 30% change (reduction) in ADHD-RS-IV scores is considered a clinically meaningful improvement.[12]

6.3 Adult ADHD Quality-of-Life Scale (AAQoL)

The AAQoL is a 29-item, self-report measure of functioning in a variety of life domains and adult roles.[13] Items are rated on a 5-point scale that are transformed into a 0–100 scale, with higher scores reflecting higher quality of life. Scores are calculated for Life Productivity, Psychological Health, Life Outlook, and Relationships, in addition to a Total score. An analysis by Goodman et. Al., established a threshold for discrimination for changes in health-related QoL of approximately 0.5 SD corresponding to an 8-point improvement.[12]

6.4 Conner's Adult ADHD Rating Scales-Self-Report: Short Version (CAARS-S:S)

The CAARS provides an assessment of symptoms and behaviors associated with ADHD in adults aged 18 and older.[14] Normative data for the self-report forms consist of 1,026 non-clinical adults (ages 18-80). The short versions of the self-report form (CAARS-S:S) contain 26 items that are abbreviated versions of the factor-derived subscales that appear on the long forms and incorporate the ADHD index and the Inconsistency Index. The short versions require about 10 minutes to complete.

6.5 Measures to Minimize Bias

Bias opportunities should be minimized by the removal of rater variability. Every effort should be made, throughout the course of the trial, to maintain the consistency of rating staff among participants.

To minimize the unintentional bias of study data and to maintain data consistency, clinical rater assessments for each participant should be completed at the approximately same time of day throughout the course of the study. The TOVA assessment should also be completed at the same time of the day and a break should be allowed before the TOVA.

Every effort must be made to ensure that raters of all rating scales/assessments are kept consistent among participants throughout trial participation. Each rating scale/assessment for the participant should be administered by the same rater for the duration of the trial. If a rater leaves a clinical site for any reason the site should replace the rater as quickly as possible, and this replacement rater will also be expected to complete rater training (if not previously trained). The replacement rater should review

the participant's source documents for previously completed rating assessments, to become familiar with the former rater's evaluations.

7. CONCOMITANT MEDICATIONS AND THERAPIES

ADHD medications administered prior to treatment and until the final study visit will be recorded in the CRFs.

Participants may be enrolled into the study currently on stimulant or other types of ADHD medication, provided that the participant's medication is prescribed for the treatment of ADHD symptoms and dosage has been stable for ≥ 4 weeks prior to the Baseline Visit. Each participant's ADHD medication dosage should remain stable as possible during the primary study. Any titrations (including switching medications) of the participant's ADHD medication during their participation in the trial will be documented within the CRFs.

Participants may be enrolled into the study on psychoactive medications (e.g., SSRIs, MAOIs, tricyclic antidepressants, antipsychotics etc.), provided that the participants' medication and dosage has been stable for ≥ 4 weeks prior to the Baseline Visit. A participant's medication dosage should remain s as stable as possible during the course of the trial. Any titrations (including switching medications) of the participant's medication during the course of their participation in the trial will be documented within the CRFs.

Initiation of or significant changes in non-pharmacological behavioral therapy, or non-pharmacologic trainings with game or app-based cognitive training or neurofeedback, (including but not limited to Cogmed or Attentive) for ADHD, is not permitted during the study. Participants actively engaged in non-pharmacological treatment at screening may be eligible if they meet all inclusion criteria and do not undergo changes to their treatment during the study. The participant should inform the Investigator if they intend to change their non-pharmacological therapy during the 6 weeks of the study.

8. SAFETY

Safety assessments will include the evaluation of adverse device effects (ADEs). Other Assessments will include unanticipated problems (UP).

8.1 Definitions of Adverse Device (Software) Effect

8.1.1 Adverse Device (Software) Effect (ADE)

Defined as an adverse event related to the use of an investigational medical treatment delivered through software application. This includes any adverse event resulting from insufficiencies or inadequacies in the instructions for use, the deployment, the installation, the operation, or any malfunction of the investigational medical software. This also includes any event that is a result of a use error or intentional misuse.

Treatment Emergent ADEs (TE-ADE) indicate ADEs to be collected at the point of exposure to the treatment and time thereafter.

8.1.2 Unanticipated Adverse Device Effects (UADEs)

A Unanticipated Adverse Device Effect is any serious adverse effect on health or safety or any life-threatening problem or death caused by, or associated with, a device, if that effect, problem, or death was not previously identified in nature, severity, or degree of incidence in the investigational plan or application (including a supplementary plan or application), or any other unanticipated serious problem associated with a device that relates to the rights, safety, or welfare of participants.

Unanticipated Adverse Device Effects will include events meeting <u>A and B</u> as stated below:

A. Events meeting **<u>ALL</u>** the following criteria:
   - Not included in the list of Anticipated Events (previously noted ADEs)
   - Possibly, probably, or definitely related to the investigational device per the PI
B. Serious (meets **<u>ANY</u>** of the following criteria):
   - Is a life-threatening illness or injury
   - Requires inpatient or prolonged hospitalization
   - Results in permanent (i.e., irreversible impairment or damage to a body structure or function, excluding trivial impairment or damage) impairment of a body structure
   - Necessitates medical or surgical intervention to prevent permanent impairment of a body function or a body structure
   - Led to fetal distress, fetal death or a congenital abnormality or birth defect
   - Led to death

8.2 Previously Noted Adverse Device (Software) Effects

Adverse Device (Software) Effects expected for **AKL-T01**:

1) Dizziness
2) Nausea
3) Headache
4) Decreased frustration tolerance
5) Emotional reaction
6) Aggression

The expectedness of an ADE shall be documented in the informed consent form. Any ADE that is not identified in nature, severity, or is not listed above is considered unanticipated.

8.3 Collection of Adverse Device (Software) Effects

A TE-ADE observed from the time of Intervention Training at the Baseline visit through the Exit visit will be collected in the study database.

At each study visit, the investigator will inquire about the occurrence of TE-ADEs since the last visit. TE-ADEs may be reported via participant communication with the study team during the at-home treatment phase or interviews of a study participant presenting for medical care. Study monitors may identify a TE-ADE upon review of subject source documentation.

All TE-ADEs including local and systemic reactions will be captured on the appropriate case report form (CRF). Information to be collected includes the event description, time of onset, relationship to study product (assessed only by those with the training and authority to make a diagnosis), clinician's assessment of severity, and time of resolution/stabilization of the event.

All TE-ADEs occurring while in the study will be documented appropriately regardless of relationship.

Any medical condition that is present at the time that the participant is screened will be considered as baseline and not reported as an ADE. However, if the study participant's condition deteriorates at any time during the study, it will be recorded as a TE-ADE.

Changes in the severity of a TE-ADE will be documented and presented by maximum severity. TE-ADEs characterized as intermittent require documentation of onset for each episode.

Events will be followed for outcome information until resolution or stabilization or until the final study visit.

8.4 Assessment of Adverse Device (Software) Effect

8.4.1 Relationship to Study Intervention

All TE-ADEs must have their relationship to study treatment assessed by the clinician who examines and evaluates the participant based on temporal relationship and his/her clinical judgment. In a clinical trial, the investigational device must always be suspect. The degree of certainty of causal relationship of an adverse device effect of either study treatment will be rated as follows:

- **Possible:** An event that might be due to the use of the study application. An alternative explanation - e.g., concomitant drug(s), concomitant disease(s) - is inconclusive. The relationship in time is reasonable; therefore, the causal relationship cannot be excluded.
- **Probable:** An event that might be due to the use of the study application. An alternative explanation is less likely - e.g., concomitant drug(s), concomitant disease(s). The relationship in time is suggestive.
- **Definite:** An event that is due to the use of the study application. The event cannot be reasonably explained by an alternative explanation - e.g., concomitant drug(s), concomitant disease(s).

8.4.2 Severity of Adverse Device (Software) Effects

The severity of a TE-ADE will be rated as follows:

- **Mild:** Awareness of sign, symptom, or event, but easily tolerated.
- **Moderate:** Discomfort enough to cause interference with usual activity and may warrant intervention.
- **Severe:** Incapacitating with inability to do usual activities or significantly affects clinical status and warrants intervention.

8.4.3 Outcome of Unanticipated Adverse Device (Software) Effects

If the Sponsor determines that an UADE presents an unreasonable risk to participants, all investigations or parts of investigations presenting that risk shall be terminated as soon as possible. Termination shall occur no later than 5 working days after the Sponsor makes this determination and no later than 15 working days after the first received notice of the effect.

8.5 Reporting of Adverse Device (Software) Effects

It is understood that complete information about an event may not be known at the time the initial report is submitted. The investigator must assess the relationship of the event to the investigational device/software (including rationale for assessment) and should make every attempt to obtain as much information as possible concerning the event.

Additional information pertaining to an event should be reported in the clinical database as it becomes available.

8.5.1 Site Reporting

Within 24 hours of knowledge of the event, the investigator must report all related TE-ADEs, anticipated or unanticipated, occurring from the time of study Intervention Training, Baseline Day 1, in the clinical database.

The investigator must document when important follow-up information (final diagnosis, outcome, results of specific investigations, etc.) becomes available after documentation of the initial Suspected Adverse Reaction information.

Follow-up information should be collected according to the same process used for documenting the initial event as described above.

8.5.2 Institutional Review Board Reporting

The investigator will submit all related unanticipated, and serious TE-ADEs, to the reviewing Institutional Review Board (IRB) according to IRB policies and procedure.

8.5.3 Regulatory Reporting

A sponsor who conducts an evaluation of an UADE under 21 CFR 812.46(b) shall report the results of such evaluation to the Food and Drug Administration (FDA) and to all reviewing IRBs and participating investigators within 10 business days after the sponsor first receives notice of the effect.

Thereafter, the sponsor shall submit such additional reports concerning the effect as FDA requests.

9. DATA AND SAFETY MONITORING

9.1 Study Data Collection and Data Entry

Clinical data is any data point that is captured outside of the AKL-T01 gameplay data.

Clinical data for this study will be captured in an electronic format. Each participant will be assigned a series of electronic Case Report Forms (eCRFs) that will be contained in the EDC. Details of oversight and management of the eCRFs/EDC will be detailed in the study Data Management Plan.

The investigator must ensure that all staff involved in the conduct of the trial are familiar with the protocol and all study-specific procedures, and that they have appropriate knowledge of the study agents. The investigator, or personnel delegated by the investigator, will perform primary data collection based on the protocol design and assessments performed. Site staff will perform data entry into the EDC based on the data captured in the original source documentation.

This study will use a study specific eCRF to capture all clinical data. All required study information must be recorded on the appropriate eCRF screen/form using the eCRF Completion Guidelines for the study. A CRF must be completed for each participant.

All data must be carefully entered in a timely fashion to permit meaningful interpretation and study oversight. Required clinical data will be entered in the eCRF as soon as possible after collection, and no later than 3 business days after the completed visit.

The investigator is responsible for ensuring that the clinical data is entered into the eCRF appropriately. The investigator is responsible for signing off on the final eCRF prior to locking.

AKL-T01 gameplay data will be collected on the iPad and automatically transferred to Akili upon connection of the iPad to the internet.

9.2 Study Dataset

The full study dataset will be collected for participants who enter the study by receiving study Intervention Training (AKL-T01).

Limited data (i.e., demographics, adverse events, selection criteria, and reason for discontinuation) will be collected for participants who screen fail or discontinue before the treatment phase.

All required data for this study will be entered into the eCRF.

9.3 Endpoint Determination

Data from the TOVA assessment will be derived directly from the computers used to administer the task and these data will be shared with Akili for analysis of primary outcome. Data from all other assessments will be collected by study personnel.

9.4 Safety Monitoring

Safety/tolerability will be assessed throughout the study.

9.5 Clinical Monitoring and Quality Control (QC)

Each clinical site will perform internal quality management of study conduct and data collection, documentation, and completion per site standard operating procedures.

The investigational site will provide direct access to all trial-related source data/documents, and reports for the purpose of monitoring and auditing by the sponsor and inspection by local and regulatory authorities.

A study Clinical Management Plan will be developed to include details regarding QC and monitoring of the clinical data. Oversight and management of the clinical data will be detailed in the study Data Management Plan.

Source data verification procedures will be implemented beginning with the data entry system, and data QC checks on the database will be generated. Sites will be queried on any missing data or data anomalies for clarification/resolution.

The study monitor will conduct risk-based source data verification (SDV) as detailed in the Clinical Monitoring Plan to ensure maximum data integrity.

9.6 Future Use of Stored Data

Data collected for this study will be analyzed and stored at Akili. After the study is completed, the coded, archived data will be transmitted to and stored at Akili and may be used for additional post hoc analyses.

10. STATISTICAL CONSIDERATIONS

This section provides a brief overview of the statistical analyses, both quantitative and qualitative in nature, that will be used during this study. In addition to what follows in this section, further details will be provided in a separate statistical analysis plan (SAP).

This study will use an adaptive design based on the total information[16] as measured by the standard error (SE) of the primary endpoint which will potentially allow the trial to be stopped prior to recruitment of the 325 participants derived from the sample size calculations described in Section 10.4. Given that there is uncertainty regarding whether the variation in TOVA-ACS mean change differs between the adult and pediatric ADHD populations, it is possible that the study could achieve a statistically but not clinically significant change in TOVA-ACS if the target sample size is enrolled. Therefore, the SE of mean change in TOVA-ACS will be estimated after the first 100 participants complete the study and again after every 25 additional participants complete the study until the SE is <= 0.277 to maintain 90% power to detect an effect size of 0.9. At this time, enrollment will end, and the study will conclude when all currently enrolled participants complete the study. No efficacy-related decisions will be made based on these interim looks (up to 10) unless the p-value for treatment effect is <0.000001. Therefore, the statistical penalty for multiple early looks at the data is negligible, and the final analysis of the primary efficacy endpoint will be conducted at the 0.025 significance level as originally planned.

- The primary objective of this study is to evaluate the efficacy of AKL-T01 as determined by the change in TOVA-ACS, a digitally assessed measure of sustained and selective attention, after 6 weeks of treatment with AKL-T01
- A secondary objective of this study is to evaluate the change in ADHD symptoms, as determined by change in the ADHD-RS-IV inattention sub-scale and total scale scores, after 6 weeks of treatment with AKL-T01
- Exploratory objectives of this study are:
    - To evaluate change in the AAQoL after 6 weeks of treatment with AKL-T01
    - To evaluate change in the CAARS-S:S after 6 weeks of treatment with AKL-T01
    - To evaluate response rates for TOVA-ACS and ADHD-RS-IV after 6 weeks of treatment with AKL-T01
    - To evaluate changes in TOVA metrics other than ACS after 6 weeks of treatment with AKL-T01

10.1 Endpoints

- The primary endpoint of this study is change (Study Day 1 to Study Day 42) on the TOVA-ACS.

- The secondary endpoints of this study are change (Study Day 1 to Study Day 42) in the administered ADHD-RS-IV inattention scale and total scale scores.
- Exploratory endpoints of this study include
    - Change (Study Day 1 to Study Day 42) in AAQoL total score and the Life Productivity subscale score
    - Change (Study Day 1 to Study Day 42) in the CAARS-S:S score
    - Proportion of responders at end of treatment defined as
        - ≥ 30% reduction in total ADHD-RS-IV score[12]
        - 8-point change in AAQoL[15]
    - Change (Study Day 1 to Study Day 42) in TOVA metrics other than ACS
        - Ex-Gaussian Tau Total
        - Commission Errors Standard Score H2
        - RT Variability Standard Score Total
        - RT Mean Standard Score H1
        - D-Prime Standard Score H2
        - Omission Errors Standard Score H2

10.2 Analysis Populations

Efficacy Population

The efficacy population will consist of any participant that is sent home with the AKL-T01 intervention that (for the purpose of this protocol):

1. Completes baseline assessments
2. Completes their exit assessments

Per Protocol (PP) Population

The PP population will be a subset of the efficacy population that meets definition for "adequate dosage" or "minimum acceptable exposure" to the game which will be defined as by completion of at least 60% of prescribed missions, equivalent to 108 of 180 missions (at least 6 missions/day × 5 days/week × 6 weeks) will be used in sensitivity analyses. Patients with any protocol deviations that could impact efficacy will be excluded from the PP population.

Safety Population

The safety population will consist of all participants who are exposed to AKL-T01 intervention.

10.3 Statistical Analysis

Full details of all statistical analyses will be provided in a separate statistical analysis plan. Analyses will be conducted using a complete case analysis for participants with sufficient data at baseline and follow-up timepoints. Missing data will not be imputed.

Baseline characteristics (socio-demographic, medical history, ADHD history, ADHD treatments, etc.) of the study population will be described using mean and standard deviation as well as median, minimum, maximum and interquartile range, as appropriate for continuous variables, and counts with percentages for categorical variables. Compliance metrics will also be reported descriptively in the efficacy population using the same conventions.

10.3.1 Analysis of the Primary Endpoint

The primary endpoint is the change in TOVA-ACS calculated as the score at Day 42 minus the score at baseline for each participant. Since no missing data will be imputed, any patients without TOVA-ACS measurements at either timepoint will be excluded from the analysis. The primary analysis population will be the efficacy population, with analyses repeated for the PP populations as sensitivity analyses.

Descriptive statistics for TOVA-ACS baseline, follow-up and change scores will be calculated (mean, standard deviation, median, minimum, maximum, interquartile range) for patients with complete data at both timepoints. The null (H_0) and alternative (H_a) hypotheses for assessing mean change (μ_d) in baseline to Day 42 measurements of TOVA-ACS are:

$$H_0\text{: } \mu_d = 0$$
$$H_a\text{: } \mu_d \neq 0$$

A two-sided one-sample *t*-test will be used to evaluate the mean change in TOVA-ACS with significance level of $\alpha = 0.05$. A statistically significant improvement will be defined by a 95% confidence interval for the mean change with lower bound greater than zero and corresponding *p*-value < 0.05. Given the large target sample size for this study, the *t*-test should be valid for assessing the change in TOVA-ACS relative to zero per the Central Limit Theorem even if the data deviates from a Normal distribution.[16] Distribution of the change scores and use of equivalent nonparametric tests (i.e., Wilcoxon signed rank test) may be explored by visually assessing appropriate diagnostic plots (e.g., histogram, Q-Q plot) at the discretion of the investigators.

10.3.2 Analysis of Secondary Endpoints

Secondary endpoints for this study are change in ADHD-RS-IV (1) inattention subscale score, and (2) total scale score, calculated as the score at Day 42 minus the score at

baseline. Analysis of secondary endpoints will follow the methods described above for the primary endpoint with mean changes in respective ADHD-RS-IV scores considered here as μ_d. A lower score on these measures indicates less severe symptoms, so a 95% confidence interval with upper bound less than zero will be considered a statistically significant improvement in these analyses.

Adjustment for multiplicity will be conducted for the primary and two secondary endpoints. Specific methods will be described in the SAP.

10.3.3 Analysis of Exploratory Endpoints

Exploratory endpoints for this study include change in (1) AAQoL total score, and (2) AAQoL Life Productivity subscale score, (3) CAARS-S:S score, and (4) specified TOVA metrics other than ACS. All differences will be calculated as the score at Day 42 minus the score at baseline and analyzed using the same methods as for the primary and secondary outcomes. Type I error will not be controlled for in analyses for exploratory endpoints, and only nominal *p*-values will be reported. Analyses will be conducted on the efficacy and repeated in the PP populations.

For responder analyses, patients achieving $\geq$ 30% reduction in total ADHD-RS-IV score and 8-point change in AAQoL will be reported descriptively using counts and percentages. Additional responder analyses may be considered and will be described in the SAP if incorporated in the study.

10.3.4 Subgroup Analyses

Summary statistics for all efficacy endpoints will be provided for the following subgroups as additional exploratory analyses:

- Concomitant stimulant medication use: yes, no.
- Alternate definition(s) of treatment adherence, with categories to be determined post hoc

10.4 Sample Size

Sample sizes were calculated to test the null hypothesis of a zero change in TOVA-ACS from baseline to Study Day 42 against the alternative of a positive change in TOVA-ACS over the same period with a one-sample paired t-test using SAS® version 9.4 (SAS Institute, Cary, North Carolina). The significance level was defined by a one-sided α=0.025 with a minimum power of 90% to detect a mean difference in pre- and post-intervention TOVA-ACS.

Given that the adult ADHD population is expected to be more heterogeneous than the pediatric ADHD population, it is assumed that the standard deviation σ in TOVA-ACS in this study population is 1.5 times that observed with TOVA-API in the study population of STARS-ADHD where SD=3.2. Assuming the previously observed control-corrected effect size of $\mu=0.9$ and 1.5 times the observed standard deviation of $\sigma=4.8$, a sample size of 301 participants is required to detect the effect with 90% power.

10.5 Safety Analysis

Treatment related adverse events for the AKL-T01 intervention will be presented. Any adverse events occurring during any phase of the study judged by the PI to be related to the intervention will be recorded and presented in a table. The Safety Population will be used for this analysis that will include participants that began the at-home treatment phase for the intervention. The severity of each event will be evaluated by the PI and presented with the recorded events. If there are frequently occurring treatment related adverse events, a frequency table will be included, to rank events from most frequent to most rare.

11. OPERATIONAL CONSIDERATIONS

11.1 Informed Consent

11.1.1 Consent/Assent Requirements

Informed consent will be obtained from all participants. Informed consent is a process that is initiated before the individual agrees to participate in the study and continues throughout the individual’s study participation. Consent forms will be Institutional Review Board (IRB) approved, and the participants will be asked to read and review the document.

The informed consent process will be conducted and documented in the source document (including the date), and the form signed before the participant undergoes any study-specific procedures. Participants must be informed that participation is voluntary and that they may withdraw from the study at any time, without prejudice. The rights and welfare of the participants will be protected by emphasizing to them that the quality of their medical care will not be adversely affected if they decline to participate in this study.

Consent forms describing in detail the study treatment, study procedures, and risks are given to the participants and written documentation of informed consent is required before starting treatment/administering study treatment.

11.1.2 Other Information and Materials Provided to Participants

Any other study related informational materials provided to participants will be submitted for review and approval to the reviewing IRB according to IRB policies and procedure.

11.2 Confidentiality and Privacy

Participant confidentiality and privacy is strictly held in trust by the participating investigators, their staff, and the sponsor. The study protocol, documentation, data, and all other information generated will be held in strict confidence. No information concerning the study, or the data will be released to any unauthorized third party without prior written approval of the sponsor.

The study monitor, other authorized representatives of the sponsor, representatives of the IRB, and regulatory agencies may inspect all documents and records required to be maintained by the investigator, including but not limited to, medical records (office, clinic, or hospital) and pharmacy records for the participants in this study. The clinical study site will permit access to such records.

The study participants' contact information will be securely stored at each clinical site for internal use during the study. At the end of the study, all records will continue to be kept in a secure location for as long a period as dictated by the reviewing IRB, Institutional policies, or sponsor requirements.

Study participant research data, which is for purposes of statistical analysis and scientific reporting, will be transmitted to and stored at Akili. This will not include the participant's contact or identifying information. Rather, individual participants and their research data will be identified by a unique study identification number. The study data entry and study management systems used by clinical sites and by Akili Interactive's research staff will be secured and password protected. At the end of the study, all study databases will be coded and archived at Akili.

11.3 Trial Master File and Records Retention

The Trial Master File (TMF) is the collection of study documentation consisting of essential documents, which enable both the conduct of the clinical trial and the quality of the data produced to be evaluated.

Akili Clinical Operations is responsible for ensuring that complete and accurate documentation is prepared and maintained in compliance with good documentation practices and applicable federal, state, and local laws, rules and regulations.

TMF documentation includes all CRFs, regulatory documents and other study related documents (e.g., signed protocol and amendments, IRB correspondence and approval, approved participant consent forms, signed Investigator Statement form, clinical supplies receipts and distribution records).

TMF Documentation for this study will be maintained electronically in an electronic Trial Master File system (eTMF). Details of the oversight and management of the study TMF is detailed in the Trial Master File Management Plan.

The PI is responsible for filing of site essential documentation in the Investigator Site File. The PI will ensure all study documentation from the site will be available to Akili Clinical Operations and contracted vendors for review and monitoring.

Clinical operations and participating sites maintain all study documentation from the initiation of the study through 2 years after the latter of the following: either the date the study is complete or the date the records are no longer required to meet a regulatory approval application.

11.4 Protocol Deviations

A protocol deviation is defined as an event where the investigator or study personnel did not conduct the study according to the investigational plan.

All protocol deviations will be documented in the protocol deviation CRF. The IRB will be informed by the investigator of all protocol changes or deviations that occur in accordance with applicable regulations and the IRB's established procedures. No deviations from the protocol of any type will be made without complying with all the IRB/EC's established procedures.

11.5 Study Discontinuation and Closure

This study may be temporarily suspended or prematurely terminated if there is sufficient reasonable cause. Written notification, documenting the reason for study suspension or termination, will be provided by the sponsor to regulatory authorities, investigators, and study participants as applicable. If the study is prematurely terminated or suspended, the site principal investigator (PI) will promptly inform study participants, their reviewing IRB, and will provide the reasons for the termination or

suspension. Study participants will be contacted, as applicable, and be informed of changes to study visit schedule.

Circumstances that may warrant termination or suspension include, but are not limited to:

- Determination of unexpected, significant, or unacceptable risk to participants
- Demonstration of efficacy that would warrant stopping
- Insufficient compliance with protocol requirements
- Data that are not sufficiently complete and/or evaluable
- Determination that the primary endpoint has been met
- Determination of futility

Study may resume once concerns about safety, protocol compliance, and data quality are addressed, and satisfy the sponsor, IRB and/or Food and Drug Administration (FDA).

11.6 Audits and Inspections

The investigator will provide access to all study related documents and trial supplies for the purpose of sponsor oversight and quality assurance or inspection by local and federal regulatory authorities.

11.7 Conflict-of-Interest Policy

Any actual conflict of interest of persons who have a role in the design, conduct, analysis, publication, or any aspect of this study will be disclosed and managed. Furthermore, persons who have a perceived conflict of interest will be required to have such conflicts managed in a way that is appropriate to their participation in the design and conduct of this study.

12. REFERENCES

1. Barkley RA, Fischer M, Smallish L, Fletcher K. The persistence of attention-deficit/hyperactivity disorder into young adulthood as a function of reporting source and definition of disorder. *J Abnorm Psychol*. 2002;111(2):279-289.

2. Kolar D, Keller A, Golfinopoulos M, Cumyn L, Syer C, Hechtman L. Treatment of adults with attention-deficit/hyperactivity disorder. *Neuropsychiatr Dis Treat*. 2008;4(1):107-121.

3. Compton WM, Han B, Blanco C, Johnson K, Jones CM. Prevalence and correlates of prescription stimulant use, misuse, use disorders, and motivations for misuse among adults in the U.S. *Am J Psychiatry*. 2018;175(8):741-755. doi:10.1176/appi.ajp.2018.17091048

4. Biederman J, Chan J, Spencer TJ, et al. Evidence of A Pharmacological Dissociation Between The Robust Effects of Methylphenidate on Adhd Symptoms and Weaker Effects on Working Memory. *J Brain Sci*. 2015;1(2):43-53.

5. Klingberg T. Training and plasticity of working memory. *Trends Cogn Sci*. 2010;14(7):317-324. doi:10.1016/j.tics.2010.05.002

6. Kollins SH, DeLoss DJ, Cañadas E, et al. A novel digital intervention for actively reducing severity of paediatric ADHD (STARS-ADHD): a randomised controlled trial. *Lancet Digit Health*. 2020;2(4):e168-e178. doi:10.1016/S2589-7500(20)30017-0

7. Kollins SH, Childress A, Heusser AC, Lutz J. Effectiveness of a digital therapeutic as adjunct to treatment with medication in pediatric ADHD. *Npj Digit Med*. 2021;4(1):58. doi:10.1038/s41746-021-00429-0

8. Anguera JA, Brandes-Aitken AN, Rolle CE, et al. Characterizing cognitive control abilities in children with 16p11.2 deletion using adaptive 'video game' technology: a pilot study. *Transl Psychiatry*. 2016;6(9):e893-e893. doi:10.1038/tp.2016.178

9. Yerys BE, Bertollo JR, Kenworthy L, et al. Brief Report: Pilot Study of a Novel Interactive Digital Treatment to Improve Cognitive Control in Children with Autism Spectrum Disorder and Co-occurring ADHD Symptoms. *J Autism Dev Disord*. 2019;49(4):1727-1737. doi:10.1007/s10803-018-3856-7

10. Hughes S. A Guide to Using the Test of Variables of Attention (T. :4.

11. DuPaul GJ, Power TJ, Anastopoulos AD, Reid R. *ADHD Rating Scale–IV: Checklists, Norms, and Clinical Interpretation.* Guilford Press; 1198.

12. Goodman D, Faraone SV, Adler LA, Dirks B, Weisler R. Interpreting ADHD Rating Scale Scores: Linking ADHD Rating Scale Scores and CGI Levels in Two Randomized Controlled Trials of Lisdexamfetamine Dimesylate in ADHD. *Prim Psychiatry*. Published online 2010:9.

13. Brod M, Johnston J, Able S, Swindle R. Validation of the adult attention-deficit/hyperactivity disorder quality-of-life Scale (AAQoL): a disease-specific quality-of-life measure. *Qual Life Res Int J Qual Life Asp Treat Care Rehabil*. 2006;15(1):117-129. doi:10.1007/s11136-005-8325-z

14. Conners CK, Erhardt D, Sparrow EP. *Conners' Adult ADHD Rating Scales (CAARS): Technical Manual*. Multi-Health Systems North Tonawanda, NY; 1999.

15. Tanaka Y, Brod M, Lane JR, Upadhyaya H. What Is a Clinically Relevant Improvement in Quality of Life in Adults With ADHD? *J Atten Disord*. 2019;23(1):65-75. doi:10.1177/1087054715580395

16. US Food & Drug Administration. Adaptive designs for medical device clinical studies: guidance for industry and Food and Drug Administration staff.2016. https://www.fda.gov/downloads/medicaldevices/deviceregulationandguidance/guidancedocuments/ucm446729.pdf. Accessed 8 Oct 2021.
