# Supplementary material for: Secondary analyses of sex differences in attention improvements across three clinical trials of a digital therapeutic in children, adolescents, and adults with ADHD
Source: BMC Public Health. 2024 Apr 29;24:1195. doi: 10.1186/s12889-024-18597-5 (PMC11057090; doi:10.1186/s12889-024-18597-5)
Supplement: Supplementary file 1 — Supplementary Material 1. [file 12889_2024_18597_MOESM1_ESM.zip › AKL-T01 Pediatric Study Protocol.pdf]

akili

Akili-001R

Version 1.02

A randomized, controlled parallel-group, intervention study to assess at-home, game-based digital therapy for treating pediatric participants ages 8 to 12 years old with Attention Deficit Hyperactivity Disorder (ADHD)

Date: August 4, 16

Version: 1.02

Principal Investigator:

Scott H. Kollins, Ph.D.

Medical Monitor:

Andrew Krystal, M.D.

Akili Interactive Labs, Inc.

PROTOCOL APPROVAL PAGE

Study Title: A randomized, controlled parallel-group, intervention study to assess at-home game-based digital therapy for treating pediatric participants ages 8 to 12 years old with Attention Deficit Hyperactivity Disorder (ADHD).

Version: 1.02

Date of Issue: August 4, 16

Study Sponsor: Akili Interactive Labs, Inc.
125 Broad St., 4th Floor
Boston, MA 02110

We, the undersigned, have read and approve this protocol and agree on its content.

[signature]
Sponsor Representative

8/4/16
Date

[signature]
Principal Investigator

8/4/16
Date

PROTOCOL VERSION AND AMENDMENT TRACKING

Version Number/Amendment	Approval Date
1.00	11/17/15
1.01/ Amendment 1	04/06/16
1.02/Amendment 2	07/25/16

Summary of Changes

Amendment 2:

1) Updated information regarding participant's ability to access 3rd party applications, download additional applications, and store personal information.
2) Added requirement for sites to hold and document a discussion with each participant to describe intended and unintended use of the device, as well as study restrictions and requirements.
3) Added requirement for sites to return the device to Akili for complete erasing of all the data entered prior to re-circulating the device.
4) Updated description of the secondary and descriptive endpoints to be consistent with the current SAP
5) Added detail on administering the IRS

.

Amendment 1:

1) Updated Akili business address to reflect current location
2) Attention Performance Index (API) is an output of TOVA <u>version 8</u>. Specified "TOVA 8" where TOVA is mentioned in conjunction with API. Also added clarity that API is a "composite measure"
3) Added analysis of TOVA non-composite measures to the secondary endpoints
4) Added exclusion criteria that potential subjects could not have previously participated in any assessment of Akili's videogame-like digital therapy
5) Clarified the study design that the Investigators and Outcome Assessors are blinded to the treatment assignment.
6) Added analysis of responders and descriptive analysis as exploratory endpoints
7) Timing of interim analysis update to reflect N = 75/75
8) Clarified that maximum enrolment, following interim analysis, is 1000 subjects
9) Added a baseline visit expectation survey for the parents to complete
10) To better preserve the blinding, removed the "A" / "B" labeling of the iPads (previously corresponding to treatment assignment) and included an anonymous serial number. A separate blinded treatment assignment list will be generated
11) Clarified instructions for connecting via remote Wi-Fi locations
12) Updated the statistical analysis section to reference details that are described in a separate statistical analysis plan (SAP) and added a non-inferiority test to the primary objective
13) Clarified subject replacement criteria to include subjects that don't complete the in-clinic exit assessments

14) Require participants to be able to spell at least 2 words during EVO Words assessment
15) Added requirement where same study staff administers the ADHD-RS, CGI, and IRS pre/post visits
16) Capture any unblinding event in the CRF

PROTOCOL SYNOPSIS

Name of Company	Akili Interactive Labs, Inc., 125 Broad Street, 4th Floor, Boston, MA 02110
Study Product	"EVO", Multi-tasking Game-Based Digital Therapy or "EVO" Words Game-Based Digital Therapy
Protocol Number	Akili-001R
Protocol Title	A randomized, controlled parallel-group, intervention study to assess at-home, game-based digital therapy for treating pediatric participants ages 8 to 12 years old with Attention Deficit Hyperactivity Disorder (ADHD)
Main Criteria for Inclusion	Inclusion<br>• Age 8 years to 12 years, inclusive, at the time of parental informed consent<br>• Male or female<br>• Confirmed ADHD diagnosis, any presentation, at Screening based on DSM-V criteria and established via the MINI-KID administered by a trained clinician<br>• Screening/Baseline (Visit 1 or 1a) score on the clinician-rated ADHD-RS-IV score $\geq$ 28<br>• Screening/Baseline (Visit 1 or 1a) score on the TOVA 8 API $\leq$ -1.8<br>• Not undergoing pharmacological treatment with methylphenidate or amphetamine-based products at time of Screening; or, if undergoing pharmacological treatment, must be willing and appropriate (i.e. not optimally treated in the investigator's judgment) to wash out of current regimen.<br>• Ability to follow written and verbal instructions (English), as assessed by the PI and/or study coordinator<br>• Estimated IQ score $\geq$ 80 as assessed by the Kaufmann Brief Intelligence Test, Second Edition (KBIT-II)

	• Ability to comply with all the testing and requirements.<br>Exclusion<br>• Current, controlled (requiring a restricted medication) or uncontrolled, comorbid psychiatric diagnosis , based on MINI-KID and subsequent clinical interviewing, with significant symptoms including but not limited to post-traumatic stress disorder, psychosis, bipolar illness, pervasive developmental disorder, severe obsessive compulsive disorder, severe depressive or severe anxiety disorder, conduct disorder, or other symptomatic manifestations that in the opinion of the Investigator that may confound study data/assessments. Participants with clinical history of learning disorders will be allowed to participate, provided the disorder does not impact their ability to participate in the trial based on PI judgment<br>• Participants who are currently treated with a non-stimulant medication for ADHD (i.e., atomoxetine, clonidine, guanfacine)<br>• Initiation within the last 4 weeks of behavioral therapy. Participants who have been in behavior therapy consistently for more than 4 weeks may participate provided their routine is unchanged during the course of the study. Participants planning on changing or initiating behavior therapy during the course of the study will be excluded.<br>• Participant is currently considered a suicide risk in the opinion of the Investigator, has previously made a suicide attempt, or has a prior history of, or is currently demonstrating active suicidal ideation or self-injurious behavior as measured by C-SSRS at screening.

	• Motor condition (e.g., physical deformity of the hands/arms; prostheses) that prevents game playing as reported by the parent or observed by the investigator.<br>• Recent history (within the past 6 months) of suspected substance abuse or dependence.<br>• History of seizures (exclusive of febrile seizures), or significant motor or vocal tics, including but not limited to Tourette's Disorder.<br>• Has participated in a clinical trial within 90 days prior to screening.<br>• Diagnosis of or parent-reported color blindness (Confirmed in-clinic via ICBT)<br>• Uncorrected visual acuity (Confirmed in-clinic via ability of subject to play the game)<br>• Regular use of psychoactive drugs (other than stimulant) that in the opinion of the Investigator may confound study data/assessments.<br>• Any other medical condition that in the opinion of the investigator may confound study data/assessments.<br>• Has a sibling also enrolled/currently participating in the same study<br>• Has previously participated in a study of Akili's videogame-like digital therapy<br><br><u>Concomitant Medication and Treatment</u><br>• With the exception of common over the counter (OTC) (e.g. ibuprofen, acetaminophen) and prescription medications (e.g. antibiotics) for minor transient ailments, regular use of concomitant medications (i.e., sleep aids, etc.) are not permitted during the study. Transient use of some medications (i.e., non-sedating antihistamines to treat seasonal allergies) may be permitted on a case-

	by-case basis with PI and medical monitor review/approval. Families should advise the study staff if it becomes necessary to use other types of medication. Study investigators can approve short-term use of other medications that are not anticipated to confound study assessments.<br>• Initiation of or significant changes in non-pharmacological treatment for ADHD is not permitted during the course of the study. Participants actively engaged in non-pharmacological treatment at Screening, may be eligible if they meet all inclusion criteria and do not undergo changes to their treatment during the course of the study.
Study Objective	To evaluate the effects of EVO Multitasking digital therapy versus EVO Words digital therapy on attentional functioning (measured by the TOVA) and symptoms in children diagnosed with ADHD
Study Design	Blinded (Investigator / Outcome Assessor), Randomized, Parallel-group
Treatment Regimen	25 minutes/day for 5 days/week of either EVO multitasking digital therapy or EVO Words digital therapy across the 4-week (28 Day) treatment period.
Duration of Study Participation	36 days
Number of Participants	A minimum of 300 to a maximum of 1,000 participants will be enrolled to achieve a minimum of 300 to a maximum 900 completed participants (n=150 - 450 in each of 2 arms) evaluable
Number of Sites	6-9
Primary Endpoint	Change (from Study Day 0 to Study Day 28) on the Attention Performance Index (API), an overall composite score, from the Test of Variables of Attention (TOVA 8)

Secondary Endpoints	Change (from Study Day 0 to Study Day 28) in:<br>• ADHD Symptoms as reported on the ADHD-RS total score, ADHD-RS Inattentive Sub-scale, ADHD-RS hyperactivity sub-scale, BRIEF working memory percentile, BRIEF inhibit percentile, Impairment Rating Scale, and Clinical Global Impression Scale.<br>• Objective Working Memory as measured by the CANTAB spatial working memory test<br>• Objective Inhibition as measured by the TOVA commission error standard score in half-2.
Exploratory/Descriptive Endpoints	Analysis includes:<br>• Responder analysis, percentage of responders to primary and secondary endpoints<br>• Descriptive analysis of child/parent surveys, treatment regimen compliance, demographics, missing data, site poolability, and any resulting treatment related AEs<br>• Analysis of secondary symptom and executive function endpoints adjusted for baseline parent expectations
Interim Analyses	An interim analysis will be conducted at N =75 / 75 (EVO Multitask / EVO Words) to confirm powering and to finalize sample size

INVESTIGATOR STATEMENT

I have read the protocol, including all appendices and I agree that it contains all necessary details for me and my staff to conduct this study as described. I will personally oversee the conduct of this study as outlined herein and will make a reasonable effort to complete the study within the time designated.

I will provide all study personnel under my supervision with copies of the protocol and access to all information provided by Akili Interactive Labs, Inc. [*Sponsor*]. I will discuss this material with them to ensure that they are fully informed about the efficacy and safety parameters and the conduct of the study in general. I am aware that, before beginning this study, the institutional review board responsible for such matters must approve this protocol in the clinical facility where it will be conducted. I agree to make all reasonable efforts to adhere to the attached protocol.

I agree to provide all participants with informed consent forms, as required by government and International Conference on Harmonization regulations. I further agree to report to the sponsor any adverse experiences in accordance with the terms of this protocol and U.S. Food and Drug Administration regulation 21 Code of Federal Regulations 812.2(b).

____

Principal investigator Name (print)

____

Signature

____

Date

Contents

akili 1

Akili-001R 1

Version 1.01 1

Protocol Approval Page 2

Protocol Version and Amendment Tracking 3

Protocol Synopsis....................5

Investigator statement 10

1. Introduction 16
- 1.1 Background 16
- 1.2 Clinical Experience with Study Agent(s)....................16
- 1.2.2 Rationale....................19

2. Objectives 21
- 2.1 Primary Objective....................21
- 2.2 Secondary Objectives....................21
- 2.3 Exploratory & Descriptive Objectives 21

3. Subject Selection 22
- 3.1 Inclusion Criteria....................22
- 3.2 Exclusion Criteria 22
- 3.3 Concomitant Medications and Treatments....................23

4. Study Design....................25

4.1. overview of Study....................25
- 4.2 Treatment Arms....................26
- 4.3 Screening/Baseline Procedures 29
- 4.4 Washout Procedures & Screening/Baseline (Post Washout) Procedures....................32
- 4.5 Treatment Procedures – In-Home Game Play and Post-Treatment Visit....................34
- 4.6 Procedures to minimize bias and blinding to treatment assignment....................36
  - 4.6.1 Procedures to minimize bias in parents and participants. 37
  - 4.6.2 Procedures to blind/minimize bias in site staff and investigators. 37
  - 4.6.3 Procedures in the event of possible or probable unblinding. 38
- 4.7 Randomization Procedures....................38

5 Study Device....39
5.1 Study Device Usage....39
5.2 Potential Benefits/Risks of Study Device....41
5.3 Procedures for Monitoring Compliance and Replacing Participants....42
5.3 Discontinuation of Study Device....43

6 Concomitant Medications....44
6.1 Allowed Medications....44
6.2 Prohibited Medications....44

7.0 Adverse Device Effect Reporting and Follow-up....45
7.1 Definitions....45
Unanticipated Adverse Device Effects (UADEs)....45
7.2 Assessment....46
7.2.1Time Frame....46

7.2.2 Causality Rating....46
7.2.3 Severity of Adverse Device Effects....46
7.3 Previously Noted Adverse Device Effects....47
7.4 Reporting....47
7.4.1 Adverse Device Effect....46

7.4.2 Regulatory Reporting of Unanticipated Adverse Device Effects....48

7.5 Institutional Review Boards....47

8.0 Statistical Analysis e....48
8.1 Primary Objective (P1) -Attention....49
8.2 Secondary Objectives....50
8.2.1 Major Secondary Objective 1 (ST) ADHD Symptom. 50
8.2.2 Minor Secondary Objective II (S2) Objective Working Memory....50
8.2.3 Minor Secondary Objective III (s3) Objective Inhibition....51

8.3 Exploratory Objectives....51
8.3.1 Exploratory Objective 1 (E1) Responders....51
8.3.2 Exploratory Objective II (E2) Additional TOVA Responses....51
8.4 Safety Analysis....52
8.5 Descriptive Analyses....52

8.6 General Statistical Methods....52
8.6.1 Hypothesis Tests and Multiplicity Adjustments....52
8.6.2 Analysis Populations and Missing Data....53
8.6.3 Interim Analysis....53

8.6.4 Power and Sample Size 53

9. Data and Safety Monitoring 55
9.1 Endpoint Determination 55
9.2 Safety Monitoring 55
9.3 Data Management to Maintain Blinding 55

10 Data Monitoring and Quality Control 56
10.1 Required Data 56
10.2 Subject and Clinical Data 56

11 Study Responsibilites 57
11.1 Study Data Reporting and Processing 57
11.2 Training 57
11.3 Monitoring the Investigational Sites 57
11.4 Study Documentation 58
11.5 Protocol Deviations 58
11.6 Study Supply Accountability 59
11.7 Data Transmittal and Record Retention 59
11.8 Study Closeout 60
11.9 Audit/Inspections 60
12 References 61

Abbreviations

ADHD	Attention Deficit Hyperactivity Disorder
DSM-V	Diagnostic and Statistical Manual of Mental Health-Fifth Edition
MINI-KID	MINI International Neuropsychiatric Interview for Children and Adolescents
ADHD-RS-IV	Attention Deficit Hyperactivity Disorder Rating Scale
TOVA	Test of Variables of Attention
API	Attention Performance Index
KBIT	Kaufman Brief Intelligence Test
PI	Principal Investigator
C-SSRS	Columbia Suicide Severity Rating Scale
ICBT	Ishihara Color Blindness Test
OTC	Over the counter
CANTAB	Cambridge Neuropsychological Test Automated Battery
BRIEF	Behavior Rating Inventory of Executive Function
CGI-I	Clinical Global Impression-Improvement
IRS	Impairment Rating Scale
ASD	Autism Spectrum Disorder
LD	Learning Disability
CANTAB SWM	Cambridge Neuropsychological Test Automated Battery Spatial Working Memory
ICF	Informed Consent Form
DCRI	Duke Clinical Research Institute
SSRI	Selective Serotonin Reuptake Inhibitor
MAOI	Monoamine Oxidase Inhibitor
ADE	Adverse Device Effect
UADE	Unanticipated Adverse Device Effect
eCRF	Electronic Case Report Form
FDA	Food and Drug Administration
IRB	Institutional Review Board
CPT	Continuous Performance Test
ComSS	Commission Error Standard Score
ROC	Receiver Operator Curve
SAP	Statistical Analysis Plan
APP	Application

GCP	Good Clinical Practice
CMP	Clinical Monitoring Plan
HIPPA	Health Insurance Portability and Accountability Act

1. INTRODUCTION

1.1 Background

Front-line treatment for ADHD includes the use of stimulant medications, which is effective in reducing the core symptoms of the disorder but are associated with well-documented side effects. In addition, there is recent evidence that pharmacological treatment may not show optimal benefits in some cognitive domains. (Biederman, J, et al 2015). Computerized cognitive training programs have shown some promise to improve working memory and attention in ADHD populations. (Klinberg, T.)[Further research is needed to validate the effectiveness of currently available computerized cognitive interventions as well as for consumer-grade gamified therapies that are still being developed.

This protocol will evaluate the effects of a novel, highly-immersive, videogame-based interventions for ADHD *Project: EVO that* was derived from a comparable cognitive training program shown to have robust effects on working memory, attention, and related constructs in older adults.(Angora, J.A., et al) *EVO* Multitask incorporates adaptive, simultaneous cognitive tasks into a consumer-grade action video gaming-like platform with high-quality graphics and reward loops, and is deployed on mobile devices (i.e., tablets). We hypothesized that *EVO* Multitask digital therapy would be well-tolerated and feasible to administer to pediatric participants with ADHD, and would improve functioning in relevant cognitive domains. As a control, this protocol will also evaluate the effects of a highly-immersive educational videogame, "EVO Words" in a matched, time-on-task manner.

Novel, cost-effective, and easy to implement/disseminate non-pharmacological interventions for ADHD could be helpful for many participants given limitations of other approved interventions.

1.2 Clinical Experience with Study Agent(s)

Akili has evaluated the EVO Multitask digital therapy in various clinical populations as described below.

Most relevant to the current protocol, a multi-site, open-label, proof-of-concept study was recently completed in children with ADHD. In this study, ADHD (N = 40, Mean Age = 10.3 years) and age-matched neuro-typical control (N =40, Mean Age = 10.5 years) participants were recruited from 3 sites and screened for enrollment. A one month, at-home intervention followed, which

consisted of 5 weekly 30-minute sessions of digital therapy play. Participants then returned to the clinic for post-intervention assessment. Measures of cognitive function (TOVA, CANTAB) and behaviors (Parent-BRIEF) were collected at Screening and Post-Intervention. Tolerability, compliance, and acceptability were also assessed. No treatment related adverse events were reported. The ADHD group completed an average of 81% of the required at-home sessions, and reported a high level of enjoyment on average. The Attention Performance Index composite score from the TOVA improved significantly from pre-post intervention ($p = 0.033$, Cohen's $d = 0.35$) in the ADHD group. There was no change in the neuro-typical group ($p = 0.30$, Cohen's $d = 0.16$). A post-hoc sub-population analysis of a large group of ADHD participants with greater cognitive and symptom severity at Baseline (TOVA API <= -1.8 & ADHD-RS >=30) showed a larger effect of the intervention ($p = 0.003$, Cohen's $d = 0.71$). The CANTAB Spatial Working Memory test showed statistically significant improvements on 9 of its 19 reported measures for the ADHD group while the neuro-typical group improved on 3 of the 19 measures. For the Parent-BRIEF, the ADHD group showed improvements for working memory, however this measure did not meet the alpha criterion for multiple comparisons. No change was found for the Parent-BRIEF in the neuro-typical population. Results from this proof-of-concept study provide initial support for this novel, consumer-grade video game intervention as a potential treatment for attention and working memory symptoms in pediatric ADHD populations.

Additional studies using EVO Multitask in related populations include:

A feasibility study was conducted at a small college for individuals with ADHD and other related learning difficulties. Forty-four students with a diagnosis of ADHD, ASD, LD (or a combination thereof), were asked to play EVO Multitask in their dorm rooms for 30 minutes/day for 1 week. The study showed that EVO Multitask was generally engaging and the one week protocol was feasible.

General Results:

- Over the week, participants were able to complete, on average, 25% progress through the game (matching the expected pace of 100% progress through 4 weeks).
- The participants demonstrated an expected deficit in baseline game performance when playing in the multi-task environment as compared to single-task, confirming an interference processing deficit.

- Over the week protocol, there was significant improvement in subject reaction time, as measured in the EVO Multitask program.
- No treatment related adverse events were reported.

In a pilot study of pediatric sensory processing disorder at the University of California, San Francisco (UCSF), 45 children ages 6 to 14 (23 with sensory processing disorders / 22 age-matched neuro-typical children), were asked to play EVO Multitask at home for 30 minutes/day, 5 days a week, for 4 weeks. Both groups demonstrated that EVO was generally engaging and a 4-week at-home protocol in these populations was feasible.

General Results:

- Participants completed, on average, greater than 70% of the game sessions.
- Over the first ~1.5 weeks of at-home play, there was improvement (~70 – 150%) in reaction time, as measured in the EVO program.
- No treatment related adverse events were reported.

In a feasibility study at the Center for Autism Research Disorders (CARD-ABA Clinic), 10 children with **low-functioning autism** ages 4 to 14, were evaluated for their ability to engage with the EVO Multitask digital therapy during an in-clinic visit. Optionally they could take the game home for 1 week of at-home intervention play.

General Results:

- Younger participants with more severe ASD were unable to engage with EVO Multitask (the digital therapy mechanics and instruction were too difficult).
- Two of the older participants with somewhat less impairment took EVO Multitask home. These participants completed less than 25% of the game over the week, implying that at-home gameplay may not be feasible for this population.
- No treatment related adverse events were reported in any of the participants.

Additionally, Akili is currently conducting ongoing research in several additional clinical populations including geriatric depression, traumatic brain injury, Parkinson's disease, cerebrovascular disease, and prodromal Alzheimer's disease. To date no treatment related adverse events have been reported.

1.2.2 Rationale

ADHD is one of the most common pediatric psychiatric conditions and best practice for its treatment includes both pharmacological (usually psychostimulants – methylphenidate or amphetamine) and non-pharmacological (behavioral or cognitive-behavioral therapy). These treatment modalities have demonstrated incontrovertible short-term benefits across core symptoms of ADHD and associated impairments. However, there are limitations to current best practices for the treatment of ADHD.

With respect to pharmacological interventions, they are accompanied by adverse events in many children, do not normalize functioning, and adherence rates over time are generally low. The latter aspect is likely related to the fact that longer-term benefits of medication for ADHD are modest. Longitudinal studies have shown that acute improvements in ADHD symptoms and functioning are not maintained beyond 24-36 months.

With respect to non-pharmacological interventions, access to such services can be a challenge for a number of reasons, including the number of qualified providers and coverage by third-party payers.

Given these limitations to current standards of care, there is a strong need for the development of additional interventions for ADHD that can be easily and widely disseminated. Given the central role of attention and other aspects of cognition in the pathophysiology of ADHD, interventions that specifically target these constructs in safe and effective ways are particularly needed.

In recent years, there has been considerable interest in the use of computer-based software and to improve various aspects of cognition across a range of clinical populations, including those with ADHD. The CogMed Working Memory Training platform was one of the first interventions in this genre to be explored specifically with ADHD and showed promising results in several randomized studies.

Although there are multiple products in the marketplace that make various claims about effects on cognition in general, and in some cases, ADHD specifically, there have been no large scale, randomized trials of a digital therapeutic intervention versus an educational control intervention. Additionally, no approved treatments have been designed to provide an engaging, consumer-level experience that will promote high compliance when administered remotely (i.e., outside of a clinic).

This study will fill this gap with a large (minimum of 300 participants) randomized parallel group, controlled trial. Participants will be randomized (1:1) to receive either the *EVO* Multitask experimental intervention or the EVO *Words* educational control and instructed to play the digital therapies for typically 25 minutes/day for 5 days/week over a 4 week treatment period. Change in Baseline on the Attention Performance Index from the TOVA 8 will serve as the primary outcome variable and will allow for a determination of whether *EVO* Multitask significantly improves attentional functioning in pediatric participants with ADHD.

Secondary measures will include measures of ADHD symptoms, other measures of cognition and executive functioning, and measures of patient and parent/caregiver experience and satisfaction.

The therapeutic play regimen for the current study (typically 25 minutes/day; 5 days/week; 4 weeks) was established based on previous work with the *EVO* Multitask digital therapy. Specifically, a proof-of-concept study demonstrated that this regimen resulted in significant improvements in API scores in a sample of children with similar characteristics as proposed for this study. Results from the current study may also inform the development of additional dose-finding studies to determine whether additional (or less) game play might yield different results. It will also form the basis of additional studies to examine the durability of the intervention over time.

2. OBJECTIVES

To evaluate the effects of EVO Multitask game-based digital therapy versus EVO Words game-based digital therapy on attentional functioning (measured by the TOVA), ADHD symptoms, cognitive/executive functioning, and clinical impairment, in children diagnosed with ADHD.

2.1 Primary Objective

To evaluate the effects of EVO Multitask play versus EVO Words play on attentional functioning (measured by the API change score from the TOVA 8).

2.2 Secondary Objectives

To evaluate the effects of EVO Multitask play versus EVO Words play on ADHD symptoms (measured via a clinician and parent rating scales of ADHD symptoms)

To evaluate the effects of EVO Multitask play versus EVO Words play on objective working memory as measured by the CANTAB Spatial Working Memory (SWM) computerized test.

To evaluate the effects of EVO Multitask play versus EVO Words play on objective inhibition as measures by the TOVA commission error standard score in TOVA half-2.

2.3 Exploratory & Descriptive Objectives

To explore the percentage of responders (defined in the Statistical Analysis Plan) to primary and secondary objectives along with descriptive analysis of child/parent surveys, treatment regimen compliance, demographics, secondary symptom / executive function measures adjusted for baseline parent expectation, and any resulting treatment related AEs. In addition multiple sensitivity analyses will be conducted to assess the effects of site poolability, age, gender, missing data (if appropriate), and baseline TOVA API.

3. SUBJECT SELECTION

3.1 Inclusion Criteria

To be eligible for this trial, participants must meet all of the following criteria:

1. Age 8 years to 12 years, inclusive, at the time of parental informed consent
2. Male or female
3. Confirmed ADHD diagnosis, any presentation, at Screening based on DSM-V criteria and established via the MINI-KID administered by a trained clinician
4. Screening/Baseline (Visit 1 or 1a) score on the clinician-rated ADHD-RS-IV score $\geq$ 28
5. Screening/Baseline (Visit 1 or 1a) score on the TOVA API $\leq$ -1.8
6. Not undergoing pharmacological treatment with methylphenidate or amphetamine-based products at time of Screening; or, if undergoing pharmacological treatment, must be willing and appropriate (i.e. not optimally treated in the investigator's judgment) to wash out of current regimen.
7. Ability to follow written and verbal instructions (English), as assessed by the PI and/or study coordinator
8. Estimated IQ score $\geq$ 80 as assessed by the Kaufmann Brief Intelligence Test, Second Edition (KBIT-II)
9. Ability to comply with all the testing and requirements.

3.2 Exclusion Criteria

If a subject meets any of the following criteria, he or she may not be enrolled in the study:

1. Current, controlled (requiring a restricted medication) or uncontrolled, comorbid psychiatric diagnosis , based on MINI-KID and subsequent clinical interviewing, with significant symptoms including but not limited to post-traumatic stress disorder, psychosis, bipolar illness, pervasive developmental disorder, severe obsessive compulsive disorder, severe depressive or severe anxiety disorder, conduct disorder, or other symptomatic manifestations that in the opinion of the Investigator may confound study data/assessments. Participants with clinical history of learning disorders will be allowed to participate, provided the disorder does not impact their ability to participate in the trial based on PI judgment
2. Participants who are currently treated with a non-stimulant medication for ADHD (i.e., atomoxetine, clonidine, guanfacine)
3. Initiation within the last 4 weeks of behavioral therapy. Participants who have been in behavior therapy consistently for more than 4 weeks may participate provided their routine is unchanged during the

course of the study. Participants planning on changing or initiating behavior therapy during the course of the study will be excluded.

4. Participant is currently considered a suicide risk in the opinion of the Investigator, has previously made a suicide attempt, or has a prior history of, or is currently demonstrating active suicidal ideation or self-injurious behavior as measured by C-SSRS at screening.
5. Motor condition (e.g., physical deformity of the hands/arms; prostheses) that prevents playing the digital therapy as reported by the parent or observed by the investigator.
6. Recent history (within the past 6 months) of suspected substance abuse or dependence.
7. History of seizures (exclusive of febrile seizures), or significant motor or vocal tics, including but not limited to Tourette's Disorder.
8. Has participated in a clinical trial within 90 days prior to screening.
9. Diagnosis of or parent-reported color blindness (Confirmed in-clinic via ICBT)
10. Uncorrected visual acuity (Confirmed in-clinic via ability of subject to play the game)
11. Regular use of psychoactive drugs (non-stimulant) that in the opinion of the Investigator may confound study data/assessments.
12. Any other medical condition that in the opinion of the investigator may confound study data/assessments.
13. Has a sibling also enrolled/currently participating in the same study
14. Has previously participated in a study of Akili's videogame-like digital therapy

3.3 Concomitant Medications and Treatment

1. With the exception of common over the counter (OTC) (e.g. ibuprofen, acetaminophen) and prescription medications (e.g. antibiotics) for minor transient ailments, regular use of concomitant medications (i.e., sleep aids, etc.) are not permitted during the study. Section 6 lists allowed and prohibited medications. Transient use of some medications (i.e., non-sedating antihistamines to treat seasonal allergies) may be permitted on a case-by-case basis with PI and medical monitor review/approval. Families should advise the study staff if it becomes necessary to use other types of medication. Study investigators can approve short-term use of other medications that are not anticipated to confound study assessments.

2. Initiation of or significant changes in non-pharmacological treatment for ADHD is not permitted during the course of the study. Participants actively engaged in non-pharmacological treatment at Screening, may

be eligible if they meet all inclusion criteria and do not undergo changes to their treatment during the course of the study.

4. STUDY DESIGN

4.1. OVERVIEW OF STUDY

The study will be a blinded (investigators and outcome assessors) randomized, parallel group, controlled trial of EVO Multitasking game-based digital therapy versus EVO Words game-based digital therapy.

The trial will consist of 3 primary phases: Screening, Washout/Baseline, and Treatment. During the Screening Phase (Day -28 to Day -7), participants will undergo screening to evaluate eligibility for the study. Screening may take place up to 28 days before the Baseline Visit (Day 0). For those children currently on medication for ADHD the Washout period will begin 7 days prior to Baseline where treatment will be discontinued. On Day 0, the Baseline visit will occur wherein additional eligibility criteria will be established (ADHD-RS scores and API scores). The Treatment Phase (Day 1 to Day 27) will involve playing the digital therapy at home for each participant followed by an In-Clinic assessment on Day 28 to assess key outcomes. Compliance with play requirements will be monitored remotely during this phase.

Participants for this study will be male and female children between the ages of 8 years, 0 months and 12 years 11 months at the time of consent who meet full criteria for ADHD, any subtype, as assessed by the MINI-KID. Children must also be medically healthy, willing to discontinue any ongoing treatment, and free from other significant psychiatric comorbidities.

Two different electronically administered digital therapies will comprise the treatments under study:

Treatment #1 will consist of Project: EVO Mobile Game-Based Digital Therapy Intervention (EVO Multitasking therapy), a proprietary system based on a prototype cognitive training videogame called Neuroracer. EVO Multitasking digital therapy was designed to incorporate the fundamental features of Neuroracer into a state-of-the-art mobile video game-like platform, which deploys modern videogame graphics, engaging reward loops, and real-time adaptive mechanics to dynamically personalize difficulty based on the user's ability. EVO Multitasking therapy employs a perceptual discrimination attention/memory task as well as a continuous motor 'driving' task. Performance on these tasks are assessed in isolation and when performed together to calculate a performance index for each individual user. A personalized multi-task training regimen is automatically configured and delivered to the user, and is optimized adaptively to increase multi-task performance. As players proceed through the therapy periodic re-calibration occurs to maintain an optimal difficulty level.

Treatment #2 will consist of the EVO Words Mobile Game-Based Digital-Therapy Intervention (EVO Words Therapy), which is based off a popular Brain Training

app available in the iTunes app store. In the game, the player spells as many words as possible by connecting letters on a grid. He or she can connect the letters in any direction by touching the starting square and dragging his or her finger along the desired path. Each word created scores points for the player. Longer words and use of unusual letters increases the score of a word. Once the player can no longer find new words on the grid, the player can generate a new grid by pressing a "shuffle" button. The "shuffle" button deducts points from the players score. EVO Words contains 12 levels. As the levels increase, the size of the grid decreases. The goal of the game is to accumulate as many points as possible.

For each intervention condition, treatment will involve game-play on the system for approximately 25 minutes/day at least 5 days a week, for a total of 8 hours and 20 minutes of total game play. Compliance will be monitored electronically.

The primary endpoint for the study will be between-group comparison of the difference between the Baseline (Day 0) and Endpoint (Day 28) measure of the API score from the TOVA 8.

Figure 1 below provides an overview of the study design and assessments.

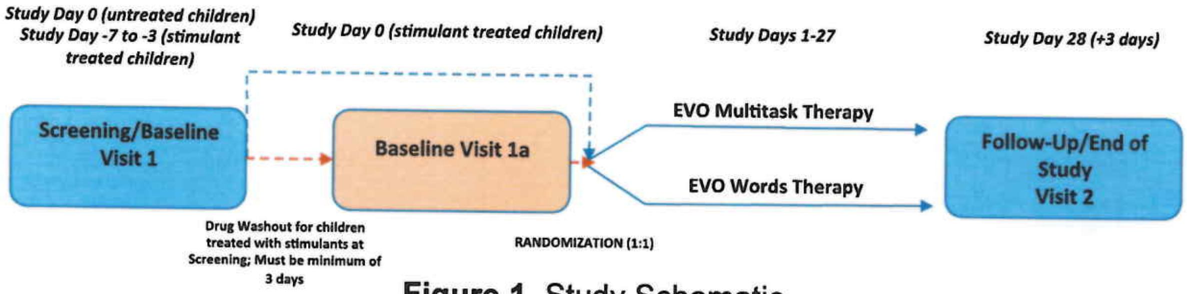

Figure 1. Study Schematic.

4.2 Treatment Arms

Each of the two treatment arms for the study, EVO Multitasking therapy and EVO Words therapy will be administered in similar format via a standardized tablet (i.e., iPad) provided to participants by the sponsor. The visual layout (i.e., color scheme) will be similar for each of the two interventions. The content of the two interventions will be different. EVO Multitasking will involve a character moving through a game-like space, while the EVO Words will involve letters and a grid as described above

PERIOD	Screening/Baseline[a]	*Washout*[a]	*Baseline*[a]	At-Home Intervention	Post-Treatment Visit
		Stimulant washout group only			
Visit	1		*1a*	-	2
Day	0 or up to 7 days prior to Baseline for those children requiring washout		*0*	1 - 27	28
Informed consent and child assent	X				
Demographic & contact information	X				
Developmental & Medical History	X				
MINI-KID	X				
Kaufmann Brief Intelligence Test, Second Edition	X				
C-SSRS assessment	X				X
Ishihara Color Blindness Test	X				
Discontinue Medication		*X*[c]			
TOVA assessment	X[d]		*X*[e]		X
CANTAB Spatial Working Memory	X[d]		*X*[e]		X
ADHD-RS	X[d]		*X*[e]		X
Parent BRIEF	X[d]		*X*[e]		X
Impairment Rating Scale	X[d]		*X*[e]		X
Clinical Global Impressions Scale[b]	X[d]		*X*[e]		X
Randomization	X[d]		*X*[e]		
EVO Multitasking intervention or *EVO Words* intervention in-clinic assessment (+Parent Expectation Survey)	X[d]		*X*[e]		
At-home *EVO Multitasking intervention* or *EVO Words* intervention play				X	

Optional weekly performance report to parents				X	
Compliance monitoring and feedback[f]				X	
Patient Experience/Satisfaction Measure- Parent Version					X
Patient Experience/Satisfaction Measure- Child Version					X
Safety assessments /AEs and concomitant medication / therapy	X	X	X		X
Collect iPads & Return to Akili for erasing					X[g]

[a]Participants who are not currently on ADHD medications at Screening will have a combined Screening/Baseline visit where all measures are completed. For participants currently treated with stimulant medications and who are eligible to initiate a washout period, a separate Baseline visit will be scheduled 3-7 days after Screening where the additional measures will be completed.

[b]For CGI measure, CGI Severity Scale will be assessed at Screening and CGI-Improvement Scale will be assessed at the Follow-Up Visit.

[c]To be initiated for participants who are currently treated with psychostimulants for ADHD at Screening; and for whom study participation is still indicated based on procedures described in Section 4.5. Washout should be scheduled at Screening visit and overseen by a qualified provider at each study site.

[d]For children currently not treated with stimulant medication, these procedures will occur at Visit 1.

[e]For children currently treated with stimulant medication who are eligible to continue following a washout of medication, these procedures will occur at Visit 1a.

[f]Compliance with intervention play requirements will be monitored via automatic uploading of play information to DCRI and a daily email report to DCRI and the clinical sites from the sponsor or their designated representative. The sites will provide feedback to the parents (via a pre-established contact method and will indicate when play drops below the minimum requirements.

[g]Sites are required to return the device to Akili for complete erasing of all data entered prior to re-circulation of the device. This process must be documented on the iPad Accountability Log.

4.3 Screening/Baseline Procedures

At the Screening/Baseline Visit (Visit 1) participants' parents or legal caregivers will be asked to provide informed consent; children will provide assent that will be documented appropriately. Participants and their parents/caregivers will then undergo a comprehensive evaluation to determine eligibility and whether inclusion/exclusion criteria are met. All of the following assessments will be recorded in the CRF if the subject is recruited into the study. Medical and psychiatric history, and demographic information will be obtained. Current medications and those taken during the past 30 days. Lifetime history of ADHD treatments taken will be obtained. The Kaufman Brief Intelligence Test, Second Edition will be administered to children and they must score ≥ 80 to continue in the study. The MINI-KID, a semi-structured interview, will be administered by qualified and trained clinicians to confirm ADHD diagnosis and determine whether psychiatric comorbidities are present. The C-SSRS, CGI-Severity, Ishihara Color Blindness Test, Impairment Rating Scale will then be completed. Ongoing medical issues will be assessed. Participants

Specific Procedures to be completed at the Screening/Baseline Visit (Visit 1) **for all participants** include:

- Informed consent with parent/caregiver; assent with child
- Demographic and Family information: Age and gender should be recorded in the screening log and the full demographics should be recorded in the CRF.
- Developmental and Medical History: Should be recorded in the screening log and in the CRF.
- MINI-KID completed by a trained clinician: Should be recorded in the screening log and in the CRF.
- C-SSRS (Lifetime): Should be recorded in the screening log and in the CRF.
- Ishihara Color Blindness Test: Should be recorded in the screening log and in the CRF.
- Kaufman Brief Intelligence Scale, Second Edition: Age-adjusted raw scores and percentiles for Matrices, Vocabulary, and Composite should be recorded in the screening log and in the CRF.

- Assessment of Ongoing Medical Issues/Concomitant Treatments: Should be recorded in the CRF.

Note that the following procedures will take place at the Screening/Baseline Visit (Visit 1) **ONLY FOR THOSE PARTICIPANTS WHO ARE NOT CURRENTLY TREATED WITH PSYCHOSTIMULANTS**:

The child will complete the following procedures:

- TOVA; Child must score ≤-1.8 on the API to remain eligible: The TOVA test will take approximately 25 minutes to complete. Separate instructions will be provided to the blinded raters to administer this test and to download and save the test results. The TOVA API score should be recorded in the CRF.

- CANTAB Spatial Working Memory task: The CANTAB SWM task will take approximately 9 minutes to complete. Separate instructions will be provided to the blinded raters to administer this test and to download and save the test results

While the child completes the TOVA and CANTAB, parents will complete the following procedures:

- Clinician completed ADHD-RS; must score ≥28 to remain eligible: The results of the ADHD-RS should be recorded in the CRF.

- Parent-Completed BRIEF: The parents should be instructed by a blinded rater to focus their evaluation over their child's past week behavior. The BRIEF Raw, T, %ile, negativity, and inconsistency scores should be recorded in the CRF.

- Impairment Rating Scale: A blinded clinical rater will review the Impairment Rating Scale with the parent. The results of the Impairment Rating Scale should be recorded in the CRF. Note that this scale was developed as a parent-reported instrument and severity ratings are often made via a visual analog scale. For the current protocol, the blinded clinical rater should solicit information about the areas of impairment for each subject and, using their clinical judgment, rate the severity of the impairment across functional domains on a 7 point scale.

- CGI-Severity Scale. A blinded rater will complete the CGI-Severity scale based on all available information. The results of the CGI-Severity scale should be recorded in the CRF.

- Randomization: The participants will be provided with an iPad which is engraved in the back with a serial number ("AL-###"). The iPads will be preloaded with either EVO Multitasking therapy or EVO Words therapy. A separate document will list the blinded treatment assignment.

- Break: Participants should be allowed a 10 minute break following the TOVA / CANTAB tests. During the break they may have a snack but no caffeinated beverages are allowed

- In-Clinic Assessment/Training for EVO Multitasking therapy OR EVO Words therapy (depending upon randomization). The subject will only practice on the therapy to which they were randomized. The subject should play either EVO Multitask or Words for approximately 10 minutes. (Note: The parents should observe their child interact with the therapy as they will be asked to rate their expectations of possible benefit). Separate instructions will be provided to an unblinded clinical coordinator to log the subject into either the EVO or Words therapy. During the 10 minutes of in-clinic play, the unblinded coordinator should observe the participants play to ensure he or she can follow the rules (which are in English) of their assigned therapy. The unblinded coordinator should also make note of any motor conditions or uncorrected visual acuity that prevents proper game play. Participants that are unable to properly play their therapy will be withdrawn from the study. Note: For EVO Words the participant should be able to spell at least two words during the assessment. At the end of approximately 10 minutes the unblinded coordinator should have the subject stop his/her play (at an obvious break point in the game) and turn the iPad off. At this point, the parent should complete the Baseline Expectation Survey

- Materials to go home: An unblinded coordinator should provide the parents with of a copy of their ICF and child assent along with the instructions for their child's therapy. The parents should be told that their child is expected to play their therapy for typically 25 minutes/day, 5 days/week. **NOTE: The parent and child should be told that no one except the child may play the therapy for any reason.** The child should be provided with same iPad they previously played in the clinic along with a battery charger. In addition, both parent and participant should be instruction on intended and unintended use of the device. These are defined as:

    - Intended use- Set up of the participant's local WiFi network for internet access to push play and compliance data to the Akili secure severs, and completion of at-home interventional play for the assigned 25 minutes per day, during 5 days of each calendar week.

- Unintended use – Any and all other features or applications of the device, including accessing the internet, downloading of additional applications, use of features such as music, calendar, or notes, and inputting any personal information other than participant's home WiFi network as described above.

4.4 Washout Procedures & Screening/Baseline (Post Washout) Procedures

For participants who are currently treated with psychostimulants for ADHD and are still eligible to continue in the trial following Screening, procedures for washout and Baseline will be discussed with the parents/caregivers.

Qualifications for treated participants to enroll in trial: Participants who meet full criteria for ADHD at Screening based on the MINI-KID, and who the PI believes are not optimally treated with current medication will be eligible to washout of their current medication regimen and enroll in the trial. Suboptimal response to current treatment may be assessed by the PI on the basis of MINI-KID symptom presentation and CGI-Severity scores that are 3 (minimally impaired) or higher.

For those participants eligible to enroll and initiate washout of their current medication, treatment will be discontinued between 7 and 3 days prior to their scheduled Screening/Baseline Visit (Visit 1a). Study staff will be available via phone or unscheduled visit during this period should clinical issues rise that warrant attention.

Note that the following procedures will take place at the Screening/Baseline (Visit 1a) **ONLY FOR THOSE PARTICIPANTS WHO WASHED OUT/ DISCONTINUED PSYCHOSTIMULANTS.** Following their washout period, during Visit 1a, the child will complete the following procedures:

- TOVA; Child must score ≤-1.8 on the API to remain eligible. The TOVA test will take approximately 25 minutes to complete. Separate instructions will be provided to the blinded raters to administer this test and to download and save the test results. The TOVA API score should be recorded in the CRF.

- CANTAB Spatial Working Memory task: The CANTAB SWM task will take approximately 9 minutes to complete. Separate instructions will be provided to the blinded raters to administer this test and to download and save the test results

While the child completes the TOVA and CANTAB, parents will complete the following procedures:

- Clinician completed ADHD-RS; must score ≥28 to remain eligible: The results of the ADHD-RS should be recorded in the CRF.

- Parent-Completed BRIEF: The parents should be instructed by a blinded rater to focus their evaluation over their child's past week behavior The BRIEF Raw, T, %ile, negativity, and inconsistency scores should be recorded in the CRF.

- Impairment Rating Scale: A blinded clinical rater will review the Impairment Rating Scale with the parent. The results of the Impairment rating Scale should be recorded in the CRF. Note that this scale was developed as a parent-reported instrument and severity ratings are often made via a visual analog scale. For the current protocol, the blinded clinical rater should solicit information about the areas of impairment for each subject and, using their clinical judgment, rate the severity of the impairment across functional domains on a 7 point scale.

- CGI-Severity Scale. A blinded rater will complete the CGI-Severity scale based on all available information. The results of the CGI-Severity scale should be recorded in the CRF

- Randomization: The participants will be provided with an iPad which is engraved in the back with a serial number ("AL-###"). The iPads will be preloaded with either EVO Multitasking therapy or EVO Words therapy. A separate document will list the blinded treatment assignment.

- Break: Participants should be allowed a 10 minute break following the TOVA / CANTAB tests. During the break they may have a snack but no caffeinated beverages are allowed

- In-Clinic Assessment/Training for EVO Multitasking therapy OR EVO Words therapy (depending upon randomization): The subject should play: either EVO Multitask or EVO Words for approximately 10 minutes. (Note: The parents should observe their child interact with the therapy as they will be asked to rate their expectations of possible benefit). Separate instructions will be provided to an unblinded clinical coordinator to log the subject into either the EVO Multitask or EVO Words therapy. During the 10 minutes of in-clinic play, the un-blinded coordinator should observe the participants play to ensure he or she can follow the rules (which are in English) of their assigned game. The un-blinded coordinator should also make note of any motor conditions or uncorrected visual acuity that prevents proper game play. Participants that are unable to properly play their therapy will be withdrawn from the study. Note: For EVO Words the participant should be able to spell at least two words during the assessment. At the end of approximately 10 minutes the unblinded coordinator should have the subject stop his/her play (at an obvious break point in the game) and turn the iPad off. At this point, the parent should complete the Baseline Expectation Survey

- Materials to go home: An un-blinded coordinator should provide the parents with of a copy of their ICF and child assent along with the instructions for their child's therapy. The parents should be told that their child is expected to play their therapy for typically 25 minutes/day, 5 days/week. ***NOTE: The parent and child should be told that no one except the child may play the game for any reason.*** The child should be provided with same iPad they previously played in the clinic along with a battery charger. In addition, both parent and participant should be instructed on intended and unintended use of the device. These are defined as:
    - Intended use- Set up of the participant's local WiFi network for internet access to push play and compliance data to the Akili secure severs, and completion of at-home interventional play for the assigned 25 minutes per day, during 5 days of each calendar week.
    - Unintended use – Any and all other features or applications of the device, including accessing the internet, downloading of additional applications, use of features such as music, calendar, or notes, and inputting any personal information other than participant's home Wi-Fi network as described above.

4.5 Treatment Procedures – In-Home Game Play and Post-Treatment Visit

Beginning on the first day after the Screening/Baseline Visit (Visit 1 for untreated children at Screening; Visit 1a for those children requiring medication washout), participants will initiate at-home play with their randomized therapy (EVO Multitasking or EVO Words). The therapy will pick up from where it left off during the in-clinic session. Participants and their parents will be instructed during Screening and Baseline that the expectations for play will be typically 25 minutes/day, 5 days/week.

For each play episode, participants will logon to their assigned iPad with a unique ID and initiate therapeutic play. Log on passwords and game instructions will be provided separately. Details about performance and duration of play will be recorded and uploaded to central servers (when the iPad is connected to Wi-Fi) for data capture. Compliance with therapy play requirements will be monitored automatically and sites will receive daily email notifications. For participants not meeting the requirements, email reminders and instructions will be provided. Additionally, EVO Multitasking and EVO Words both contain built-in features that remind the participant to play. Participants who fail to meet play requirements will be contacted by an un-blinded site clinical coordinator to troubleshoot

problems and problem-solve to increase compliance (Scripts for these communications will be described in a separate document). During the demographic data collection, the parent should specify the preferred method by which they would like to be contacted (e.g. phone, text, or email). Additionally parents will receive a weekly and automatically generated progress email report directly from the Sponsor (Akili) by sharing their email at informed consent. However, parents may elect to opt-out at the time of informed consent.

On day 21 the study site should remind the parents by phone, preferably (email and/or text are also acceptable), that their child is entering the last week of the study (A script for this communication will be described in a separate document).

The post-treatment visit (Visit 2 will be scheduled to occur 4 weeks after the Baseline Visit. If the parent and child are unable to return to the clinic on Day 28, Day 28 + 3 days is acceptable (the subject should be encouraged to continue to play their therapy during this exception period). Children who have been ill or taken medication within 48 hours of their scheduled Visit 2 should contact DCRI CRA and the medical monitor to advise regarding scheduling, In general, participants should not be scheduled for their Visit 2 if they have been sick enough to skip school, or if they have taken any medications that may have impacted functioning within 48 hours. At this visit, participants will be re-assessed for attentional functioning, ADHD symptoms, and impairment. Additional information regarding satisfaction and experience with the therapy will be collected from both participants and their parents/caregivers.

NOTE: The parent and subject should return to the clinic at approximately the same time of day (i.e. morning or afternoon) as was done for the Screening/Baseline Visit (Visit 1 or 1a for untreated or treated children, respectively). Assessments should also be administered in the same order per the Table of Procedures in Section 4.3. Also, all attempts should be made to ensure that the <u>same</u> parent/caregiver accompany the subject and complete the outcome measures. Also, all attempts should be made to ensure that the <u>same</u> study staff administers the subjective clinical outcome measures (ADHD-RS, CGI, and IRS).

Specific procedures to be completed during the Post-Treatment Visit include:

- C-SSRS (Since last visit): The results of the C-SSRS should be recorded in the CRF

- Clinician completed ADHD-RS: Results of the ADHD-RS should be recorded in the CRF

- Clinician Completed CGI Improvement Scale. The results of the CGI Improvement Scale should be recorded in the CRF

- Impairment Rating Scale. The results of the Impairment Rating Scale should be recorded in the CRF

- TOVA: The TOVA test will take approximately 25 minutes to complete. Separate instructions will be provided to the blinded raters to administer this test and to download and save the test results The TOVA API score should be recorded in the CRF.

- CANTAB Spatial Working Memory task: The CANTAB SWM task will take approximately 9 minutes to complete. Separate instructions will be provided to the blinded raters to administer this test and to download and save the test results

- Parent-Completed BRIEF: While the subject is performing the CANTAB SWM and TOVA, the parents will be asked to complete the BRIEF. The parents should be instructed by a blinded rater to focus their evaluation over their child's past week behavior. The BRIEF Raw, T, %ile, negativity, and inconsistency scores should be recorded in the CRF

- Patient Experience/Satisfaction Measure – Parent Version: The un-blinded rater will complete a parent survey. The results will be captured in the CRF

- Patient Experience/Satisfaction Measure – Child Version: The un-blinded rater will compete a child survey. The results will be captured in the CRF.

- Assessment of Adverse Events/Concomitant Treatment: AEs and Con-Meds will be captured in the CRF

- The iPad and battery charger will be returned to site

- Prior to re-circulation of the device to future participants, the device will be returned to Akili for complete erasing of all data entered by the participant.

4.6 Procedures to minimize bias and blinding to treatment assignment

Participants and their parents will not be aware of which of the interventions to be used in the study is the test device and which is the comparator. The clinicians and staff completing assessments will be blinded to which device each participant is assigned. An un-blinded staff member at each site will be

designated in order to provide initial instructions for use at the Screening/Baseline Visit, monitor compliance, and follow up with parents as required. Parents and participants will be instructed not to share instructions about the details of the intervention to which they were assigned to the study staff during their subsequent End of Treatment visit (Visit 2).). Additional details around the procedures to be used to minimize bias and blind participants/parents and site staff are as follows.

Since the EVO Multitasking therapy and the EVO Words therapy are fundamentally different, true blinding is not possible for this study. The following study procedures will be implemented to maximize the chances that 1) participants and their parents are not aware of which treatment condition is the test intervention and which is the comparator; and 2) that site staff involved in completing outcome measures are blinded to treatment assignment.

4.6.1 Procedures to minimize bias in parents and participants.

All communication between site staff and participants/parents should emphasize that this is a study to evaluate the effects of 2 different therapies on ADHD symptoms. This communication should be reviewed in the consent procedures. If parents raise questions about whether 1 of the treatments is expected to have a different effect, they should be informed that the study is designed to evaluate how they compare to one another on ADHD functioning. During Screening/Baseline, participants/parents will be reminded not to discuss the details or content of their assigned treatment with anyone at the site other than the un-blinded coordinator who will review the instructions for the randomized therapy with each participant. Participants/parent will also be discouraged from discussing the details/content of their assigned therapy with other children/families outside of the study.

4.6.2 Procedures to blind/minimize bias in site staff and investigators.

Following Screening and prior to randomization, investigators and site staff will remind participants/parents not to discuss the content/details of their assigned treatment with anyone at the site except the un-blinded coordinator. Upon returning to the clinical site for their Baseline 1a visit (for participants who require washout) and for their follow-up (Visit 2) visit, participants and their families will be reminded not to discuss the content/details of their assigned treatment with other site staff. Following randomization at the end of Visit 1 (or Visit 1a for participants requiring washout), participants/parents will only have direct contact with the unblinded coordinator who will review instructions for their assigned treatment condition. Participants/parents will be reminded not to discuss the content/details of their assigned treatment in the event of any phone calls or unscheduled visits that might occur prior to the Visit 2.

Investigators and other blinded site staff will not have access to any source documents or CRFs that may compromise the blind.

4.6.3 Procedures in the event of possible or probable unblinding.

If at any point after randomization an Investigator or other site staff responsible for completing any of the outcome measures becomes possibly or probably un-blinded, the following procedures should be implemented:

- A description of the un-blinding event should be generated and sent to the DCRI Coordinating PI (Kollins), Medical Monitor (Krystal) and Project Leader.
- If there are still study assessments that need to be completed, an alternative and blinded rater should be used, if available at the site (e.g., a Sub-I)
- If a blinded Sub-I or other trained and qualified blinded rater is not available, the measures should be completed by the unblinded rater.

Note: In the event of unblinding as described above or if study staff explicitly unblinds the parent or participant to the study hypothesis, the unblinding event will be noted in the CRF.

4.8 Randomization Procedures

Participants who meet all inclusion/exclusion criteria and sign the informed consent/assent will be enrolled. Randomization will occur during the baseline visit only after ADHD diagnosis is confirmed via the MINI-Kid. The treatment given to individual participants will be determined by a randomization schedule will be generated by the scheme prepared by Duke Clinical Research Institute (DCRI) statistics group using a validated computer software (SAS) that incorporates a standard procedure for generating random numbers. A copy of the randomization schedule scheme will be held by the DCRI Statistics Department. The randomization scheme will be monitored monthly through the enrollment period for accuracy. This schedule will be loaded into the clinical database randomization module. Participants randomized into the study will be assigned the treatment corresponding to the next available number in the randomization schedule. Any participants that show non-compliance as described in Section 8.5.1 will be replaced.

Participants will be randomized in a 1:1 ratio to receive either an iPad preloaded with EVO Multitasking therapy or an iPad preloaded with EVO Words therapy. Randomization will be stratified by drug washout or no drug washout. Group #1 - washed out of stimulant (at time of informed consent) and group #2 - wasn't on stimulant (at time of informed consent). Although no official gender stratification is indicated, study sites will use their best efforts to gender-match and enroll a minimum of 30% girls.

5 STUDY DEVICE

The study devices are NOT intended to be used for any purpose other than that outlined in this protocol.

Description of Study Device (EVO Multitasking therapy): EVO Multitasking therapy will be loaded by the sponsor (Akili) onto iPad Mini II tablets. The iPads are configured to minimize the participants' ability to utilize non-study related applications. Features such as browsing the internet, downloading apps, deleting apps, and messaging are restricted. However, some functions such as Music (which may provide for downloading via iTunes), Notes, Calendar, and Reminders may potentially be accessed by participants. Unblinded site staff will instruct participants and parents on intended and unintended use, as outlined in this protocol. The iPads along with chargers will be provided by the sponsor to the clinical sites. In a separate document, a list of logon credential (Subject IDs and Passwords) will be provided. All data entered by the participant onto the device will be erased before reusing the device.

Description of Study Device (EVO Words therapy): EVO Words therapy will be loaded by the sponsor (Akili) onto iPad Mini II tablets. The iPads are configured to minimize the participants' ability to utilize non-study related applications. Features such as browsing internet, downloading apps, deleting apps, and messaging are restricted. However, some functions such as Music (which may provide for downloading via iTunes), Notes, Calendar, and Reminders may potentially be accessed by participants. Unblinded site staff will instruct participants and parents on intended and unintended use, as outlined in this protocol. The iPads along with chargers will be provided by the sponsor to the clinical sites. In a separate document, a list of logon credential (Subject IDs and Passwords) will be provided. All data entered by the participant onto the device will be erased before reusing the device.

5.1 Study Device Usage

Participants and their parents/caregivers will be instructed to play their assigned therapy for a duration of typically 25 minutes/day for 5 days/week during each of the 4 weeks of the treatment phase of the study. For EVO Multitask 25 minutes/day is accomplished by playing 5 "sessions" of 4 to 5 minutes each. For EVO Words 25 minutes/day is accomplished via a displayed minute timer. Participants should initiate play on the first day after the Baseline Visit. Specifics about which days and times during those days can be used for play will be free to vary among the participants based on their schedules. Time of play will be captured. Therapeutic play should continue up until the day prior to the End of Treatment visit (Visit 2.).

Prior to initiating play, the subject should block out 25 minutes of uninterrupted time in a quite location where he or she can focus on playing their therapy. It's

very important that only the study subject plays the therapy. At no other time should anyone else be allowed to play the therapy.

Also, it's important to make sure the iPad is connected to the participants home Wi-Fi. If home Wi-Fi is not available the study staff and parents need to establish a plan where the iPad will be connected to remote Wi-Fi on a periodic basis during subject play (Note: The remote Wi-Fi location should not require webpage authentication). The iPad will cache all data locally but without Wi-Fi access the study sites will not receive accurate compliance reports

To initiate play, the subject taps on the game therapy icon displayed on the screen and follows the in-program instructions. Also, separate hard-copy instructions will be sent home with the parent. The instructions are specific to EVO Multitasking or EVO Words therapies, so the un-blinded clinical coordinator will need to provide these.

For any calendar day the therapy will automatically lock after approximately 25 minutes of play and no further play will be allowed until the next calendar day. For EVO Multitasking this lockout is accomplished after the subject completes 5 "sessions" of 4 to 5 minutes each. For EVO Words, the therapy stops after 25 minutes.

5.2 Potential Benefits/Risks of Study Device

Based on preliminary data obtained in several studies, including a Proof-of-Concept trial in children with ADHD, the potential benefits of the EVO Multitasking game-based therapy for this population are to increase attentional functioning. Given the central role of attention in the pathophysiology of ADHD, this benefit is substantial.

To date, there have been no adverse events reported across several early studies with the device. The risk/benefit ratio for use is favorable.

Use of the EVO Words therapy has not been evaluated systematically. However, no adverse events are expected with the amount of play required in the present study. Moreover, given the nature of the EVO Words therapy, expected benefit for the primary outcome measure and secondary measures is expected to be minimal.

The risks to a participant participating in this study are very small. The study involves completing some computerized tests, game-based digital therapies, and answering some questions. It is possible that the participant could become frustrated by some of the tasks. The participant could become fatigued by the computer tests or therapeutic play. If the participant becomes frustrated or fatigued, they may stop at any time. There is a risk that the participant may access unintended features (e.g. Music, Contacts, Reminders, Notes, or Calendar) during the course of their participation in the at-home portion of this study. In this instance, the participant may enter personal information onto the device. To circumvent associated risk, all data entered by the participant onto the

device will be erased before reusing the device. There are no other risks to taking part in this study of which we are aware.

The participant will not receive any direct benefit from participating in this study. No guarantee can be made concerning the study outcome, because results from a clinical research study cannot be predicted.

5.3 Procedures for Monitoring Compliance and Replacing Participants

Both EVO Multitask and EVO Words automatically capture gameplay compliance and upload these data directly to a central server when connected to Wi-Fi. The server will automatically push daily compliance emails to the clinical sites (the format of which is described in a separate document). Based on these emails, un-blinded study staff can then reach out to the parents or participants (via the preferred contact method captured in the demographic collection) to trouble shoot technical problems and/or encourage more play. The rules for compliance outreach follow:

1) Participants that fail to play any EVO Multitask or EVO Words sessions over two or more days will be contacted to trouble shoot potential Wi-Fi connection problems (These participants will be highlighted in yellow in the daily compliance email

2) Participants fail to play at least 5 sessions of EVO Multitask or 25 minutes of EVO Words over 3 or more days will be contacted and reminded to play more (These participants will be highlighted in yellow in the daily compliance email)

3) Participants that fail to play any EVO Multitask or EVO Words sessions over 7 days will be replaced (These participants will be highlighted in red in the daily compliance report). These participants should NOT be told they are being replaced and should be encouraged to keep playing (as with other participants) and reminded to return to the clinic for their exit visit at the end of the study.

4) Participants that fail to play at least 12 sessions of EVO Multitask or 60 minutes of EVO Words over 14 days will be replaced (These participants will be highlighted in red in the daily compliance report). These participants should NOT be told they are being replaced and should be encouraged to keep playing (as with other participants) and reminded to return to the clinic for their exit visit at the end of the study.

Additionally, parents will receive a weekly and automatically generated progress email report directly from the Sponsor (Akili), unless they have opted-out at the time of informed consent.

Note: In the event a subject does not return to the clinic for their final exit visit or does not complete their assessments during the exit visit, this subject will be replaced.

5.4 Discontinuation of Study Device

Participants may withdraw at any time during the study without prejudice or be discontinued from study treatment at the discretion of the investigator if medically necessary or if any untoward effects occur. In addition, a subject may be withdrawn by the investigator or DCRI if the subject is noncompliant with their assigned treatment (as described above) or otherwise violates the study plan, or for administrative and/or other safety reason. In the case where a subject withdraws from the study, the subject will be replaced.

The investigator or designee will notify the sponsor or their designee immediately when a subject has been discontinued or withdrawn from study treatment because of an adverse experience. When a subject discontinues or is withdrawn from treatment before study completion, all applicable activities scheduled for the final study visit should be performed at the time of discontinuation. Any adverse experiences that are present at the time of discontinuation/withdrawal should be reported and followed up in accordance with the safety requirements outlined in Section 7.0 (Adverse Events).

6 CONCOMITANT MEDICATIONS

Central nervous system stimulants will be discontinued at the beginning of the washout phase and not allowed for the duration of the trial.

6.1 Allowed Medications

Non-sedating antihistamines
Analgesics including acetaminophen and ibuprofen
Antibiotics required for the treatment of minor illnesses
Vitamins

6.2 Prohibited Medications

Prohibited medications 30 days prior to screening (for this purpose, defined as the day of informed consent) and through the duration of the trial include:
SSRIs (e.g., fluoxetine, paroxetine)
MAOIs (monoamine oxidase inhibitors)
Mood stabilizers (e.g., lithium, valproate, quetiapine)
Antipsychotics (e.g., risperidone, olanzapine)
Anticonvulsants (e.g., phenobarbital, phenytoin, primidone)
Anticoagulants
Dextromethorphan
Halogenated anesthetics
Tricyclic antidepressants
Atomoxetine
Guanfacine
Clonidine
Phenylbutazone
Sedating antihistamines
Decongestants with stimulant properties

7.0 ADVERSE DEVICE EFFECT REPORTING AND FOLLOW-UP

7.1 Definitions

Adverse Device Effect (ADE)

Adverse event related to the use of an investigational medical device. This includes any adverse event resulting from insufficiencies or inadequacies in the instructions for use, the deployment, the implantation, the installation, the operation, or any malfunction of the investigational medical device. This also includes any event that is a result of a use error or intentional misuse.

Unanticipated Adverse Device Effects (UADEs)

Per 21 CFR 812.3, an Unanticipated Adverse Device Effect is any serious adverse effect on health or safety or any life-threatening problem or death caused by, or associated with, a device, if that effect, problem, or death was not previously identified in nature, severity, or degree of incidence in the investigational plan or application (including a supplementary plan or application), or any other unanticipated serious problem associated with a device that relates to the rights, safety, or welfare of participants.

Unanticipated Adverse Device Effects will include events meeting either A or B as stated below:

A. Events meeting ALL of the following criteria:
   - *Not included in the list of Anticipated Events* (refer to I protocol section 7.3)
   - *Possibly, probably, or definitely related to the* investigational device per the site principal investigator

     Serious (meets any of the following criteria):
     - Is life-threatening illness or injury
     - Results in permanent impairment of a body structure or a body structure
     - Necessitates medical or surgical intervention to prevent permanent impairment of a body function or a body structure
     - Led to fetal distress, fetal death or a congenital abnormality or birth defect
     - Led to death

   (*Permanent* means irreversible impairment or damage to a body structure or function, excluding trivial impairment or damage).

B. Any other unanticipated serious problem associated with the investigational device that relates to the rights, safety, or welfare of participants.

7.2 Assessment

7.2.1Time Frame

An ADE observed from the time of randomization through the final study visit (day 28) will be collected in clinical database eCOS

7.2.2 Causality Rating

The causal relationship of an adverse event to either study game will be rated as follows:

Possible: An event that might be due to the use of the study device. An alternative explanation - e.g., concomitant drug(s), concomitant disease(s) - is inconclusive. The relationship in time is reasonable; therefore the causal relationship cannot be excluded.

Probable: An event that might be due to the use of the study device. An alternative explanation is less likely - e.g., concomitant drug(s), concomitant disease(s). The relationship in time is suggestive.

Definitely: An event that is due to the use of the study device. The event cannot be reasonably explained by an alternative explanation - e.g., concomitant drug(s), concomitant disease(s).

7.2.3 Severity of Adverse Device Effects

The severity of an ADE will be rated as follows:

- **Mild**: Awareness of sign, symptom, or event, but easily tolerated.
- **Moderate**: Discomfort enough to cause interference with usual activity and may warrant intervention.
- **Severe**: Incapacitating with inability to do usual activities or significantly affects clinical status and warrants intervention.

7.3 Previously Noted Adverse Device Effects

The expectedness of an ADE shall be documented in the informed consent (ICF). Any ADE that is not identified in nature, severity, or is not listed below is considered unanticipated.

Adverse Device Effects EXPECTED FOR THIS DEVICE:

1. Dizziness
2. Nausea
3. Headache
4. Increased and unusual levels of frustration during game play

7.4 Reporting

It is understood that complete information about an event may not be known at the time the initial report is submitted. The investigator must assess the relationship of the event to the investigational device (including rationale for assessment) and should make every attempt to obtain as much information as possible concerning the event. Additional information pertaining to an event should be reported in the EDC as it becomes available.

7.4.1 Adverse device effect

The investigator must report, via eCOS, all Adverse device effects, anticipated or unanticipated, occurring from the time of randomization through the final study visit (day 28) within 24 hours of knowledge of the event. If the eCRF system is temporarily unavailable, the event, including investigator-determined causality assessment, should be reported to DCRI via a paper back-up ADE form. Upon return of the availability of the eCOS system, the ADE information must be entered into the eCRF.

The investigator must report, via eCOS, when important follow-up information (final diagnosis, outcome, results of specific investigations, etc.) becomes available after submission of the initial Suspected Adverse Reaction information. Follow-up information should be submitted according to the same process used for reporting the initial event as described above (i.e., within 24 hours of knowledge, via eCOS). All adverse device effects will be followed through resolution.

DCRI Safety Surveillance will report all adverse device effects to specified trial personnel (sponsor, principal investigator [PI] and project leader)

within 1 to 2 business days of receipt.

7.4.2 Regulatory Reporting of Unanticipated Adverse Device Effects

If an investigator reports an UADE to the sponsor (via the EDC), the sponsor shall immediately conduct an evaluation of the UADE and will make a determination as to whether it meets the "unanticipated" criteria (i.e., are not listed in section 7.3 of the protocol) and whether it meets the criteria for reporting to regulatory authorities (e.g. FDA, IRBs).
Any UADE(s) that the sponsor determines is/are reportable will be submitted to the FDA, all reviewing IRBs, and all participating investigators within 10 working days of when the sponsor makes that determination.

If the sponsor determines that an UADE presents an unreasonable risk to participants, all investigations or parts of investigations presenting that risk shall be terminated as soon as possible. Termination shall occur not later than 5 working days after the sponsor makes this determination and not later than 15 working days after the sponsor first received notice of the effect.

All Regulatory Reporting to the FDA will be done by DCRI Regulatory.

7.5. Institutional Review Boards

All sites will submit the study Adverse Device Effects, Unexpected Adverse Device Effects, Adverse Events and Serious Adverse Events to their local/central Institutional Review Board (IRB) according to their local IRB polices and procedure.

8.0 STATISTICAL ANALYSES

This section will provide an overview of the statistical, descriptive, and qualitative analyses for this study. Specific details of the analyses will be described in a separate statistical analysis plan (SAP)

The main objective of this study is as follows:

- To evaluate the effects of EVO Multitask game-based digital therapy versus EVO Words game-based digital therapy on attentional functioning (measured by the TOVA API score change) and ADHD symptoms (measured by gold-standard qualitative rating scales).

This study has 1 primary and 3 secondary objectives. Success on the Primary (P1) and the first Secondary Objective (S1) (Change in ADHD symptoms) will provide confirmatory support for the full claim above. If the primary is successful, but the ADHD symptom secondary objective fails to meet statistical significance, the modified claim below will still be supported:

- To evaluate the effects of EVO Multitask game-based digital therapy versus EVO Words game-based digital therapy on attentional functioning (measured by the TOVA API score change).

Below is a list of primary, secondary, exploratory, safety, and descriptive objectives. Missing data and site poolability are addressed as descriptive objectives. Details on measures collected are provided in the SAP.

8.1 Primary Objective (P1) - Attention

P1-Objective) To evaluate the effects of EVO Multitask play versus EVO Words play on attentional functioning (measured by the API composite score from the TOVA 8).

P1-Outcome Measure(s)) Change from baseline to day 28 on TOVA API

The TOVA is a clinically-relevant and validated continuous performance test (CPT) that informs attentional performance in ADHD populations. The API score is a composite variable that summarizes the overall result of the TOVA and is composed of age/gender adjusted (z-scores) of reaction time variability, mean reaction time, and sensitivity to the target stimuli (d')

8.2 Secondary Objectives

8.2.1 **Major Secondary Objective I (S1) – ADHD Symptoms**

S1-Objective) To evaluate the effects of EVO Multitask play versus EVO Words play on ADHD symptoms using clinically relevant qualitative rating scales of ADHD symptoms, executive function, and global function.

S1-Outcome Measures(s))

- ADHD-RS Total (Change from baseline to day 28)
- ADHD-RS Inattentive Sub-scale (Change from baseline to day 28)
- ADHD-RS Hyperactivity Sub-scale (Change from baseline to day 28)
- BRIEF Working Memory Percentile (Change from baseline to day 28)
- BRIEF Inhibit Percentile (Change from baseline to day 28)
- Impairment Rating Scale (IRS) (Change from baseline to day 28)
- Clinician Global Improvement (CGI) (at day 28)

8.2.2 Minor Secondary Objective II (S2) – Objective Working Memory

S2-Objective) To evaluate the effects of EVO Multitask versus EVO Words play on objective working memory function as measured by CANTAB's Spatial Working Memory (SWM) computerized test.

S2-Outcome Measure(s)) 8 CANTAB SWM outcome measures (Change from baseline to day 28)

- Mean time to first response (4 boxes)
- Mean time to last response (4 boxes)
- Mean token-search preparation time (4 boxes)

- S.D. time to first response (4 boxes)
- S.D. time to last response (4 boxes)
- S.D. token-search preparation time (4 boxes)
- Strategy (4 to 8 boxes)
- Total Errors

8.2.3 Minor Secondary Objective III (S3) – Objective Inhibition

S3-Objective) To evaluate the effects of EVO Multitask versus EVO Words play on inhibitory function as measured by TOVA Commission Error Standard Score (non-composite score) during the frequent response portion of the TOVA (COMSSH2).

S3-Outcome Measure(s)) - TOVA COMMSSH2

8.3 Exploratory Objectives

This section will list the planned exploratory analyses. Full details are provided in the SAP.

8.3.1 Exploratory Objective I (E1) - Responders

E1-Objective) To evaluate the percentage of responders for select outcome measures in both the EVO: Multi and EVO: Words groups.

E1-Outcome Measure(s)) The following outcomes will be examined;

- TOVA API
- ADHD-RS Total
- CGI
- IRS

Responder cut-off values and details of this analysis will be described in the SAP.

8.3.2 Exploratory Objective II (E2) – Additional TOVA Endpoints

E2-Objective) To evaluate the effects of EVO Multitask play versus EVO Words play on additional TOVA endpoints not analyzed in other objectives.

E2-Measures) Multiple TOVA outcomes (Change from baseline to day 28). See SAP for a list of measures.

8.4 Safety Analysis

Treatment related adverse events for both the EVO Multitask and EVO Words interventions will be presented. Any adverse events occurring during any phase of the study judged by clinical sites' Primary Investigator (PI) to be related to either intervention will be recorded and presented in a table listing. The Safety population (see section 8.6.2 for analysis population details) will be used for this analysis that will include randomized participants that began the at-home training phase for either intervention. The severity of each event will be evaluated by the PI and presented with the recorded events. If there are frequently occurring treatment related adverse events, a frequency table will be included, to rank events from most frequent to most rare. Subjects will be included in the actual treatment group received.

8.5 Descriptive Analyses

The following additional descriptive analyses will be conducted. Details are provided in the SAP.

- D1: Gameplay Surveys
- D2: Compliance
- D3: Age and Gender Sensitivity Analysis
- D4: Site Poolability Sensitivity Analysis
- D5: Missing Data Sensitivity Analysis
- D6: Baseline TOVA API sensitivity analysis
- D7: Baseline Parent Expectancy Sensitivity Analysis

8.6 General Statistical Methods

This section will present the formal hypotheses, analysis populations, multiplicity adjustment consideration, and methods for handling missing data. In addition, details about the planned interim analysis and statistical power are presented.

8.6.1 Hypothesis Tests and Multiplicity Adjustments

The aim of all primary and secondary objectives in this study are to demonstrate a statistical difference on outcome measure mean change scores between the EVO Multitask group and the EVO Words group. Change scores are defined as the score obtained on the pre-training assessment

subtracted from the post-training assessment (Post – Pre). Change score group means will be compared using a 2-tailed between participants T-Test with a base alpha criterion of 0.05 to meet statistical significance. Note: The assumptions for a T-Test will be checked and if the assumptions do not hold true, then other appropriate methods will be used as described in the SAP. Below are general formulations of the null and alternative statistical hypotheses,

- H_0: $\mu_{EVOMULTI} = \mu_{EVOWORDS}$ (1)
- H_1: $\mu_{EVOMULTI} \neq \mu_{EVOWORDS}$ (2)

where H_0 is the null hypothesis, H_1 is the alternative hypothesis, $\mu_{EVOMULTI}$ is the mean change score for the EVO Multitask group, and $\mu_{EVOWORDS}$ is the mean of the change score for the EVO Words group. The Primary Objective (P1) is a special case of this hypothesis test that will use weighted scores to control for Type-I error increases due to a sample size re-estimation and potential sample size increase at the interim analysis. The calculation of weighed scores will use the Chu-Hung-Wang method (Chang, 2014; Chu, Hung, & Wang, 1999).

Multiplicity will be controlled across the Major Objectives of this study (P1 and S1). See the SAP for details on multiplicity adjustments

8.6.2 Analysis Populations

Three populations are defined for this study: A per-protocol (PP), intention-to-treat (ITT), and safety population. The ITT and PP populations will be used for all efficacy analyses. The Safety population will be used for the safety analysis. The success of the primary and secondary objectives will be based on an assessment of the results from the ITT population. Additional analysis of the PP population is supportive. The primary efficacy endpoint must achieve significance in the ITT to preserved type I error for the secondary endpoints. Details about inclusion into each population are presented below:

- Per-Protocol (Treatment Complaint): This population is defined as any participant that began the at-home phase, completed both the pre and post assessment, and met a minimum compliance level of 50%. Additionally the following participants are excluded from this population:

- Participants where the subjective clinical assessments (ADHD-RS, CGI, and IRS) were not made by the same study staff member pre and post
- Participants where it was reported that the blinding was broken
- Participants that received incorrect intervention after randomization
- Participants who used prohibited/exclusionary medication or other treatments for ADHD during the course of the trial

- Intention-to-Treat (ITT): This population is defined as any randomized participant that received a device for at home training. In the case participants received the incorrect intervention after randomization, no corrections to group assignment will be made.

- Safety: This population is defined as any randomized participant that received a device for at home training. The primary safety analyses will include participants regardless of if they completed the post-treatment assessment and will be analyzed in the group whose treatment they actually received.

8.6.3 Interim Analyses

An interim analysis will be conducted on the primary outcome, TOVA 8 API, to determine if the minimum sample size of 300 patients is sufficient to detect a statistical difference between EVO Multitask and EVO Words. The population for this analysis will follow the same guidelines used for inclusion into the ITT population (See section 8.6.2).

The specific results of the interim analysis will remain blinded and will not be used to declare success of the primary objective at the interim; Un-blinded staff at the DCRI will conduct this analysis for the purpose of finalizing the sample size only. To maintain blinding for all other study personnel and affiliates, the results of this analysis will only indicate the sample size necessary to sustain the power of the study to detect an effect size of 0.4. Enrollment in the study will continue until the at least a minimum of 300 patients have been enrolled and the study has achieve the number of enrolled patients necessary to sustain the power of the study. The study will not exceed 1,000 enrolled patients. Additional details on the interim analysis will be presented in the SAP.

8.6.4 Power and Sample Size

To determine an appropriate study sample size to detect a statistical difference between change scores of the EVO Multitask group vs. the EVO Words group, a preliminary power analysis was conducted. The results indicated that 150 participants per group is sufficient to detect an effect size of 0.40 at a power > 90% on a between participants T-test with an alpha criterion of 0.05.

9. DATA AND SAFETY MONITORING

9.1 Endpoint Determination

API scores from the TOVA 8 will be derived directly from the computers used to administer the task and these scores will be entered into the database for analysis of primary outcome

9.2 Safety Monitoring

Safety/tolerability will be assessed primarily at the Post-Treatment Visit (Visit 3), as well as during the 4-week game play component as necessary.

9.3 Data Management to Maintain Blinding

As noted above (Section 4.7), instructions will be given to participants, their parents and study staff to minimize the possibility that information that may potentially unblind treatment assignment (e.g., *parent or participant description of their assigned game content)* is limited.

10 DATA MONITORING AND QUALITY CONTROL

10.1 Required Data

The full study dataset will be collected for participants who enter the treatment phase of the study. Limited data (i.e., demographics, adverse events, selection criteria, and reason for discontinuation) will be collected for participants who discontinue before the treatment phase.

All required data for this study will be entered into the electronic case report form (eCRF).

10.2 Subject and Clinical Data

The collection site will provide the following

This study will use Web-based e-CRFs developed through a validated, electronic reporting-electronic-signatures–compliant platform (21 Code of Federal Regulations Part 11). Before initiation of the trial, each site will be contacted as to computer availability, hardware specifications, and Internet connectivity, to evaluate the capacity of the site to use this type of data collection system. The investigator's site staff who will be entering data will receive training on the system, after which each person will be issued a unique user identification (ID) and password.

For security reasons, and in compliance with regulatory guidelines, it is imperative that only the persons who own the user IDs and passwords access the system using their own unique access codes. Access codes are nontransferable. Site personnel who have not undergone training may not use the system and will not be issued user ID and password until appropriate training is completed.

During monitoring visits, the site will make their computer and/or high-speed Internet access available to the clinical research associate. At the conclusion of the study, each enrolling site will be provided with a compact disc containing PDF files of both the individual subject's data and the audit trail (changes made to the database). This will be maintained at the site according to the requirements for records retention.

11 STUDY RESPONSIBILITIES

11.1 Study Data Reporting and Processing

Each page of the electronic case report form (eCRF) will be reviewed by site PI at the site. The site PI is required to sign the eCRF on the appropriate pages to verify that he or she has reviewed the recorded data. This review and sign-off may be delegated to a qualified clinician appointed as a sub investigator by the site PI. The transfer of duties to a sub investigator will be recorded on the delegation list (kept on file at the site). The investigator must ensure that all site staff involved in the conduct of the trial are familiar with the protocol and all study-specific procedures, and that they have appropriate knowledge of the study agents.

11.2 Training

The training of appropriate clinical site personnel across study procedures will be the responsibility of Akili Interactive Labs, Inc. and/or their representative(s) (EVO Multitask and EVO Words, CANTAB, TOVA, compliance monitoring) and DCRI (Protocol procedures, MINI-KID, ADHD-RS, eCRF/data entry, AE reporting, general test administration). The site PI is responsible for ensuring that his or her staff conduct the study according to the protocol. To ensure proper administration of study interventions, uniform data collection, and protocol compliance, Akili Interactive Labs, Inc. and their representatives and DCRI will present a formal training session to study site personnel, to include instructions for study procedures, the investigational plan, instructions on data collection, methods schedules for follow-up with the study site coordinators, and regulatory requirements. Detailed feedback regarding completion of forms will be provided by Akili Interactive Labs, Inc. and their representatives and DCRI in the course of regular site monitoring.

11.3 Monitoring the Investigational Sites

As part of a concerted effort to follow the study in a detailed and orderly manner in accordance with established principles of GCP and applicable regulations, a study monitor will visit the study sites regularly and will maintain frequent telephone and written communication. Additional information regarding site management and monitoring can be found in the Clinical Monitoring Plan (CMP).

During monitoring visits, the monitor will review informed consent, and HIPAA authorization, all inclusion/exclusion criteria, all events meeting criteria for expedited event reporting, as well as safety and efficacy endpoints. Additional review will be performed on a site-by-site basis, as warranted by the findings of previous monitoring visits. Key variables (demographics, inclusion/exclusion

criteria, and safety) on the eCRFs will be compared with each subject's source documents. Any discrepancies will be noted and resolved.

11.4 Study Documentation

Study documentation includes all electronic case report forms, source documents, monitoring logs and appointment schedules, sponsor-investigator correspondence, and regulatory documents (e.g., signed protocol and amendments, IRB or EC correspondence and approval, approved and signed subject consent forms, Statement of Investigator form, clinical supplies receipts and distribution records).

The site PI is responsible for ensuring that complete and accurate is prepare and maintain in compliance with GCP standards and applicable federal, state, and local laws, rules and regulations; and, for each subject participating in the study, promptly complete all eCRFs and such other reports as required by this protocol following completion or termination of the clinical study or as otherwise required pursuant to any agreement with the sponsor or their designated representative.

By signing the protocol, the site PI acknowledges that, within legal and regulatory restrictions and institutional and ethical considerations, study documentation will be promptly and fully disclosed to the sponsor or their designated representative by the investigator upon request and also shall be made available at the investigator's site upon request for inspection, copying, review, and audit at reasonable times by representatives of the Sponsor and the sponsor or their designated representative, or responsible government agencies as required by law.

The site PI agrees to promptly ensure that any reasonable steps that are requested by Sponsor or the sponsor or their designated representative as a result of an audit to cure deficiencies in the study documentation and eCRFs are taken.

11.5 Protocol Deviations

A protocol deviation is defined as an event where the investigator or site personnel did not conduct the study according to the investigational plan or the Investigator Agreement.

Investigators are required to obtain approval from the sponsor or their designated representative medical monitor before initiating deviations from the investigational plan or protocol, except where necessary to protect the life or physical well-being of a subject in an emergency. Such approval will be documented in writing and maintained in study files. Unless the Sponsor has consented to any such deviations in writing, Sponsor will not assume any resulting responsibility or liability. Preapproval is generally not expected in situations where unforeseen circumstances are beyond the investigator's control,

(e.g., subject did not attend scheduled follow-up visit,); however, the event is still considered a deviation.

The IRB will be informed of all protocol changes by the sponsor or deviations that occur at the investigative site in accordance with applicable regulations and the IRB/EC's established procedures. No deviations from the protocol of any type will be made without complying with all the IRB/EC's established procedures.

11.6 Study Supply Accountability

The investigator will maintain records of the receipt and disposition of all investigational devices (i.e., tablets), including any reported instances of unintended usage. When the enrollment is complete, the investigator will be notified by the sponsor or their designated representative, and in a timely manner, will return all study devices as directed by the Sponsor.

11.7 Data Transmittal and Record Retention

Required data will be entered in the eCRF as soon as possible after the subject visit or the availability of test results, but no later than 3 business days after the completed visit.

Study sites will transcribe subject source data into eCRFs using a computerized electronic data capture (EDC) system. The EDC system is compliant with all relevant aspects of GCP. Edit checks, electronic queries, and audit trails are built into the system to ensure accurate and complete data collection. Data will be transmitted via the Internet from investigational sites to a central site, utilizing state-of-the-art encryption mechanisms to ensure security and confidentiality.

The eCRF will be transmitted to a secure central database (host site) as forms are completed or updated. Protocol-specified source documents will be retrieved as necessary. Originals of all study-related documentation will be retained at the site.

The site PI will maintain the records of device/tablet disposition, final eCRFs, worksheets, and all other study-specific documentation (e.g., study file notebooks or source documentation) until notified by the sponsor that records may be destroyed. To avoid error, the investigator will contact the sponsor or their designated representative before the destruction of any records pertaining to the study to ensure they no longer need to be retained. In addition, the sponsor will be contacted if the site PI plans to leave the institution so that arrangements can be made for the transfer of records.

11.8 Study Closeout

Upon completion of the study (defined as all participants have completed all follow-up visits, all eCRFs are complete, and all queries have been resolved), the sponsor or their designated representative will notify the site of closeout, and a study closeout visit will be performed. All devices will be collected and returned to the sponsor or their designated representative. The sponsor or their designated representative monitor will ensure that the site PI's regulatory files are up-to-date and complete and that any outstanding issues from previous visits have been resolved. Other issues to be reviewed at the closeout visit include discussing retention of study files, possibility of site audits, publication policy, and notifying the IRB of study closure.

11.9 Audit/Inspections

The sponsor or their designated representative quality assurance personnel may conduct audits at the study sites. Audits will include, but not be limited to, audit trail of data handling and processes, standard operating procedures, device supply, presence of required documents, the informed consent process, and comparison of eCRFs with source documents. The site PI agrees to accommodate and participate in audits conducted at a reasonable time and in a reasonable manner, as needed.

The FDA may also inspect the site during or after the study. The site PI should contact the sponsor immediately if this occurs and must fully cooperate with governmental (e.g., FDA) inspections conducted at a reasonable time and in a

12. REFERENCES

Biederman, J, et al. Evidence of a pharmacological dissociation between the robust effects of methylphenidate on ADHD symptoms and working memory. Journal of Brain Science 2015; 1(2) 43-53

Klinberg T. Training and Plasticity of Working Memory. Trends in Cognitive Sciences. 2010; 14(2) 317-314

Cortese, S, et al. Cognitive Training for Attention Deficit/Hyperactivity Disorder; Meta-Analysis of Clinical and Psychological Outcomes from Randomized Controlled Trials. Journal of American Academy of Child and Adolescent Psychiatry 2015; 54(3) 164-174.

Anguera, J.A., et al. Video Game Training Enhances Cognitive Control in Older Adults. Nature 2013; 501, 97-101.

Blackwelder WC. "Proving the null hypothesis" in clinical trials. Control Clin Trials. 1982;3:345–53
