# Supplementary material for: Secondary analyses of sex differences in attention improvements across three clinical trials of a digital therapeutic in children, adolescents, and adults with ADHD
Source: BMC Public Health. 2024 Apr 29;24:1195. doi: 10.1186/s12889-024-18597-5 (PMC11057090; doi:10.1186/s12889-024-18597-5)
Supplement: Supplementary file 2 — Supplementary Material 2. [file 12889_2024_18597_MOESM2_ESM.docx]

### Supplemental

#### Supplemental Baseline Measures

There were no significant sex differences in age for the child or adult samples (child: *M(SD)female* 9.5(1.32), *M(SD)male* 9.80(1.32), p=0.18; adult: *M(SD)female* 39.598 (12.791), *M(SD)male* 40.478 (13.057), p=0.699)). Girls were slightly older than boys in the adolescent sample (adolescent: *M(SD)female* 14.583 (1.394), *M(SD)male* 14.163 (1.126) p=0.046; See Supplemental Table 1).

There were significantly more boys than girls in the child and adolescent samples (child: *n* female=55, *n* male=125, *X2* = 27.222(1); adolescent: *n* female=60, *n* male=86, p <.001; *X2* = 4.6301(1), p = 0.03142, but significantly more women than men in the adult sample (adult: *n* female=107, *n* male=46 *X2* = 24.32(1), p <.001; See Supplemental Table 1).
